# Anti-Inflammatory Effect of Xanthones from *Hypericum beanii* on Macrophage RAW 264.7 Cells through Reduced NO Production and TNF-*α*, IL-1*β*, IL-6, and COX-2 Expression

**DOI:** 10.3390/molecules29153705

**Published:** 2024-08-05

**Authors:** Wei Ma, Fu-Cai Ren, Xue-Ru Wang, Ning Li

**Affiliations:** School of Pharmacy, Anhui Medical University, No.81 Meishan Road, Shushan District, Hefei 230032, China; mw421553449@sina.com (W.M.); renfucai@ahmu.edu.cn (F.-C.R.); 19855867686@139.com (X.-R.W.)

**Keywords:** *Hypericum beanii*, chemical constituents, xanthones, anti-inflammatory activity

## Abstract

*Hypericum beanii* N. Robson, a perennial upright herb, predominantly inhabits temperate regions. This species has been utilized for the treatment of various inflammation-related diseases. One new xanthone 3,7-dihydroxy-1,6-dimethoxyxanthone (**1**) and twenty-three known xanthones (**2**–**24**) were isolated from the aerial parts of *H. beanii*. The structure of the new compound was determined based on high-resolution electrospray ionization mass spectroscopy (HR-ESIMS), nuclear magnetic resonance (NMR), Infrared Spectroscopy (IR), ultraviolet spectrophotometry (UV) spectroscopic data. The anti-inflammatory effects of all the isolates were assessed by measuring the inhibitory effect on nitric oxide (NO) production in LPS-stimulated RAW 264.7 macrophages. Compounds 3,4-dihydroxy-2-methoxyxanthone (**15**), 1,3,5,6-tetrahydroxyxanthone (**19**), and 1,3,6,7-tetrahydroxyxanthone (**22**) exhibited significant anti-inflammatory effects at a concentration of 10 *μ*M with higher potency compared to the positive control quercetin. Furthermore, compounds **15**, **19**, and **22** reduced inducible NO synthase (iNOS), tumor necrosis factor alpha (TNF-*α*), interleukin-1*β (*IL-1*β*), IL-6, and cyclooxygenase 2 (COX-2) mRNA expression in the LPS-stimulated RAW 264.7 macrophages, suggesting that these compounds may mitigate the synthesis of the aforementioned molecules at the transcriptional level, provisionally confirming their anti-inflammatory efficacy.

## 1. Introduction

The genus *Hypericum*, a member of the Hypericaceae family, encompasses over 500 taxa distributed across temperate regions in the northern hemisphere and high-altitude tropical areas worldwide [1]. In southwestern China, there are approximately fifty-five species and eight subspecies of this genus. More than 20 species from *Hypericum* have been utilized in traditional Chinese medicine [2]. Among them, *Hypericum beanii* N. Robson, a perennial upright herb primarily found in temperate regions such as Kunming, Lunan, and Mengzi in Yunnan Province, China, is locally known as “Huang-hua-xiang” [3]. *H. beanii* has been traditionally used for its therapeutic properties including heat clearing (reducing or eliminating heat-related imbalances in the body) and detoxification effects, as well as relaxation of tendons and activation of collaterals to address various ailments, such as menstrual fever, epistaxis, jaundice due to damp-heat (a pathological condition where there is an accumulation of dampness (Shi) and heat (Re) in the body), arthralgia, myalgia, upper respiratory infections, hepatitis, nephritis, and stomatitis [4,5]. Recent reports have identified the presence of several phenolic compounds in *H. beanii*, including xanthones, flavones, and phloroglucinols. Among them, polycyclic polyprenylated acylphloroglucinols (PPAPs) have exhibited hepatoprotective, cytotoxic, and anti-inflammatory activities in vitro [6,7,8].

Inflammation is a defense response of living tissues to injurious stimuli and a complex biological response by the organism to remove the stimulus and then initiate the tissue healing process [9]. Macrophages play a crucial role in the response to inflammatory stimuli by secreting a series of pro-inflammatory cytokines, signaling proteins, and various other inflammatory mediators, including TNF-*α*, IL-1*β*, and IL-6 [10]. NO regulated by iNOS reacts with peroxides to promote the inflammatory processes [11]. The enzyme responsible for catalyzing the conversion of arachidonic acid into prostaglandin G2 (PGG2) and subsequently into PGH2 is known as COX, or prostaglandin-endo peroxide synthase. There are two mammalian isozymes encoded by different genes: the constitutive COX-1 and the inducible COX-2. Both enzymes are major targets of NSAIDs in pharmacology [12].

**Figure 1:**
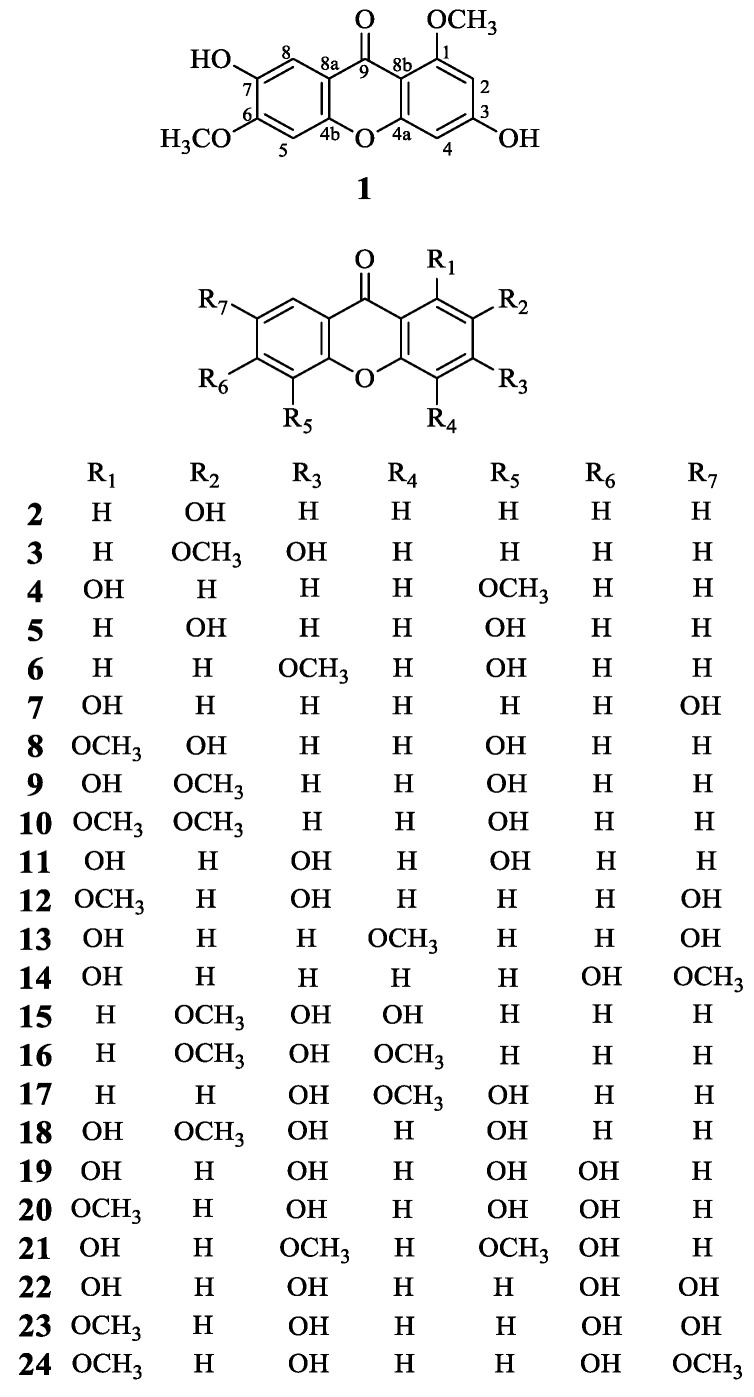
Structures of compounds **1**–**24**.

Xanthones possess a unique 9*H*-Xanthen-9-one scaffold, mainly found in the plants of the Gentianaceae and Hypericaceae families. Xanthones showed a variety of pharmacological activities and structural diversity. Many xanthones have been reported with potent anti-inflammatory properties [13]. In order to find more xanthone compounds with anti-inflammatory activities from the aerial parts of *H. beanii*, in the present study, one new xanthone (**1**) and twenty-three known xanthone compounds (**2**–**24**) were obtained from the aerial parts of *H. beanii* (Figure 1). The anti-inflammatory properties of these compounds were evaluated based on their ability to inhibit NO production in LPS-stimulated RAW 264.7 macrophages. Compounds **15**, **19**, and **22** exhibited significant anti-inflammatory effects. Furthermore, the anti-inflammatory activity of compounds **15**, **19**, and **22** was confirmed through the inhibition of pro-inflammatory mediator production and other potentially associated anti-inflammatory signaling pathways.

## 2. Results and Discussion

### 2.1. Isolation and Structural Elucidation of Compounds **1**–**24**

**Figure 2:**
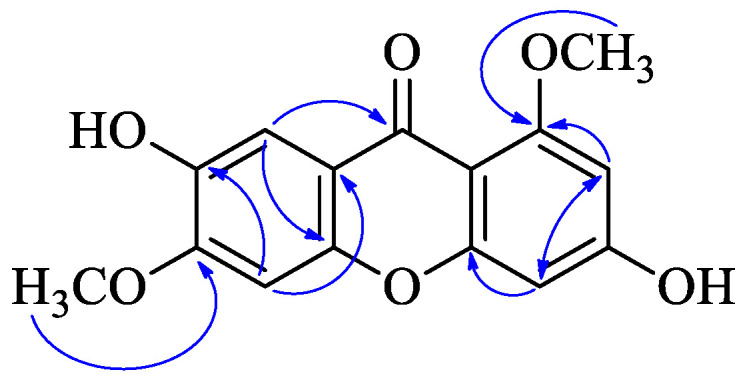
HMBC (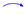) of compound **1**.

Compound **1** was obtained as a yellow powder. The molecular formula was established as C_15_H_12_O_6_ based on the quasi-molecular ion observed at *m*/*z* 287.0561 [M − H]^−^ (calcd 287.0561 for C_15_H_11_O_6_^−^) by HR-ESI-MS. The IR spectrum showed absorption bands at *ν*_max_ 3343 and 1613 cm^−1^, corresponding to hydroxy groups and an aromatic conjugated carbonyl group, respectively. The ^1^H-NMR spectrum showed two methoxyl groups [*δ* 3.80 (3H, s) and 3.88 (3H, s)] and four singlet aromatic protons [*δ* 6.33 (1H, s), 6.37 (1H, s), 7.01 (1H, s), and 7.34 (1H, s)] (Table 1). ^13^C-NMR spectrum of compound **1** showed 14 carbon signals, which were sorted by HMQC techniques as two methoxyl groups, four methines, and nine carbons without hydrogens attached, including a carbon of a carbonyl group (172.7 ppm), six oxygenated sp^2^ carbons. The ^1^H and ^13^C NMR spectra of compound **1** were similar to compound **22** (1,3,6,7-tetrahydroxyxanthone) except two methoxyl groups of *δ*_H_ 3.80 (s). *δ*_H_ 3.88 (s), *δ*_C_ 55.8, and *δ*_C_ 56.1. The methoxy groups were connected at positions C-1 and C-6, which was confirmed by the HMBC correlations from *δ*_H_ 3.80 (s) to C-1, and *δ*_H_ 3.88 (s) to C-6, respectively (Figure 2). Thus, the structure of compound **1**, 3,7-dihydroxy-1,6-dimethoxyxanthone, was assigned as shown in Figure 1.

The known compounds (**2**–**24**) were identified by comparing their experimental NMR spectral data with those in the literature. These compounds were identified as 2-hydroxyxanthone (**2**) [14], 3-hydroxy-2-methoxyxanthone (**3**) [15], 1-hydroxy-5-methoxyxanthone (**4**) [16], 2,5-dihydroxyxanthone (**5**) [17], 5-hydroxy-3-methoxyxanthone (**6**) [18], 1,7-dihydroxyxanthone (**7**) [19], 2,5-dihydroxy-1-methoxyxanthone (**8**) [20], 1,5-dihydroxy-2-methoxyxanthone (**9**) [21], 5-hydroxy-1,2-dimethoxyxanthone (**10**) [22], 1,3,5-trihydroxyxanthone (**11**) [23], 3,7-dihydroxy-1-methoxyxanthone (**12**) [24], 1,7-dihydroxy-4-methoxyxanthone (**13**) [25], 1,6-dihydroxy-7-methoxyxanthone (**14**) [23], 3,4-dihydroxy-2-methoxyxanthone (**15**) [26], 3-hydroxy-2,4-dimethoxyxanthone (**16**) [27], 3,5-dihydroxy-4-methoxyxanthone (**17**) [28], 1,3,5-trihydroxy-2-methoxyxanthone (**18**) [29], 1,3,5,6-tetrahydroxyxanthone (**19**) [30], 3,5,6-trihydroxy-1-methoxyxanthone (**20**) [31], 1,6-dihydroxy-3,5-dimethoxyxanthone (**21**) [32,33], 1,3,6,7-tetrahydroxyxanthone (**22**) [34], 3,6,7-trihydroxy-1-methoxyxanthone (**23**) [35], and 3,6-dihydroxy-1,7-dimethoxyxanthone (**24**) [36]. It is noteworthy that compounds **2**, **4**–**9**, **11**–**15**, and **18**–**24** were reported from *H. beanii* for the first time.

### 2.2. Cell Viability

**Figure 3:**
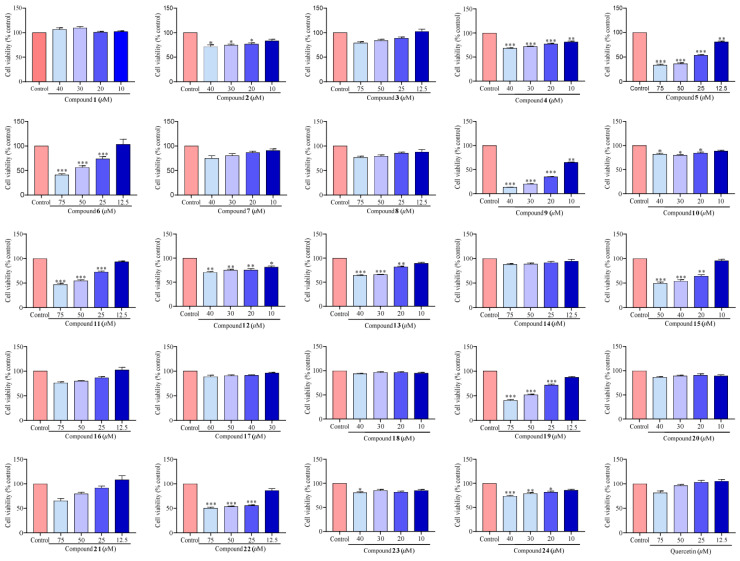
The cytotoxic effects of compounds (**1**–**24**) on the RAW 264.7 macrophage cells. Values are the mean ± SEM, n = 3. (Compared to the control group *** p < 0.001, ** *p* < 0.01, or * *p* < 0.05).

The potential cytotoxicity of compounds (**1**–**24**) on RAW 264.7 macrophage cells was evaluated through Cell Counting Kit-8 (CCK-8, Apexbio, Houston, TX, USA). The results were compared to a control group incubated with normal medium only. As depicted in Figure 3, the majority of compounds exhibited no significant cytotoxic effects at a concentration of 10 *μ*M. Consequently, we deduced that the concentration of 10 *μ*M for further analyses would be appropriate.

### 2.3. Evaluation of Anti-Inflammatory Activity of the Isolated Compounds

Previous reports have shown many models, either in vitro *or* in vivo, which are recruited to evaluate the anti-inflammatory properties of xanthones. A lot of xanthones with anti-inflammatory properties have been found, including simple oxygenated xanthones, xanthone glycosides, prenylated xanthones, and xanthonolignoids [13]. Nitric oxide (NO) is a short-lived molecule produced by the enzyme nitric oxide synthase (NOS) and is released by macrophages in response to pathogens. In many diseases, high levels of NO are produced due to the induction of iNOS [37]. Increased production of NO has been considered an indication of macrophage activation, making it a well-known method for investigating anti-inflammatory properties [38].

**Figure 4:**
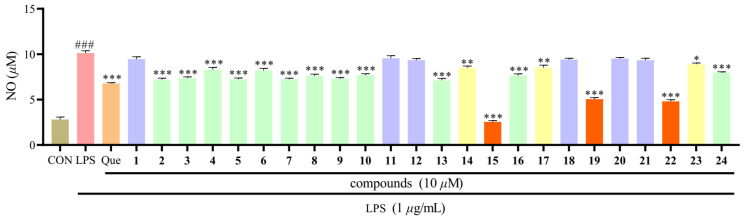
The inhibitory effects of **1**–**24** against LPS-induced NO production in RAW264.7 macrophages. Mean ± SEM of three replicates is shown. * *p* < 0.05, ** *p* < 0.01, *** *p* < 0.001 with the LPS group. ^###^ *p* < 0.001 with the CON (control) group.

We have separated the chemical components from the aerial parts of *H. beanii*, in order to find more xanthone compounds with anti-inflammatory activities and to promote the reasonable use of this herb as a medicinal plant. In this study, all xanthone compounds from the aerial parts of *H. beanii* were tested for their inhibitory activities at 10 *μ*M against NO production in LPS-induced RAW264.7 macrophages (Figure 4). Except for compounds **1**, **11**, **12**, **18**, **20,** and **21**, other components showed varying degrees of anti-inflammatory activity. Compounds **15**, **19**, and **22** displayed significant anti-inflammatory effects with higher potency compared to the positive control quercetin.

All the compounds **1**–**24** isolated from *H. beanii* were analogues and possessed the same xanthone skeleton with different amounts of -OCH_3_ and -OH groups at C-1 to C-7 positions. Compounds **1** and **22**–**24** have substituents in four identical positions of C-1, C-3, C-6, and C-7, but **1**, **23,** and **24** displayed much weaker anti-inflammatory activity than **22**. Careful further molecular structure analysis allowed us to reach the preliminary deduction that the anti-inflammatory activity of compound was reduced when -OH was substituted by -OCH_3_ at the same position. Similar conclusions are observed between compounds **15** and **16**, as well as between compounds **19** and **20**, and **19** and **21**. This result suggested that the number of phenolic hydroxyl groups is a key factor in the enhancement of the anti-inflammatory activity of xanthone when the number and position of substituents are exactly the same.

### 2.4. Effect of Compounds **15**, **19**, and **22** on NO Production and the mRNA Level of iNOS

**Figure 5:**
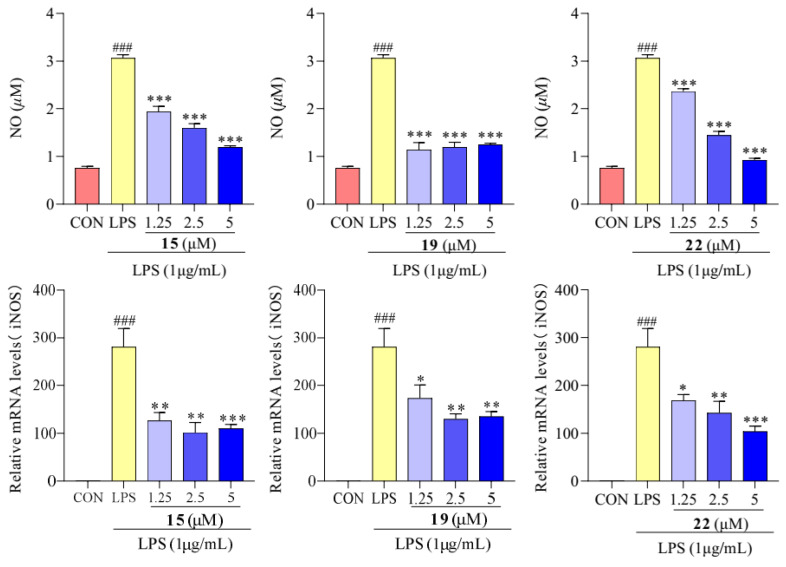
Concentrations of NO and mRNA expression of iNOS in RAW 264.7 cells treated with compounds **15**, **19**, and **22**. Values are the mean ± SEM, n = 3. (Compared to the control group ^###^ *p* < 0.001. Compared to the LPS group, *** *p* < 0.001, ** *p* < 0.01, or * *p* < 0.05).

As shown in Figure 5, the concentrations of NO and mRNA expression level of iNOS were higher in the LPS treatment group than in the control group, indicating an immune inflammatory reaction. And treatment with compounds **15**, **19**, and **22** resulted in a significantly lower NO content compared to that in LPS-treated cells. The expression level of iNOS mRNA, when treated with compounds **15**, **19**, and **22**, showed a noticeable difference from the LPS group. It can be speculated that compounds **15**, **19**, and **22** inhibit NO synthase by suppressing iNOS expression, ultimately reducing NO production.

Many plants of the *Hypericum* genus containing xanthones as bioactive constituents have been traditionally used as anti-inflammatory agents [13,39]. Among the xanthone derivatives tested, compounds **15**, **19**, and **22** showed a remarkable inhibitory effect, suggesting its potential anti-inflammatory activity. Previous studies have highlighted the ability of these compounds to modulate inflammatory pathways, including the inhibition of NO production [39]. In this study, the observed inhibitory effects suggest that compounds **15**, **19**, and **22** may interfere with the inflammatory cascade by suppressing NO production.

### 2.5. Effect of Compounds **15**, **19**, and **22** on TNF-α, IL-1β, and IL-6

Macrophage activation can increase the production of cytokines such as TNF-*α*, IL-1*β*, and IL-6. Overproduction and release of these cytokines can disrupt the immune balance of the body, leading to an exaggerated immune response and contributing to the development of inflammatory diseases [40]. Therefore, the anti-inflammatory activity of compounds **15**, **19**, and **22** on TNF-*α*, IL-1*β*, and IL-6 levels was analyzed to assess their effects on the pathways involving these cytokines.

**Figure 6:**
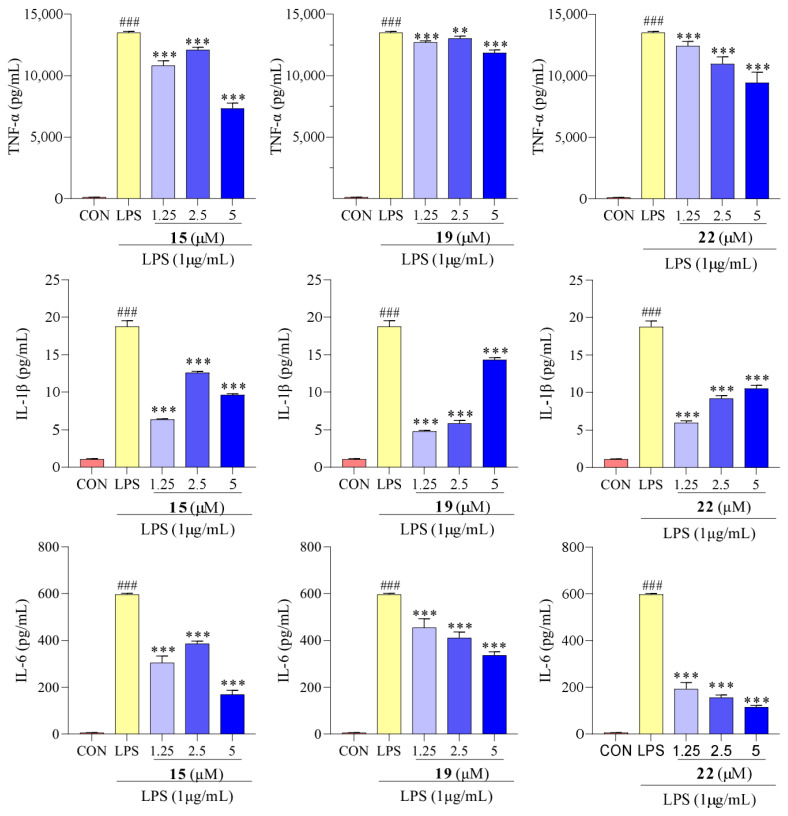
Concentrations of TNF-*α*, IL-1*β*, and IL-6 in RAW 264.7 cells with compounds **15**, **19**, and **22** treatments. Values are the mean ± SEM, n = 3. (Compared to the control group ^###^ *p* < 0.001. Compared to the LPS group, *** *p* < 0.001, ** *p* < 0.01).

**Figure 7:**
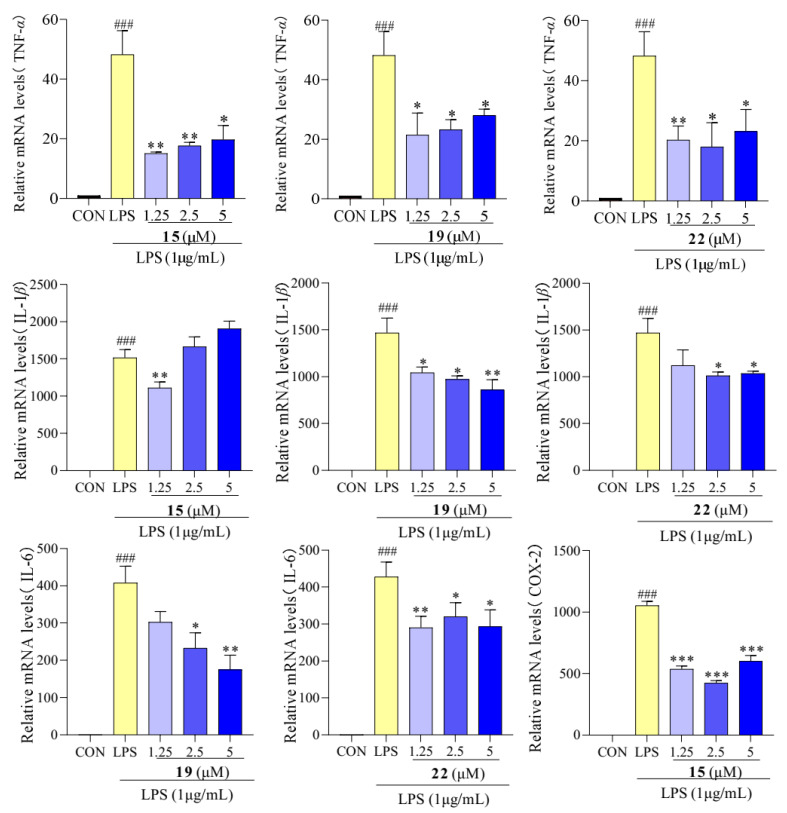
mRNA expression of pro-inflammatory cytokines TNF-*α*, IL-1*β*, and IL-6 in RAW 264.7 cells treated with compounds **15**, **19**, and **22**. Values are the mean ± SEM, n = 3. (Compared to the control group ^###^ *p* < 0.001. Compared to the LPS group, *** *p* < 0.001, ** *p* < 0.01, or * *p* < 0.05).

The results presented in Figure 6 show that the LPS-treated group exhibited a significant elevation in TNF-*α*, IL-1*β*, and IL-6 levels compared to the control group. However, after treatment with compounds **15**, **19**, and **22,** concentrations of TNF-*α*, IL-1*β*, and IL-6 were reduced. We determined the effect of compounds **15**, **19**, and **22** on the mRNA expression of TNF-*α*, IL-1*β*, and IL-6 using qPCR. As showed in Figure 7, the mRNA expression of TNF-*α*, IL-1*β*, and IL-6 increased upon LPS treatment, following the same trend as the secretion levels of TNF-*α*, IL-1*β*, and IL-6. Conversely, following treatment with compounds **15**, **19**, and **22**, the mRNA expression of TNF-*α*, IL-1*β*, and IL-6 in the macrophages decreased compared to the LPS-treated group, supporting our previous finding that treatment with compounds **15**, **19**, and **22** reduced the mRNA expression of TNF-*α*, IL-1*β*, and IL-6. Additionally, in previous studies, Cho et al. reported that mangostenone F, a natural xanthone, dose-dependently inhibited the production of NO, iNOS, and pro-inflammatory cytokines (TNF-*α*, IL-1*β*, and IL-6) in LPS-stimulated RAW264.7 cells [41]. Li et al. described that 1,3,6,7-tetrahydroxy-8-prenylxanthone was identified as a potent inhibitor of LPS-induced NO production and IL-6 secretion in RAW264.7 macrophages [42]. Jeong et al. found that cudratricusxanthone A, an isoprenylated xanthone, suppressed TNF-*α* and IL-1*β* production [43]. These results suggest that compounds **15**, **19**, and **22** inhibit the inflammatory activity in macrophage RAW264.7 cells via lowering the expression and secretion of the inflammatory mediators TNF-*α*, IL-1*β*, and IL-6.

### 2.6. Effect of Compounds **15**, **19**, and **22** on COX-2

**Figure 8:**
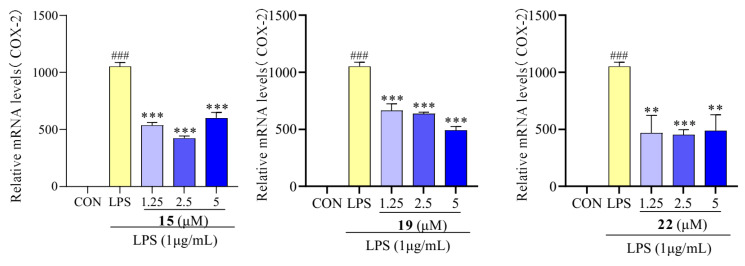
COX-2 mRNA expression in RAW 264.7 cells treated with compounds **15**, **19**, and **22**. Values are the mean ± SEM, n = 3. (Compared to the control group ^###^ *p* < 0.001. Compared to the LPS group, *** *p* < 0.001 or ** *p* < 0.01).

Recent reports have suggested that increased levels of COX activity promote inflammatory pain [44]. To investigate the inhibitory effect of compounds **15**, **19**, and **22** on LPS-induced inflammatory response in RAW 264.7 macrophages, the level of COX-2 mRNA expression was determined. After treatment with LPS, the mRNA expression levels of COX-2 were significantly increased, whereas following treatment with compounds **15**, **19**, and **22**, the mRNA expression of COX-2 significantly decreased compared to the LPS-treated group (Figure 8). Similar results were reported that suggest that ravenelin, a xanthone, suppressed iNOS and COX-2 expression in LPS-induced RAW 264.7 macrophages [45].

## 3. Materials and Methods

### 3.1. General Experimental Procedures

A Shimadzu UV-2401PC spectrophotometer was used to obtain the UV spectra. A Thermo NICOLET Is10 FT-IR spectrometer was used for IR spectroscopy with KBr pellets. The 1D and 2D NMR spectra were recorded on an Avance III-600 spectrometer with TMS as an internal standard, and chemical shifts (*δ*) are expressed in ppm. HR-MS was performed on an Agilent 1290 UPLC/6540 Q-TOF spectrometer (Agilent, Santa Clara, CA, USA). A JASCO J-1500 spectrometer was used to measure the ECD spectra (JASCO Corp., Japan). Sephadex LH-20 gel (25–100 *μ*m) was obtained from Pharmacia Fine Chemical (Uppsala, Sweden), and C18 silica gel (50 *μ*m) was procured from YMC (Osaka, Japan). Column chromatography silica gel (200–300 mesh) and thin-layer chromatography (TLC) silica gel plates were purchased from Shanghai Haohong Biomedical Technology (Shanghai, China). HPLC separations were carried out with a Thermo Scientific UltiMate3000 liquid chromatography system, and a Thermo Hypersil ODS column (ODS, 250 × 10 mm, 5 *μ*m; Thermo, Waltham, MA, USA) was used.

### 3.2. Plant Material

The aerial parts of *H. beanii* were collected from Kunming, Yunnan Province, People’s Republic of China, in June 2019 and were authenticated by Prof. Kai-Jin Wang (Anhui University). A voucher specimen (20190630-3) was deposited at the School of Pharmacy, Anhui Medical University.

### 3.3. Extraction and Isolation

The dried aerial parts of *H. beanii* (9.5 kg) were subjected to extraction with 95% ethanol (4 × 50 L) at room temperature to obtain 680 g of crude residue. The residue was resuspended in water and subsequently extracted with petroleum ether (PE) and EtOAc. Six fractions (Fr. 1 to Fr. 6) were obtained from the EtOAc (240 g) partition by silica gel column chromatography eluting with dichloromethane (CH_2_Cl_2_)/EtOAc (from 100:1 to 1:1) and EtOAc/methyl alcohol (MeOH) (from 20:1 to 1:1). Fr. 4 (2.3 g) was fractionated by a Sephadex LH-20 column eluted with CH_2_Cl_2_/MeOH (1:1) to obtain two subfractions (Fr. 4.1 and Fr. 4.2). Fr. 4.1 (1.6 g) and Fr. 4.2 (380 mg) were separated by a Sephadex LH-20 column eluted with MeOH/H_2_O (from 10% to 100%) to yield Frs. 4.1.1–4.1.2, Frs. 4.2.1–4.2.3. Fr. 4.1.1 and Fr. 4.1.2 were further applied to C18 eluting with MeOH/H_2_O (from 10% to 100%) to yield Frs. 4.1.1.1–4.1.1.2 and Frs. 4.1.2.1–4.1.2.2. Fr. 4.1.1.1 was purified by preparative TLC (CH_2_Cl_2_/MeOH, 30:1) to produce **3** (65 mg). Fr. 4.1.1.2 was purified by CC eluting with CH_2_Cl_2_/EtOAc (80:1) to produce **6** (8 mg) and **9** (9 mg). Fr. 4.1.2.2 was further purified by preparative TLC (CH_2_Cl_2_: MeOH 30:1) to produce **7** (37 mg), **14** (4 mg), and **21** (8 mg). Frs. 4.2.1–4.2.3 were purified by preparative HPLC with MeOH/H_2_O (60:40) to afford **2** (Rt 23.5 min, 13.5 mg), **4** (Rt 26.2 min, 9 mg), **10** (Rt 28.2 min, 3 mg), **13** (Rt 13.8 min, 12 mg), **16** (Rt 22.5 min, 5.5 mg), and **18** (Rt 18.8 min, 6 mg). Fr.5 (8.85 g) was subjected to Sephadex LH-20 column elution with CH_2_Cl_2_/MeOH (1:1), and then separated by CC with CH_2_Cl_2_/EtOAc (20:1 to 1:1) and purified by a Sephadex LH-20 column to yield **5** (28 mg), **8** (26 mg), **11** (45 mg), and **17** (7 mg). Fr. 6 (45 g) was further applied to Sephadex LH-20 column elution with CH_2_Cl_2_/MeOH (1:1) to obtain two subfractions (Frs. 6.1–6.2). Fr. 6.1 (12 g) and Fr. 6.2 (2.03 g) were separated on a Sephadex LH-20 column eluted with MeOH to yield Frs. 6.1.1–6.1.2 and Frs. 6.2.1–6.2.2. Fr. 6.1.2 was separated by a Sephadex LH-20 column with MeOH/H_2_O (from 10% to 100%) to obtained Frs. 6.1.2.1–6.1.2.4. Fr. 6.1.2.1 was purified by CC with CH_2_Cl_2_/EtOAc (1:1) to produce **15** (12 mg). Fr. 6.1.2.2 and Fr. 6.1.2.3 were separated by CC with CH_2_Cl_2_/MeOH (20:1) and purified by preparative TLC (CH_2_Cl_2_/MeOH, 30:1) to produce **20** (17 mg), **23** (20 mg). Fr. 6.1.2.4 was purified by preparative HPLC with MeOH/H_2_O (37.5%) to afford **1** (Rt 11.9 min, 6 mg), **12** (Rt 10.8 min, 8 mg), **24** (Rt 9.8 min, 5 mg). Fr. 6.2.1 and Fr. 6.2.2 were separated by CC with CH_2_Cl_2_/MeOH (20:1) to produce **19** (5 mg), **22** (4 mg).

### 3.4. Spectroscopic Data

3,7-Dihydroxy-1,6-dimethoxyxanthone (**1**): C_15_H_12_O_6_, yellow powder; [*α*${]}_{D}^{25}$ + 73.3 (*c* 0.10, MeOH); UV (MeOH) *λ*_max_ (log *ε*) 250 (3.50), 302 (3.12), and 356 (2.88) nm; IR (KBr) *v*_max_ 3343.9, 1613.1, 1559.0, 1455.1, 1277, 1206.3, 1175.4, 1123.1, 1098.1, 1022.9, 965.2, 816.2, and 572.4 cm^−1^; HR-ESI-MS *m*/*z* 287.0561 [M − H]^−^ (calcd 287.0561 for C_15_H_11_O_6_^−^); ^1^H NMR (600 MHz) and ^13^C NMR (125 MHz) data; see Table 1.

### 3.5. CCK-8 Assay

Cell viability was examined by the CCK-8 assay [46], and none of the test compounds exhibited cytotoxicity at their effective concentrations. Quercetin was used as a positive control.

### 3.6. Anti-Inflammatory Activity

The inhibitory effects of all the compounds on LPS-stimulated NO production were evaluated in RAW264.7 macrophages by Griess reaction [46]. The cells (Cell Bank of the Chinese Academy of Sciences, Shanghai, China) were cultured in DMEM (Gibco, Pleasanton, CA, USA) supplemented with 10% FBS (Rongye Biotechnology, Lanzhou, China), 100 U/mL penicillin, and 100 U/mL streptomycin (Kaiji Biotechnology, Shanghai, China), and incubated at 37 °C in a humidified atmosphere with 5% CO_2_. The RAW 264.7 cells were seeded on 24-well plates at 1 × 10^5^ cells/well and incubated for 24 h. Then, they were treated with compounds and quercetin (10 *μ*M) with LPS (1 *μ*g/mL). After 24 h, 50 *μ*L of cell-free supernatant was mixed with 100 *μ*L of Griess reagent and incubated at room temperature for 5 min. The concentration of nitrite was measured at 540 nm. Sodium nitrite was used as a standard to calculate the NO concentration.

### 3.7. Determination of NO, TNF-α, IL-1β, and IL-6 Levels

The macrophages (1 × 10^5^ cells/mL) were treated with different concentrations of compounds **15**, **19**, and **22** (0, 1.25, 2.5, 5 *μ*M) with LPS (1 *μ*g/mL) or DMEM. After 24 h, cell supernatants were then collected and the levels of NO, TNF-*α* (ABclonal Technology, Wuhan, China, Cat. NO.: RK00027), IL-1*β* (ABclonal technology, Cat. NO.: RK04878), and IL-6 (ABclonal technology, Cat. NO.: RK00008) were measured by Griess reagent and ELISA kits [40].

### 3.8. qPCR Analysis

The RAW 264.7 cells were seeded on 12-well plates at 1 × 10^6^ cells/well and incubated for 24 h. Then, they were treated with compounds **15**, **19**, and **22** (0, 1.25, 2.5, 5 *μ*M) with LPS (1 *μ*g/mL) or DMEM [39]. After 24 h, cells were lysed to isolate total RNA. Total RNA was isolated using TRIzol reagent according to the manufacturer’s protocol for cDNA synthesis by reverse transcriptase. The cDNA encoding iNOS, TNF-*α*, IL-1*β*, IL-6, and COX-2 genes was quantified by a quantitative real-time PCR assay (qPCR) using gene-specific primers (Table 2). GAPDH was used as an internal reference.

### 3.9. Statistical Analysis

All the data were presented as mean ± SEM. One-way ANOVA tests were conducted using SPSS Statistics 20.0 for the comparison between treatments. A value of <0.05 was considered statistically significant.

## 4. Conclusions

One new xanthone (**1**) and twenty-three known xanthone compounds (**2**–**24**) were isolated from the aerial parts of *H. beanii*, and their chemical structures were elucidated through comprehensive spectroscopic analysis in conjunction with HR-ESI-MS. All xanthone compounds were tested for their inhibitory activities at 10 *μ*M against NO production in LPS-induced RAW264.7 macrophages. Except for compounds **1**, **11**, **12**, **18**, **20,** and **21**, other components showed varying degrees of anti-inflammatory activity. Notably, compounds **15**, **19**, and **22** exhibited significant anti-inflammatory activity by inhibiting NO production in LPS-stimulated murine macrophage RAW 264.7 cells. Furthermore, compounds **15**, **19**, and **22** reduced iNOS, TNF-*α*, IL-1*β*, IL-6, and COX-2 mRNA expression in the LPS-stimulated RAW 264.7 macrophages. Collectively, these findings underscore the potential of xanthones as crucial anti-inflammatory constituents derived from *H. beanii*; this notion is further supported by the inhibitory effects observed for compounds **15**, **19**, and **22** on tested inflammatory mediators. These results will contribute to a more rational utilization of both *H. beanii* and its constituent xanthones.

## Figures and Tables

**Table 1:** ^1^H and ^13^C NMR (600, 150 MHz) data of compound **1** in DMSO-*d*_6_.

NO	1	
	*δ*_C_	*δ*_H_
1	161.6	
2	94.8	6.33s
3	163.6	
4	95.6	6.37s
4a	159.0	
4b	143.7	
5	99.5	7.01s
6	153.4	
7	149.0	
8	108.8	7.34s
8a	115.4	
8b	104.7	
9	172.7	
1-OCH_3_	55.8	3.80 (s)
6-OCH_3_	56.1	3.88 (s)

**Table 2:** Primer sequences.

Gene	Name	Primers Sequence
GAPDH	Forward Primer Reverse Primer	AGGTCGTGTGAACGGATTTG GGGGTCGTTGATGGCAACA
iNOS	Forward Primer Reverse Primer	GGAGTGACGGCAAACATGACT TCGATGCACAACTGGGTGAAC
TNF-*α*	Forward Primer Reverse Primer	CGCTCTTCTGTCTACTGAACTTCGG GTGGTTTGTGAGTGTGAGGGTCTG
IL-1*β*	Forward Primer Reverse Primer	CACTACAGGCTCCGAGATGAACAAC TGTCGTTGCTTGGTTCTCCTTGTAC
IL-6	Forward Primer Reverse Primer	CTTCTTGGGACTGATGCTGGTGAC TCTGTTGGGAGTGGTATCCTCTGTG
COX-2	Forward Primer Reverse Primer	TCCAACACACTCTATCACTGGC AGAAGCGTTTGCGGTACTCAT

## Data Availability

The original contributions presented in the study are included in the article (and Supplementary Material), further inquiries can be directed to the corresponding authors.

## Supplementary Materials

The following supporting information can be downloaded at: https://www.mdpi.com/article/10.3390/molecules29153705/s1.

## References

[1] Duan Y.L., Deng Y.F., Bu P.F., Guo Y., Shi Z.Y., Cao Y.Y., Zhang Y.T., Hu H., Qi Z.X., Hu C.X. (2021). Discovery of bioactive polycyclic polyprenylated acylphloroglucinols from *Hypericum wilsonii*. Bioorgan. Chem..

[2] Editorial Board of Zhong Hua Ben Cao, State Administration of Traditional Chinese Medicine (1999). Zhong Hua Ben Cao (China Herbal).

[3] Editorial Committee of Flora of China, Chinese Academy of Sciences (1990). *Hypericum* L.. Flora of China.

[4] Editorial Committee of Flora of China, Chinese Academy of Sciences (1990). Hypericum beanii N. Robson. Flora of China.

[5] Yunnan Institute of Materia Medica (2003). Yunnan Natural Medicine Atlas.

[6] Chen X.Q., Li Y., Li K.Z., Peng L.Y., He J., Wang K., Pan Z.H., Cheng X., Li M.M., Zhao Q.S. (2011). Spirocyclic acylphloroglucinol derivatives from *Hypericum beanii*. Chem. Pharm. Bull..

[7] Suo X.Y., Liu X.Y., Liu X.W., Li X.X., Zhu T.T., Ji T.F., Liu B. (2022). Four new polyprenylated acylphloroglucinol derivatives from *Hypericum beanii*. J. Asian Nat. Prod. Res..

[8] Xu W.J., Tang P.F., Lu W.J., Zhang Y.Q., Wang X.B., Zhang H., Luo J., Kong L.Y. (2019). Hyperberins A and B, type B polycyclic polyprenylated acylphloroglucinols with bicyclo[5.3.1]hendecane core from *Hypericum beanii*. Org. Lett..

[9] Libby P. (2007). Inflammatory mechanisms: The molecular basis of inflammation and disease. Nutr. Rev..

[10] Ahn C.B., Jung W.K., Park S.J., Kim Y.T., Kim W.S., Je J.Y. (2016). Gallic Acid-g-Chitosan Modulates Inflammatory Responses in LPS-Stimulated RAW264.7 Cells Via NF-*κ*B, AP-1, and MAPK Pathways. Inflammation.

[11] Kwon D.H., Cha H.J., Choi E.O., Leem S.H., Kim G.Y., Moon S.K., Chang Y.C., Yun S.J., Hwang H.J., Kim B.W. (2018). Schisandrin A suppresses lipopolysaccharide-induced inflammation and oxidative stress in RAW 264.7 macrophages by suppressing the NF-*κ*B, MAPKs and PI3K/Akt pathways and activating Nrf2/HO-1 signaling. Int. J. Mol. Med..

[12] Taketo M.M. (1998). Cyclooxygenase-2 inhibitors in tumorigenesis (part I). J. Natl. Cancer Inst..

[13] Feng Z.L., Lu X.Q., Gan L.S., Zhang Q.W., Lin L.G. (2020). Xanthones, a promising anti-inflammatory scaffold: Structure, activity, and drug likeness analysis. Molecules.

[14] Gnerre C., Thull U., Gaillard P., Carrupt P.A., Testa B., Fernandes E., Silva F., Pinto M., Pinto M.I., Wolfender J.L. (2001). Natural and synthetic xanthones as monoamine oxidase inhibitors: Biologicalassay and 3D-QSAR. Helv. Chim. Acta.

[15] Habib A.M., Reddy K.S., McCloud T.G., Chang C.J., Cassady J.M. (1987). New xanthones from *Psorospermum febrifugum*. J. Nat. Prod..

[16] Dharmaratne H.R., Napagoda M.T., Tennakoon S.B. (2009). Xanthones from roots of *Calophyllum thwaitesii* and their bioactivity. Nat. Prod. Res..

[17] Cardona M.L., Pedro J.R., Seoane E., Vidal R. (1985). Xanthone constituents of *Hypericum canariensis*. J. Nat. Prod..

[18] Frédérich M., Kikuchi H., Tane P., Tchinda A.T., Lonfouo A.H.N., Kowa T.K., Wabo H.K., Oshima Y. (2012). Phenolic Compounds and terpenoids from *Hypericum lanceolatum*. Res. Nat. Prod..

[19] Lin C.N., Chung M.I., Liou S.J., Lee T.H., Wang J.P. (1996). Synthesis and anti-inflammatory effects of xanthone derivatives. J. Pharm. Pharmacol..

[20] Wang Y.C., Zhong F.F., Zhao Y.H., Yang G.Z., Chen Y. (2008). Study on the antioxidant constituents from the barks of *Garcinia xanthochymus*. Nat. Prod. Res. Dev..

[21] Tala M.F., Talontsi F.M., Zeng G.Z., Wabo H.K., Tan N.H., Spiteller M., Tane P. (2015). Antimicrobial and cytotoxic constituents from native Cameroonian medicinal plant *Hypericum riparium*. Fitoterapia.

[22] Poobrasert O., Constant H.L., Beecher C.W., Farnsworth N.R., Kinghorn A.D., Pezzuto J.M., Cordell G.A., Santisuk T., Reutrakul V. (1998). Xanthones from the twigs of *Mammea siamensis*. Phytochemistry.

[23] Li Y.P., Huang S.T. (2020). Xanthones from *Swertia nervosa* and their inhibitory effects on nitric oxide production. Chem. Nat. Compd..

[24] Zhang Y., Shi N.F., Xie Z., Zhao Y.M., Liang C.H., Deng Y.Y., Wang R., Liu Y.P., Fu Y.H. (2023). Chemical constituents from stems and leaves of *Cratoxylum cochinchinense* and their inhibitory effects on proliferation of synoviocytes in vitro. China J. Chin. Mater. Medica.

[25] Marston A., Hamburger M., Sordat-Diserens I., Msonthi J.D., Hostettmann K. (1993). Xanthones from *Polygala nyikensis*. Phytochemistry.

[26] Deng J.T., Hao J., Ma Y.R., Zhou T.X., Huang H.Q. (2021). Study on chemical components of *Hypericum wilsonii* N. Robson. J. Yunnan Univ. Nat. Sci. Ed..

[27] Cardona M.L., Fernández M.I., Pedro J.R., Seoane E., Vidal R. (2004). Additional new xanthones and xanthonolignoids from *Hypericum canariensis*. J. Nat. Prod..

[28] Zhang Z.Z., ElSohly H.N., Jacob M.R., Pasco D.S., Walker L.A., Clark A.M. (2002). Natural products inhibiting *Candida albicans* secreted aspartic proteases from *Tovomita krukovii*. Planta. Med..

[29] Li Y.Z., Li Z.L., Hua H.M., Li Z.G., Liu M.S. (2007). Studies on flavonoids from stems and leaves of *Calophyllum inophyllum*. China J. Chin. Mater. Medica.

[30] Jiang D.J., Hu G.Y., Jiang J.L., Xiang H.L., Deng H.W., Li Y.J. (2003). Relationship between protective effect of xanthone on endothelial cells and endogenous nitric oxide synthase inhibitors. Bioorganic Med. Chem..

[31] Gonda R., Takeda T., Akiyama T. (2001). Studies on the constituents of Anaxagorea luzonensis A. GRAY II. Nat. Med..

[32] Ghosal S., Chaudhuri R.K., Nath A. (1973). Chemical constituents of gentianaceae IV: New xanthones of *Canscora decussata*. J. Pharm. Sci..

[33] Wolfender J.L., Hamburger M., Msonthi J.D., Hostettmann K. (1991). Xanthones from *Chironia krebsii*. Phytochemistry.

[34] Wang H., Ye G., Ma C.H., Tang Y.H., Fan M.S., Li Z.X., Huang C.G. (2007). Identification and determination of four metabolites of mangiferin in rat urine. J. Pharm. Biomed. Anal..

[35] Ngouela S., Zelefack F., Lenta B.N., Ngouamegne E.T., Tchamo D.N., Tsamo E., Connolly J.D. (2005). Xanthones and other constituents of *Allanblackia monticola* (Guttiferae). Nat. Prod. Res..

[36] Hu L.H., Yip S.C., Sim K.Y. (1999). Xanthones from *Hypericum ascyron*. Phytochemistry.

[37] Kanwar J.R., Kanwar R.K., Burrow H., Baratchi S. (2009). Recent advances on the roles of NO in cancer and chronic inflammatory disorders. Curr. Med. Chem..

[38] Khatua S., Simal-Gandara J., Acharya K. (2022). Understanding immune-modulatory efficacy in vitro. Chem. Biol. Interact..

[39] Ma W., Ren F.C., Yan X.W., Wang X.R., Wu T.N., Li N. (2024). Cytotoxic and anti-inflammatory constituents from roots of *Hypericum beanii* and the antitumor potential under the view of cancer-related inflammation. Fitoterapia.

[40] DeRijk R., Michelson D., Karp B., Petrides J., Galliven E., Deuster P., Paciotti G., Gold P.W., Sternberg E.M. (1997). Exercise and circadian rhythm-induced variations in plasma cortisol differentially regulate interleukin-1*β* (IL-1*β*), IL-6, and tumor necrosis factor-*α* (TNF-*α*) production in humans: High sensitivity of TNF-*α* and resistance of IL-6. J. Clin. Endocrinol. Metab..

[41] Cho B.O., Ryu H.W., So Y., Chang W.L., Chang H.J., Hong S.Y., Yong W.J., Park J.C., Jeong I.Y. (2014). Anti-inflammatory effect of mangostenone F in lipopolysaccharide-stimulated RAW264.7 macrophages by suppressing NF-κB and MAPK activation. Biomol. Ther..

[42] Li D., Liu Q., Sun W., Chen X., Wang Y., Sun Y., Lin L. (2018). 1, 3, 6, 7-Tetrahydroxy-8-prenylxanthone ameliorates inflammatory responses resulting from the paracrine interaction of adipocytes and macrophages. Br. J. Pharmacol..

[43] Jeong G.S., Lee D.S., Kim Y.C. (2009). Cudratricusxanthone A from *Cudrania tricuspidata* suppresses pro-inflammatory mediators through expression of anti-inflammatory heme oxygenase-1 in RAW264.7 macrophages. Int. Immunopharmacol..

[44] Toshihiro N., Takahashi-Yanaga F., Arioka M., Mori Y., Sasaguri T. (2016). Inhibition of GSK-3 reduces prostaglandin E2 production by decreasing the expression levels of COX-2 and mPGES-1 in monocyte/macrophage lineage cells. Biochem. Pharmacol..

[45] Koopklang K., Choodej S., Hantanong S., Intayot R., Jungsuttiwong S., Insumran Y., Ngamrojanavanich N., Pudhom K. (2024). Anti-inflammatory properties of oxygenated isocoumarins and xanthone from thai mangrove-associated endophytic fungus *Setosphaeria rostrata*. Molecules.

[46] Liu Z.Z., Ma J.C., Deng P., Ren F.C., Li N. (2023). Chemical constituents of *thesium chinense* turcz and their in vitro antioxidant, anti-inflammatory and cytotoxic activities. Molecules.

