# Supplementary material for: Anti-Inflammatory Effect of Xanthones from Hypericum beanii on Macrophage RAW 264.7 Cells through Reduced NO Production and TNF-α, IL-1β, IL-6, and COX-2 Expression
Source: Molecules. 2024 Aug 5;29(15):3705. doi: 10.3390/molecules29153705 (PMC11313822; doi:10.3390/molecules29153705)
Supplement: Supplementary file 1 [file molecules-29-03705-s001.zip › molecules-3101946-supplementary.pdf]

Supplementary Materials

Anti-Inflammatory Effect of Xanthones from *Hypericum beanii* on Macrophage RAW 264.7 Cells through Reduced NO Production and TNF-α, IL-1β, IL-6, and COX-2 Expression

Wei Ma †, Fu-Cai Ren †, Xue-Ru Wang and Ning Li *

School of Pharmacy, Anhui Medical University, No.81 Meishan Road, Shushan District, Hefei 230032, China; mw421553449@sina.com (W.M.); renfucai@ahmu.edu.cn (F.-C.R.); 19855867686@139.com (X.-R.W.)
* Correspondence: 1993500019@ahmu.edu.cn
† These authors contributed equally to this work.

Table of Contents

Figure S1. ^{1}H-NMR, spectrum of compound **1**........................ ....S3

Figure S2. ^{13}C-NMR and ^{13}C DEPT-135 spectra of compound **1**.............................S3

Figure S3. ^{1}H-^{1}H COSY spectrum of compound **1**...S4

Figure S4. HSQC spectrum of compound **1** S4

Figure S5. HMBC spectrum of compound **1** S5

Figure S6. HRESIMS plot of compound **1** S6

Figure S7. IR spectrum of compound **1** S7

Figure S8. UV spectrum of compound **1**... ....S7

1D-NMR spectra and data of compounds **2**–**24**...S8

A survey of natural compounds **2**–**24**..S36

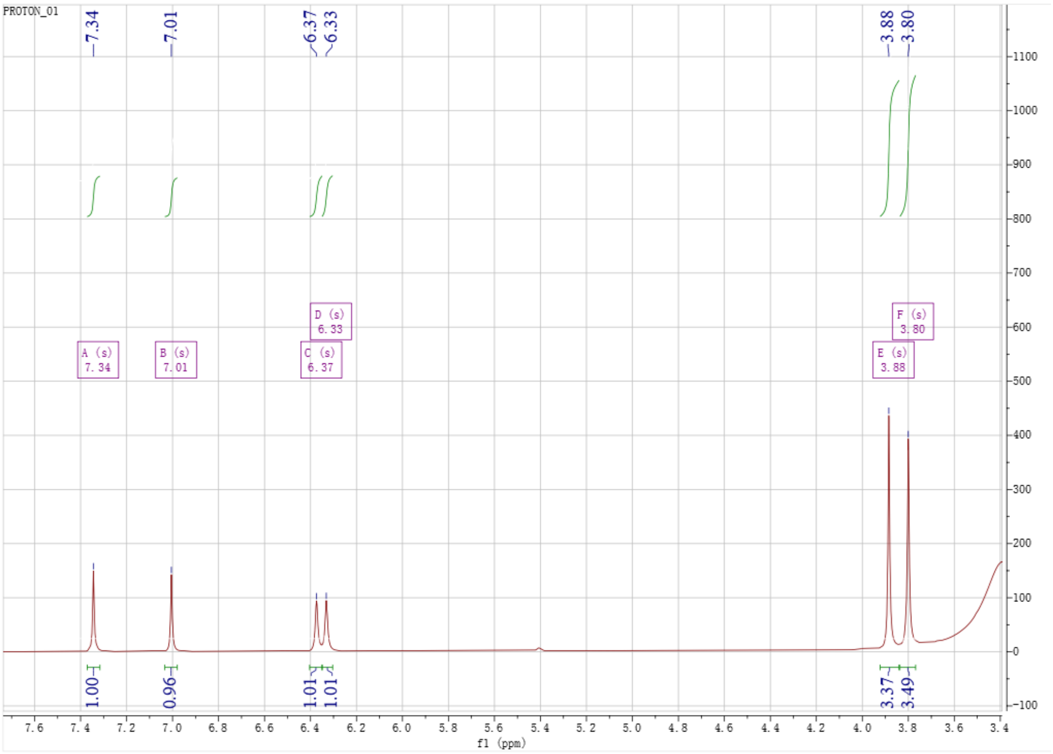

Figure S1. ^{1}H-NMR spectrum of compound **1** (600 MHz, DMSO-d_6)

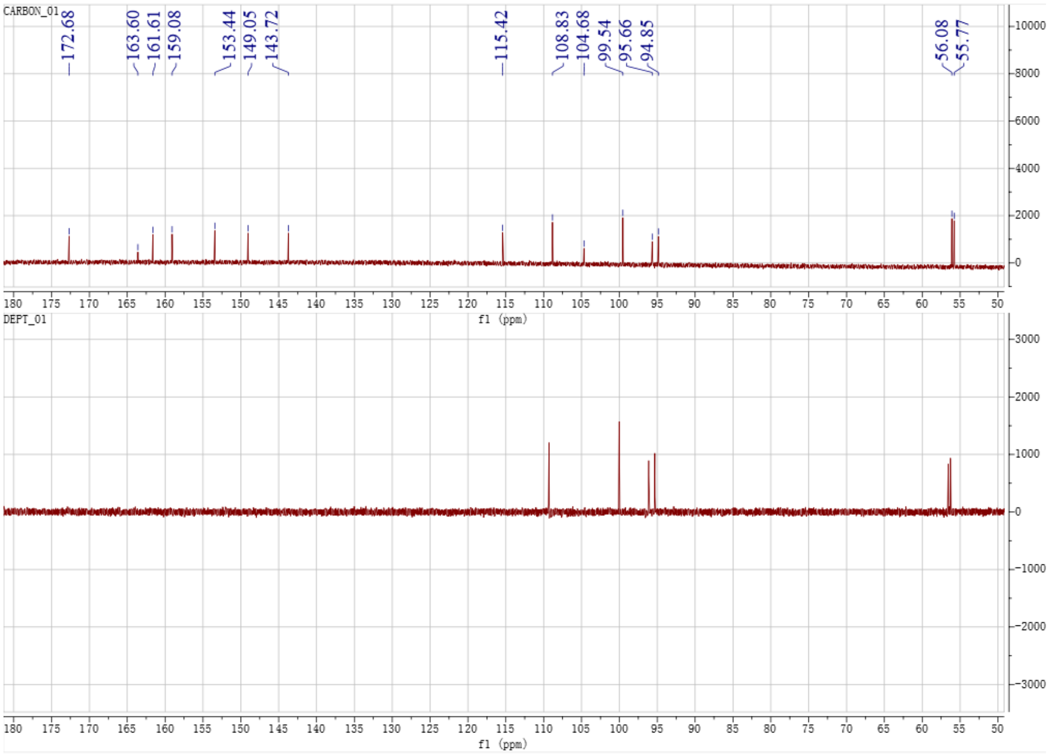

Figure S2. ^{13}C-NMR and ^{13}C DEPT-135 spectra of compound **1** (151 MHz, DMSO-d_6)

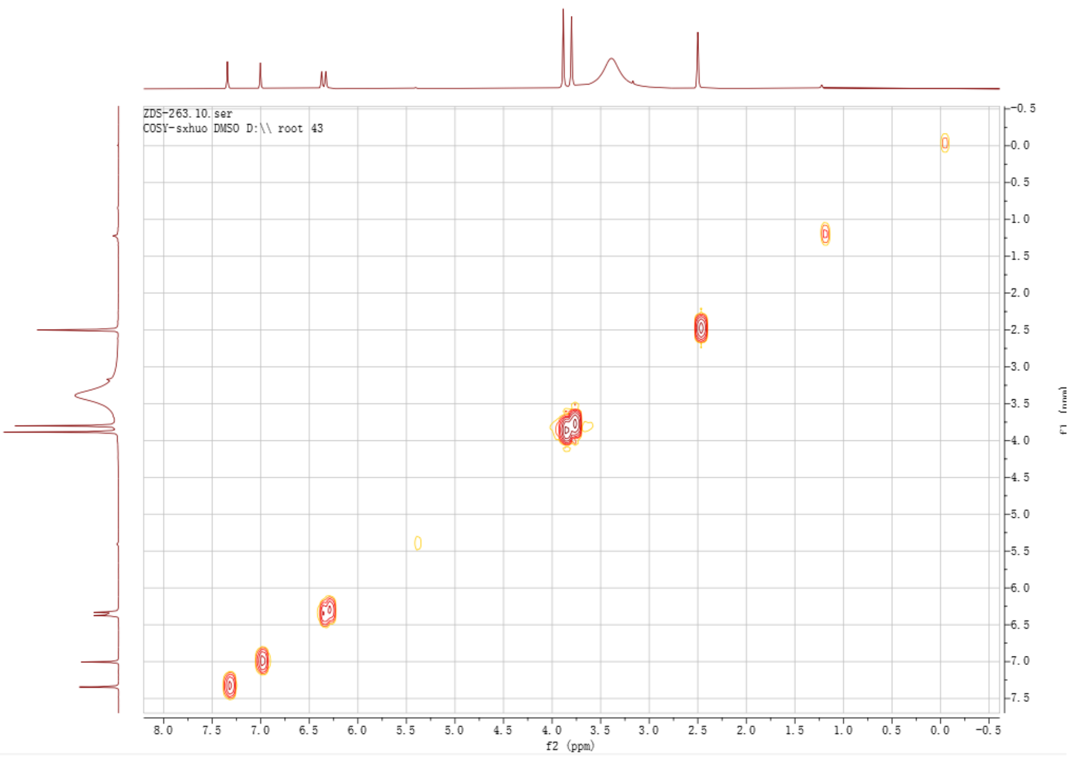

Figure S3. ^{1}H-^{1}H COSY spectrum of compound **1** (600 MHz, DMSO-*d*$_6$)

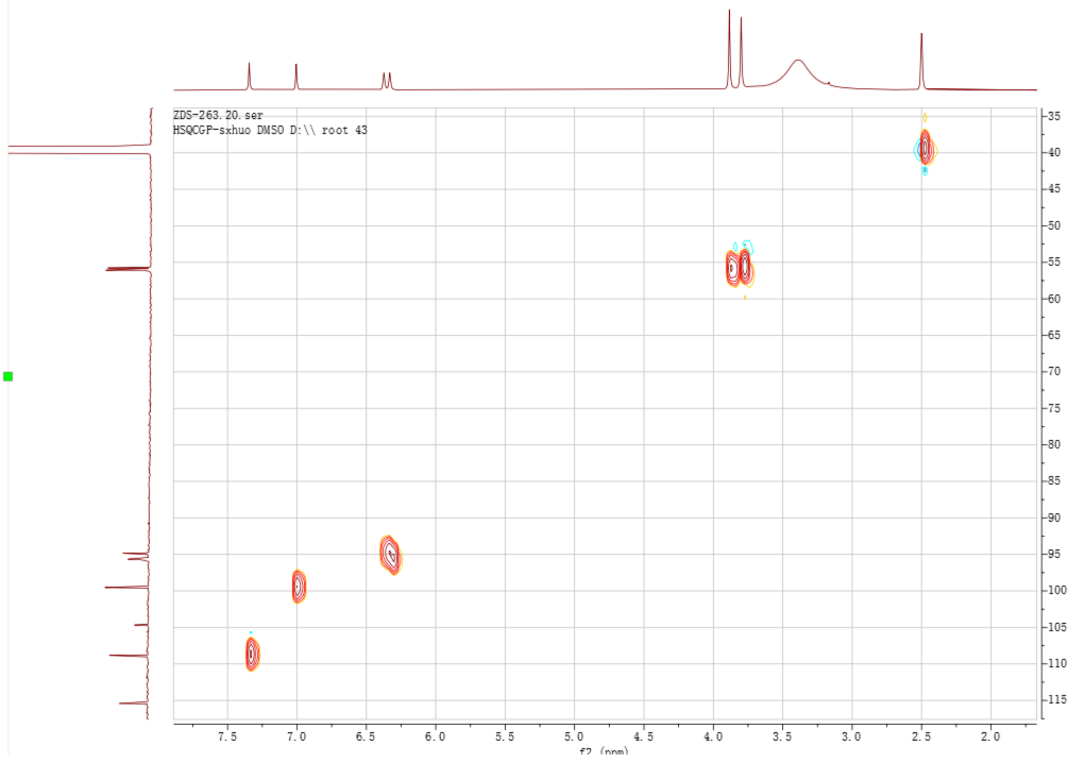

Figure S4. HSQC spectrum of compound **1** (600 MHz, DMSO-*d*$_6$)

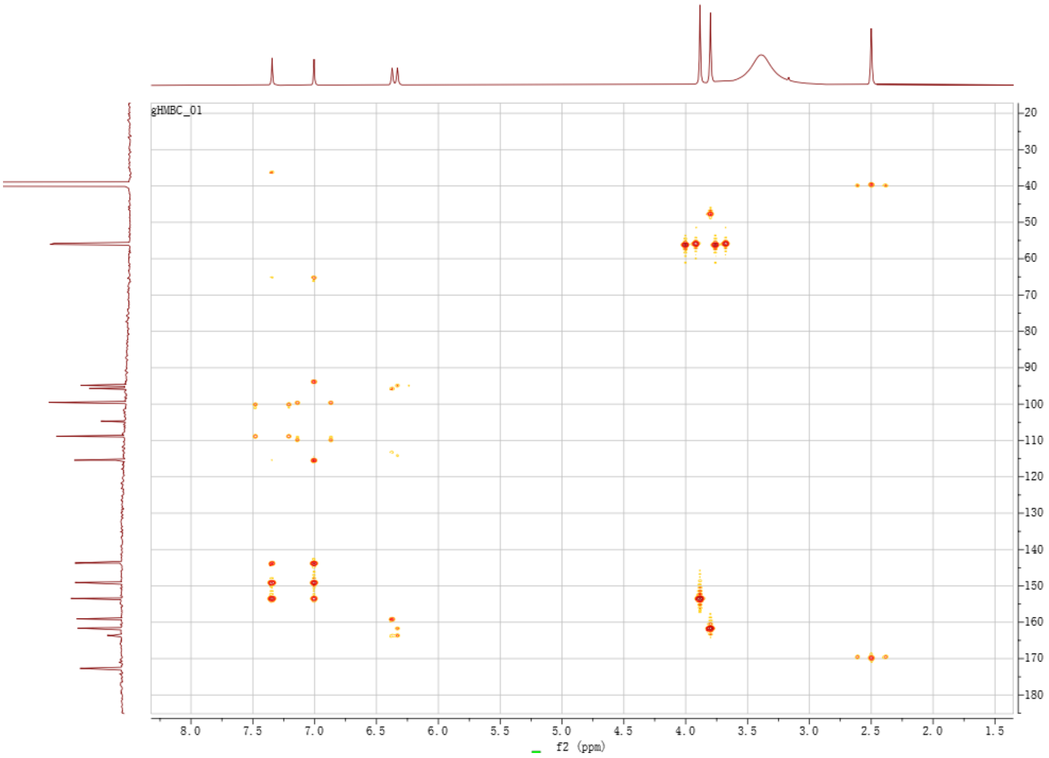

Figure S5. HMBC spectrum of compound **1** (600 MHz, DMSO-d_6)

Qualitative Analysis Report

**Data Filename**	ZDS-263.d	**Sample Name**	ZDS-263
**Sample Type**	Sample	**Position**	P1-F2
**Instrument Name**	Instrument 1	**User Name**	
**Acq Method**	s-.m	**Acquired Time**	2/27/2024 1:55:29 PM
**IRM Calibration Status**	Success	**DA Method**	PCDL.m
**Comment**			

Sample Group **Info.**

Acquisition SW 6200 series TOF/6500 series
Version Q-TOF B.05.01 (B5125.2)

User Spectra

Fragmentor Voltage	Collision Energy	Ionization Mode
135	0	ESI

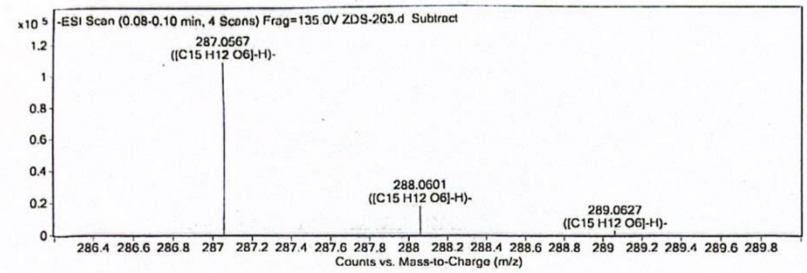

Peak List

m/z	z	Abund	Formula	Ion
89.0245	1	5330.09		
97.9998	1	4527.42		
112.9855	1	6311.79		
257.0458	1	8059.69		
287.0567	1	109191.5	C15 H12 O6	(M-H)-
288.0601	1	18559.99	C15 H12 O6	(M-H)-
323.0334	1	3561.71		
355.0443	1	5619.92		
575.1207	1	18614.2		
576.1248	1	6282.3		

Formula Calculator Element Limits

Element	Min	Max
C	3	60
H	0	120
O	0	10

Formula Calculator Results

Formula	CalculatedMass	CalculatedMz	Mz	Diff. (mDa)	Diff. (ppm)	DBE
C15 H12 O6	288.0634	287.0561	287.0567	-0.60	-2.09	10.0000

--- End Of Report ---

Agilent Technologies Page 1 of 1 Printed at: 4:10 PM on: 2/29/2024

Figure S6. HR-ESI-MS spectrum of compound **1**

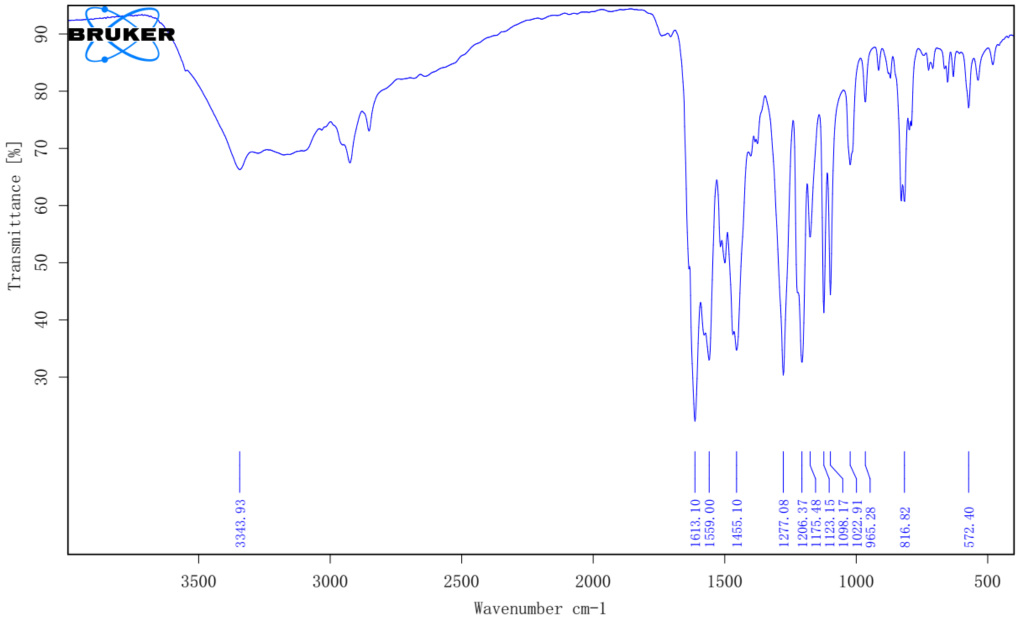

Figure S7. IR spectrum of compound **1**

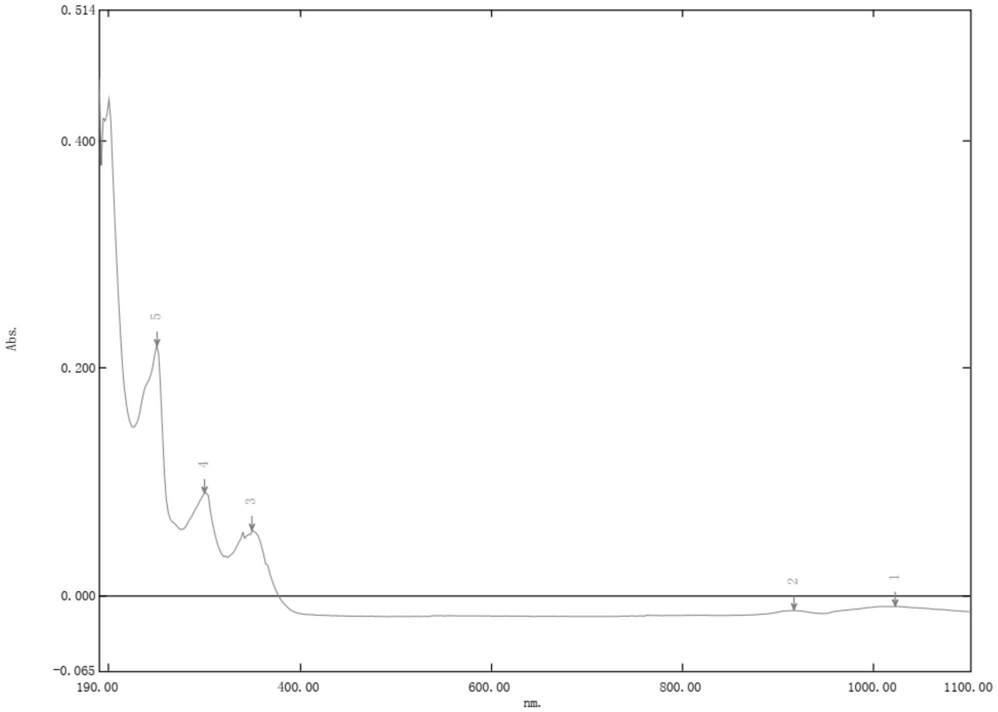

Figure S8. UV spectrum of compound **1**

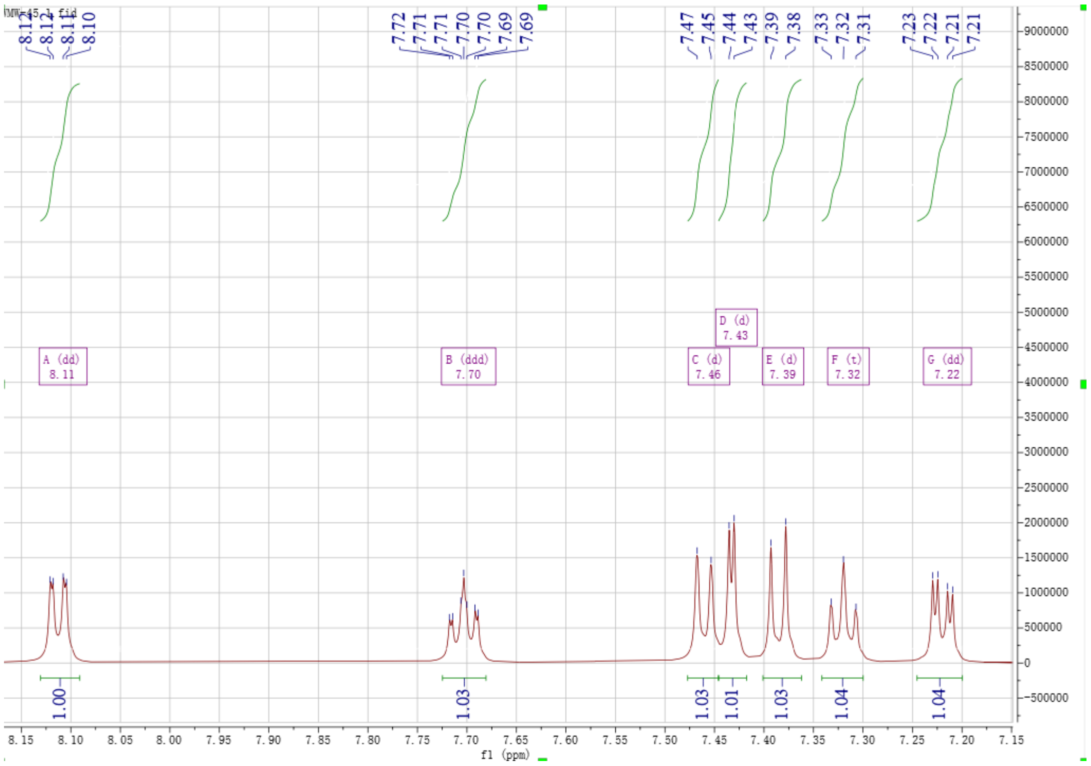

^{1}H-NMR spectrum of compound **2** (600 MHz, DMSO-d_6)

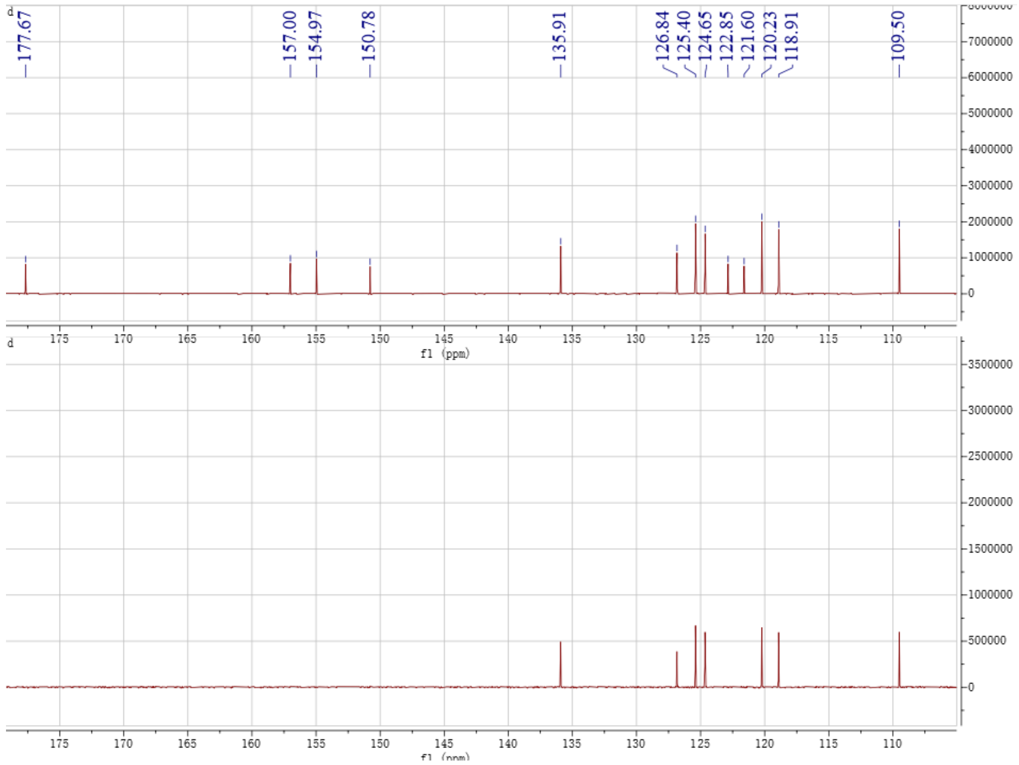

^{13}C-NMR and ^{13}C DEPT-135 spectra of compound **2** (151 MHz, DMSO-d_6)

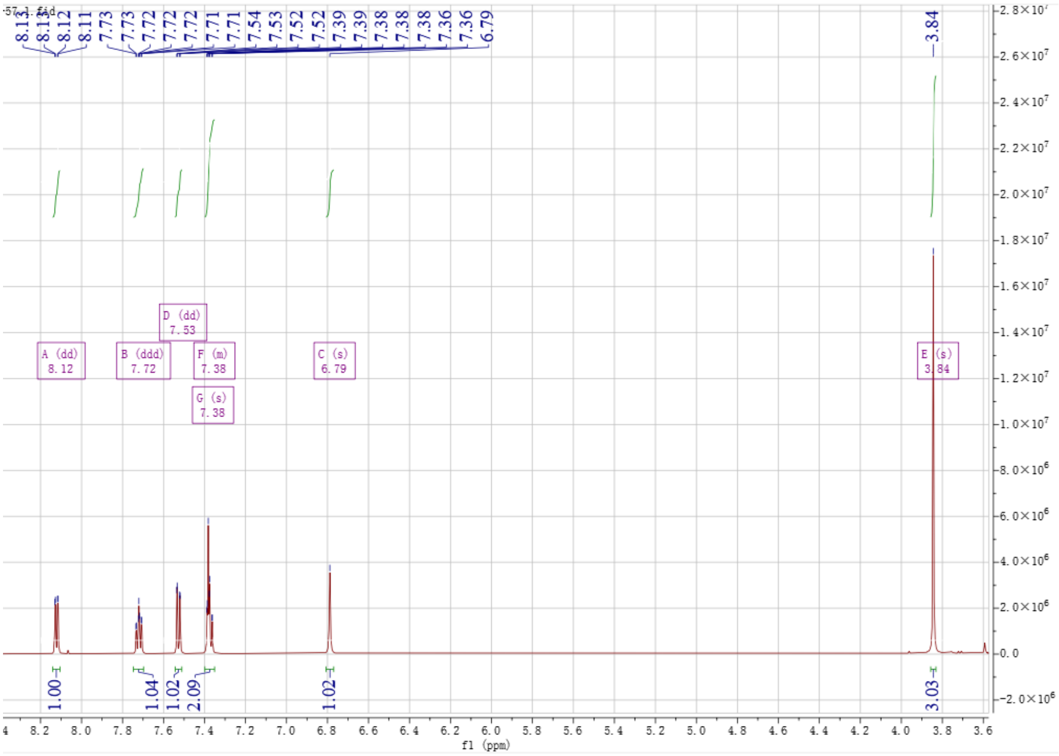

^{1}H-NMR spectrum of compound **3** (600 MHz, DMSO-d_6)

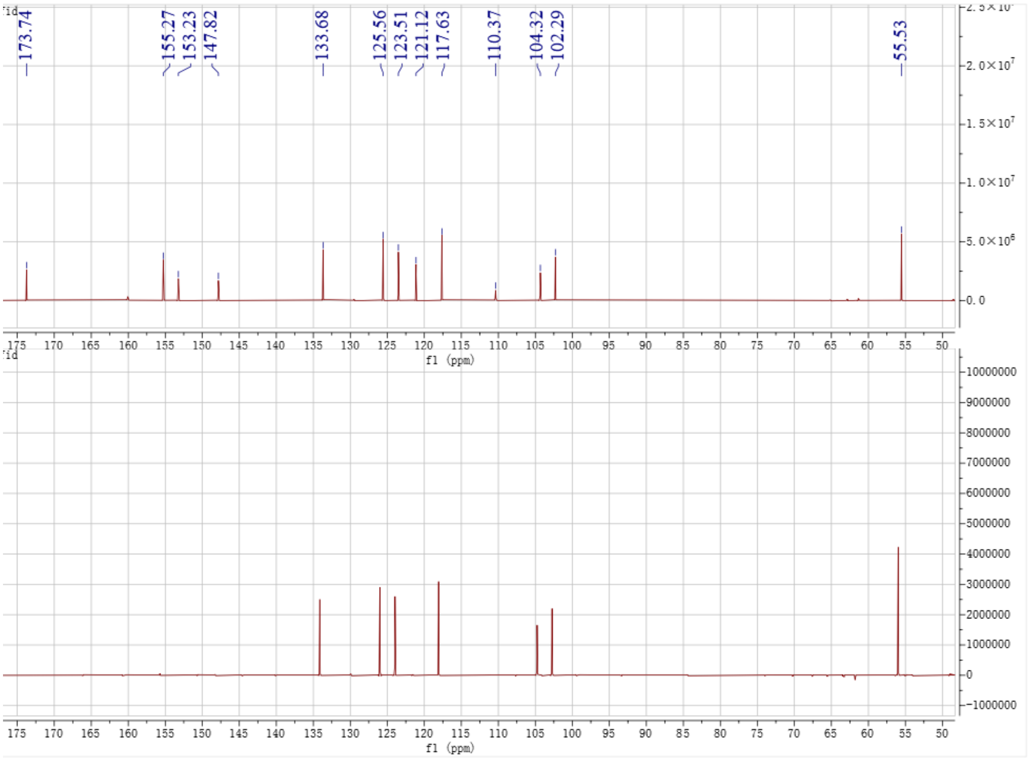

^{13}C-NMR and ^{13}C DEPT-135 spectra of compound **3** (151 MHz, DMSO-d_6)

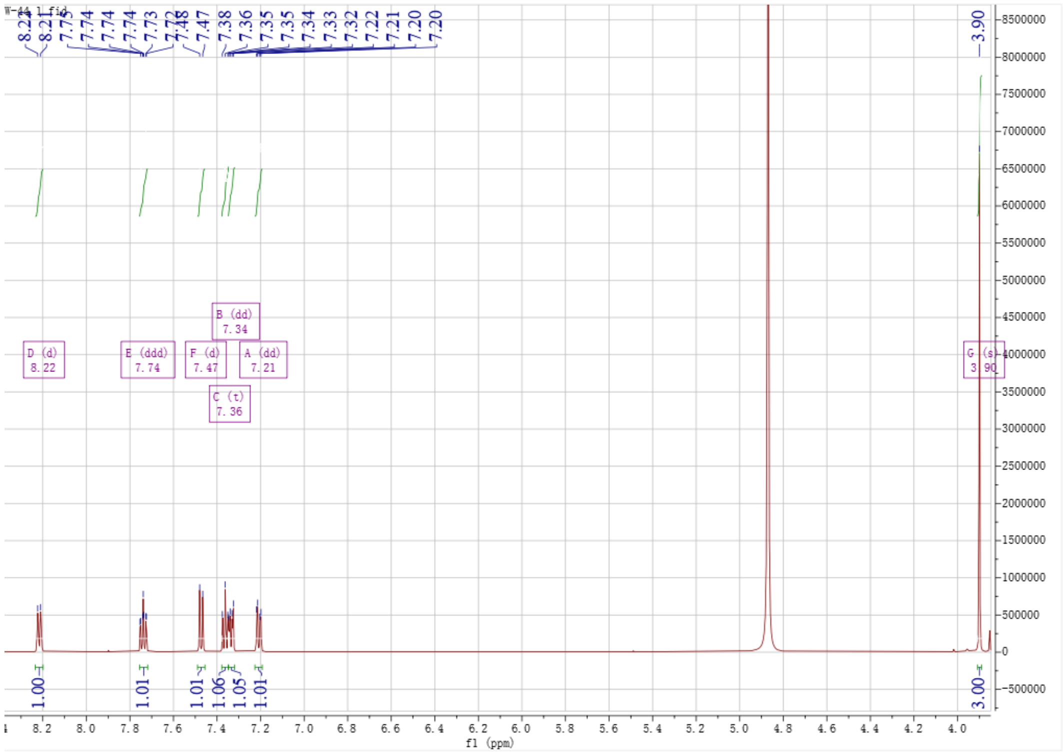

^{1}H-NMR spectrum of compound **4** (600 MHz, CD_3OD)

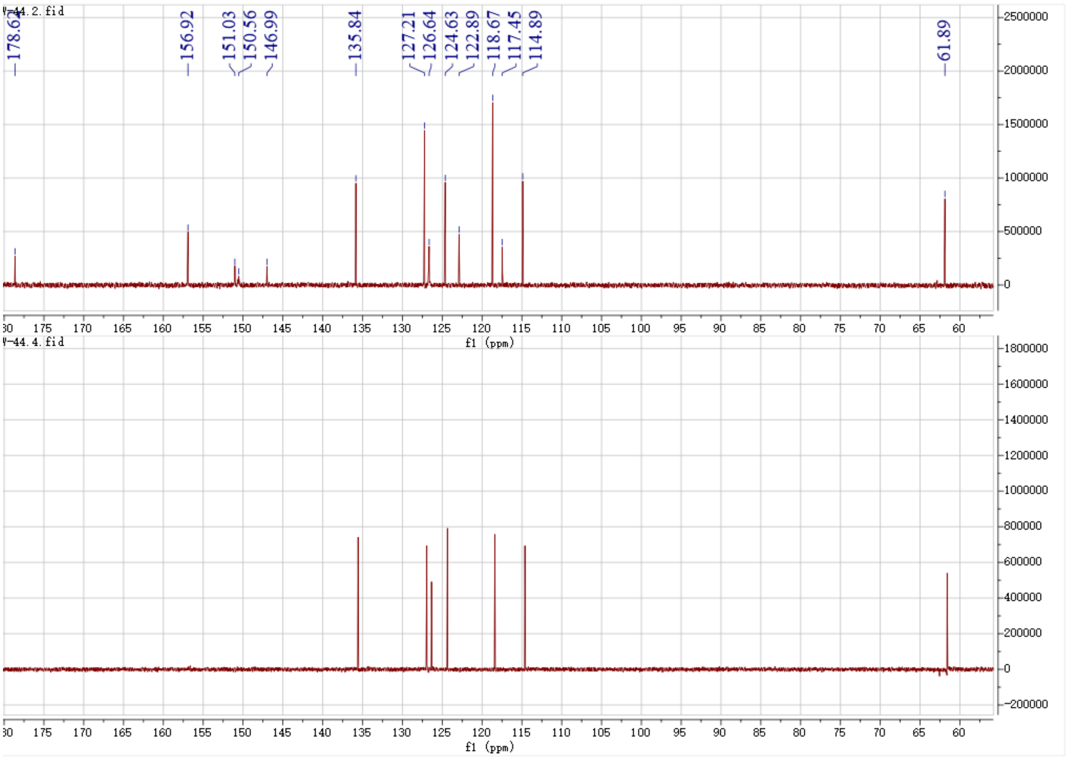

^{13}C-NMR and ^{13}C DEPT-135 spectra of compound **4** (151 MHz, CD_3OD)

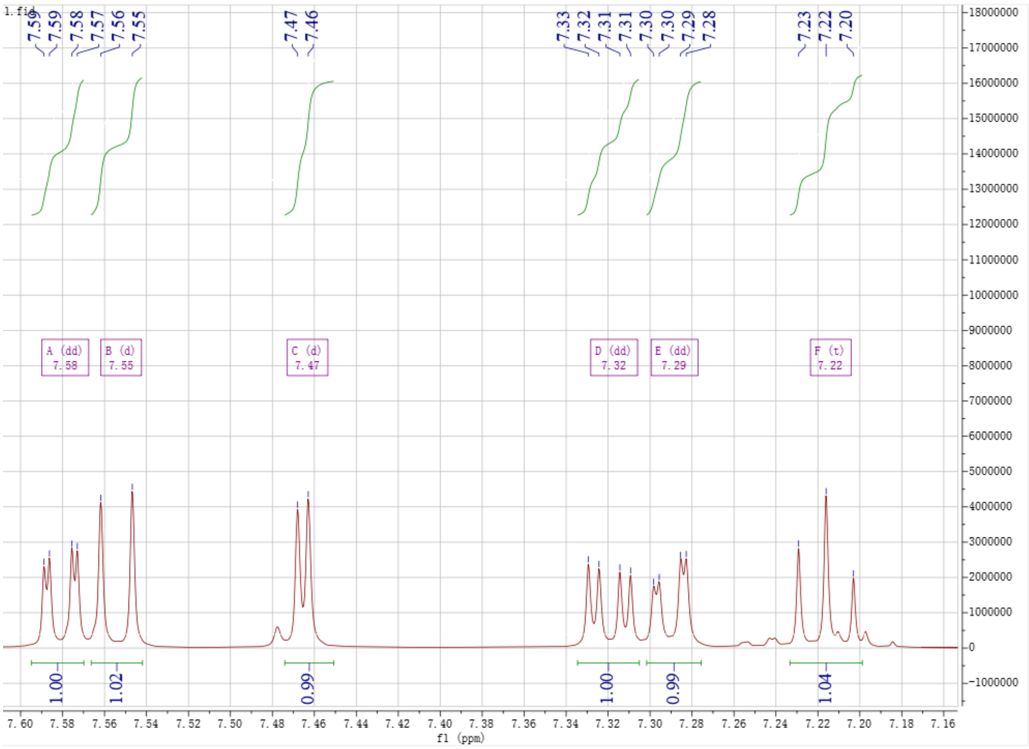

^{1}H-NMR spectrum of compound **5** (600 MHz, DMSO-d_6)

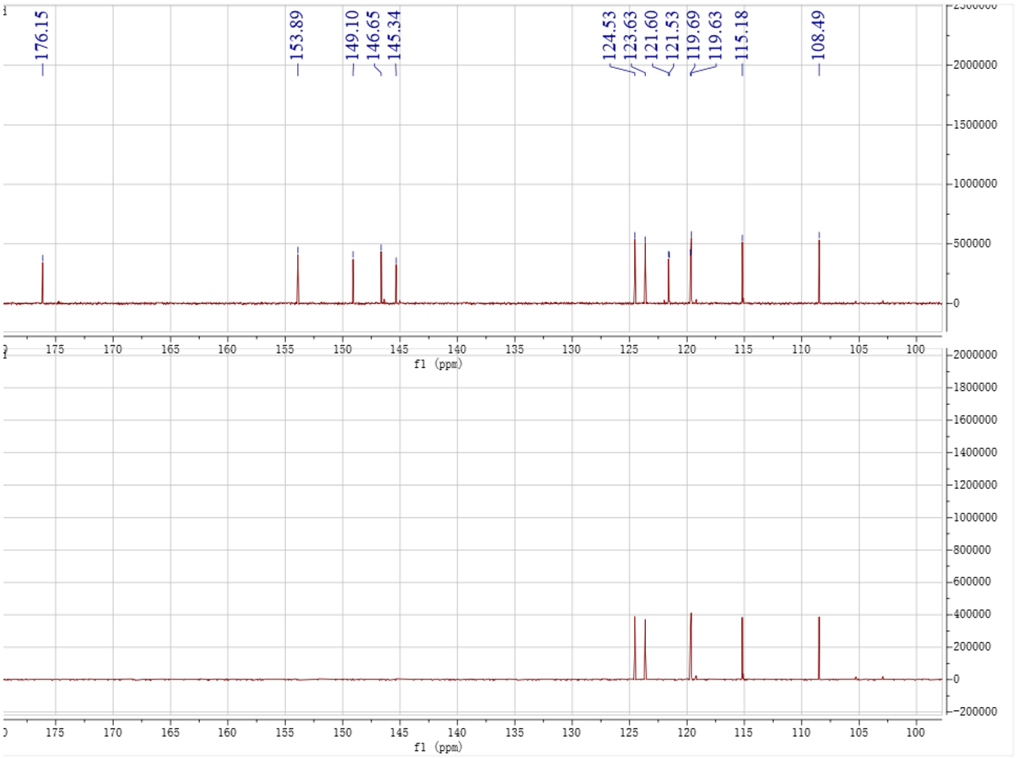

^{13}C-NMR and ^{13}C DEPT-135 spectra of compound **5** (151 MHz, DMSO-d_6)

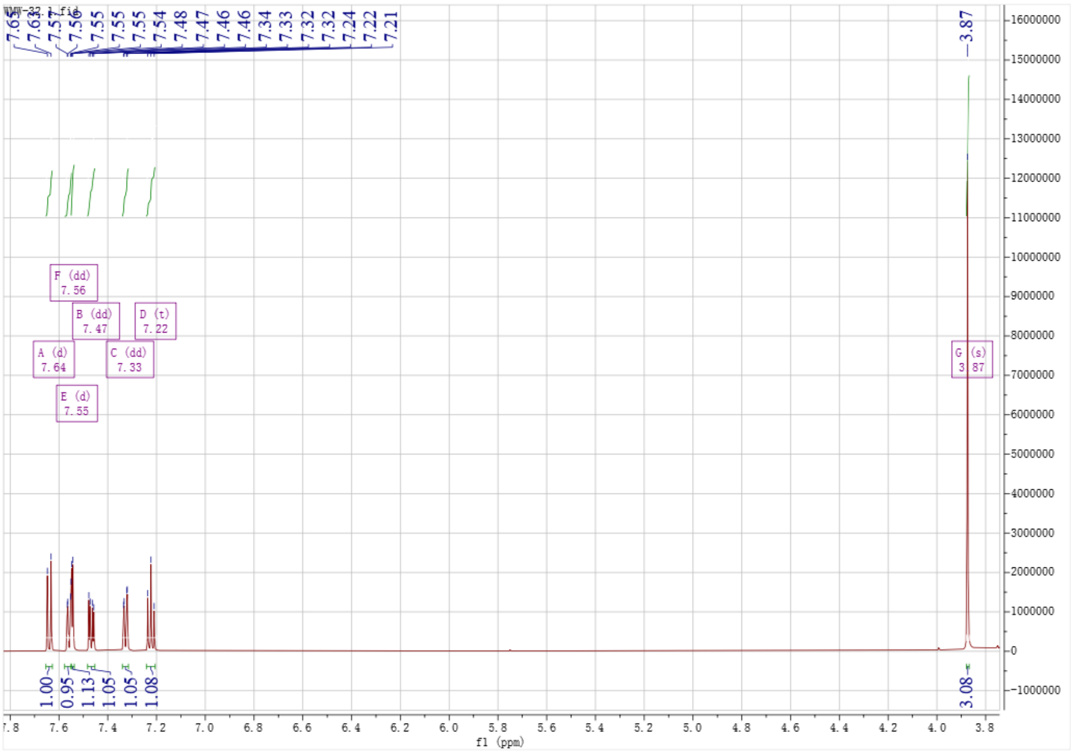

^{1}H-NMR spectrum of compound **6** (600 MHz, DMSO-d_6)

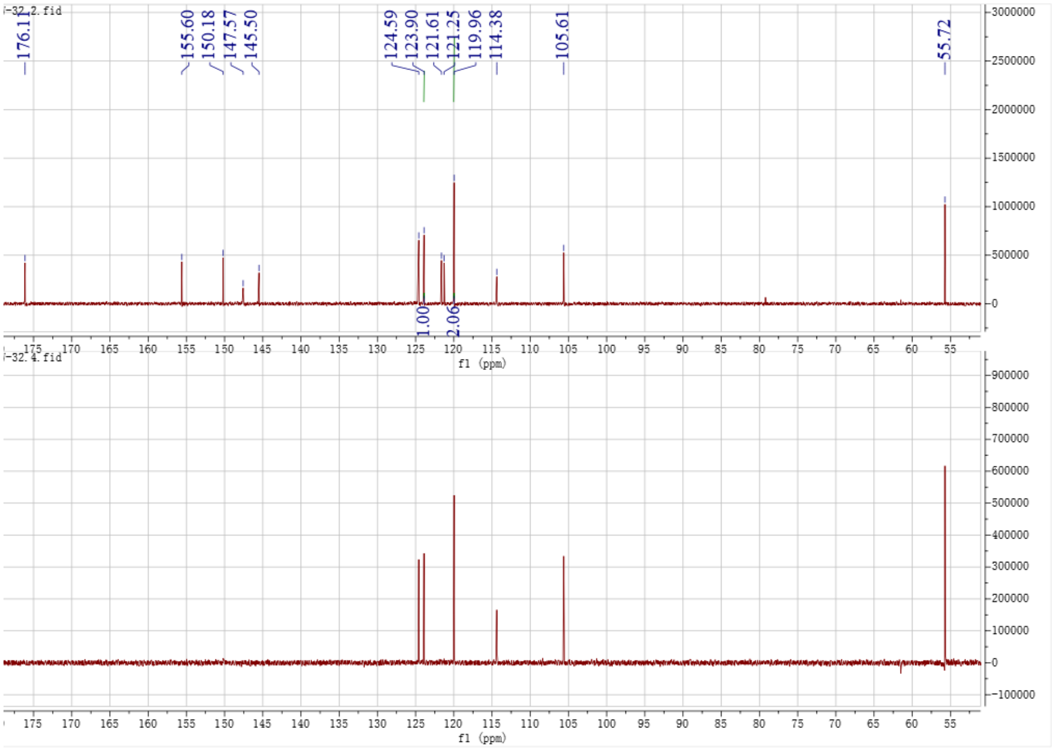

^{13}C-NMR and ^{13}C DEPT-135 spectra of compound **6** (151 MHz, DMSO-d_6)

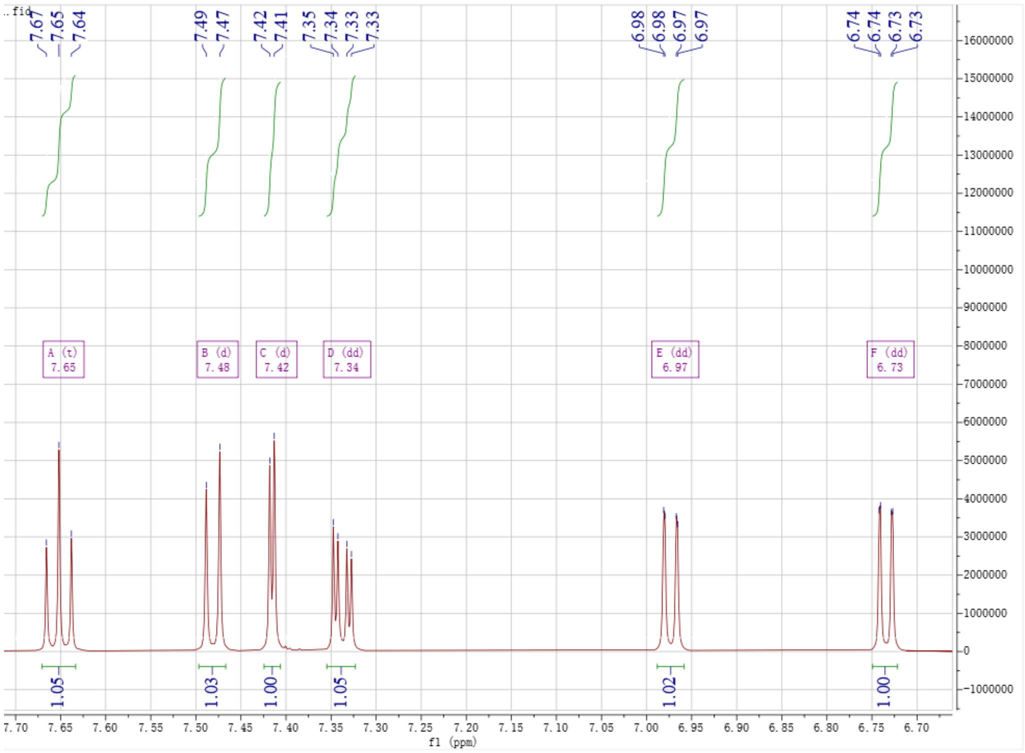

^{1}H-NMR spectrum of compound **7** (600 MHz, DMSO-d_6)

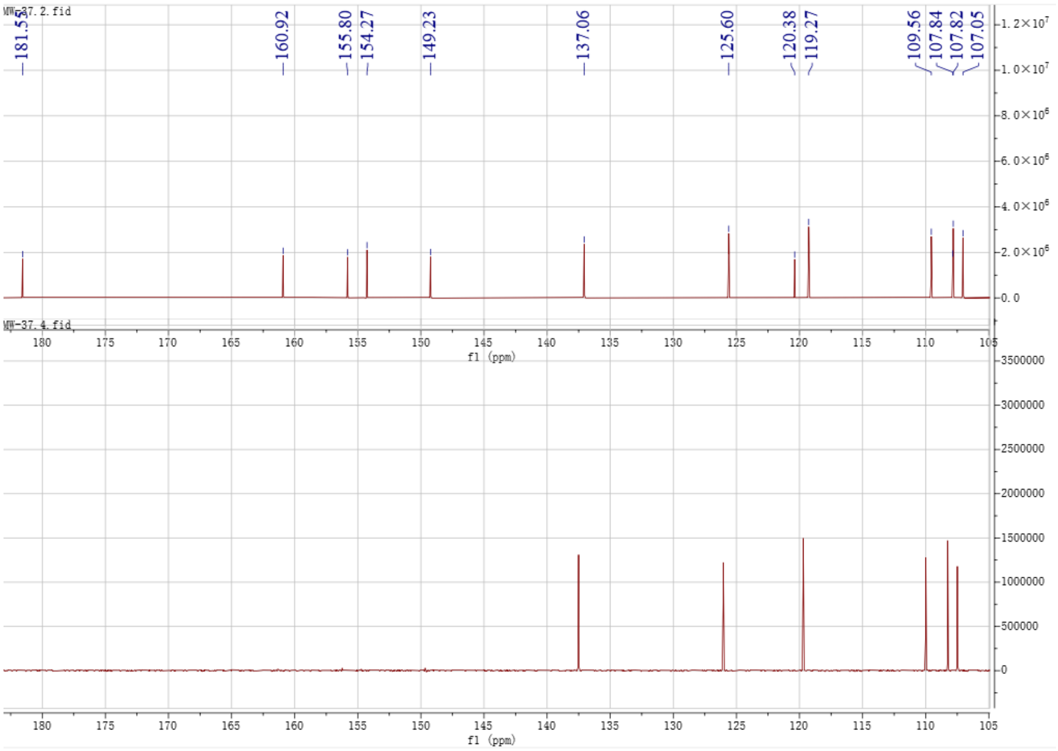

^{13}C-NMR and ^{13}C DEPT-135 spectra of compound **7** (151 MHz, DMSO-d_6)

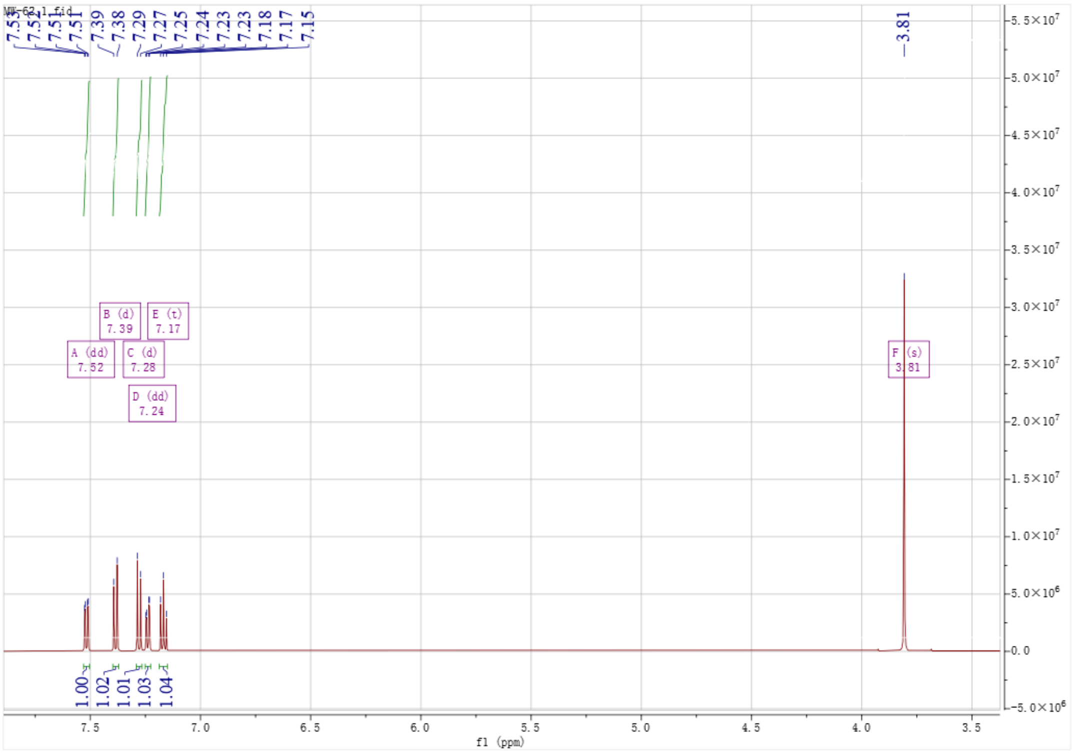

^{1}H-NMR spectrum of compound **8** (600 MHz, DMSO-*d*$_6$)

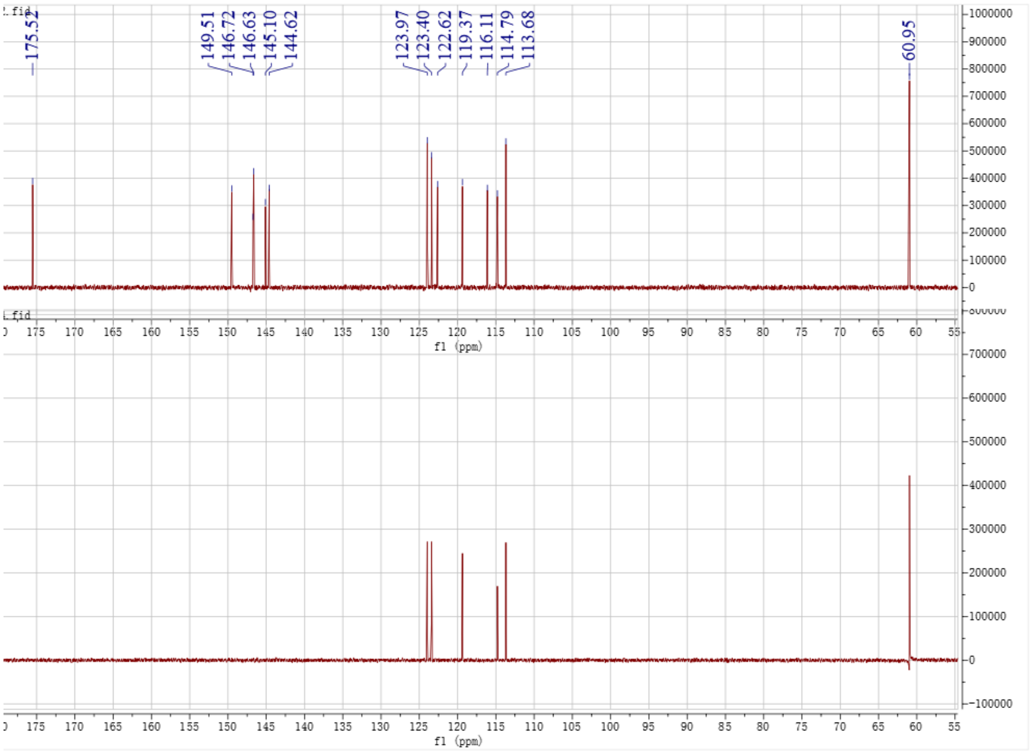

^{13}C-NMR and ^{13}C DEPT-135 spectra of compound **8** (151 MHz, DMSO-*d*$_6$)

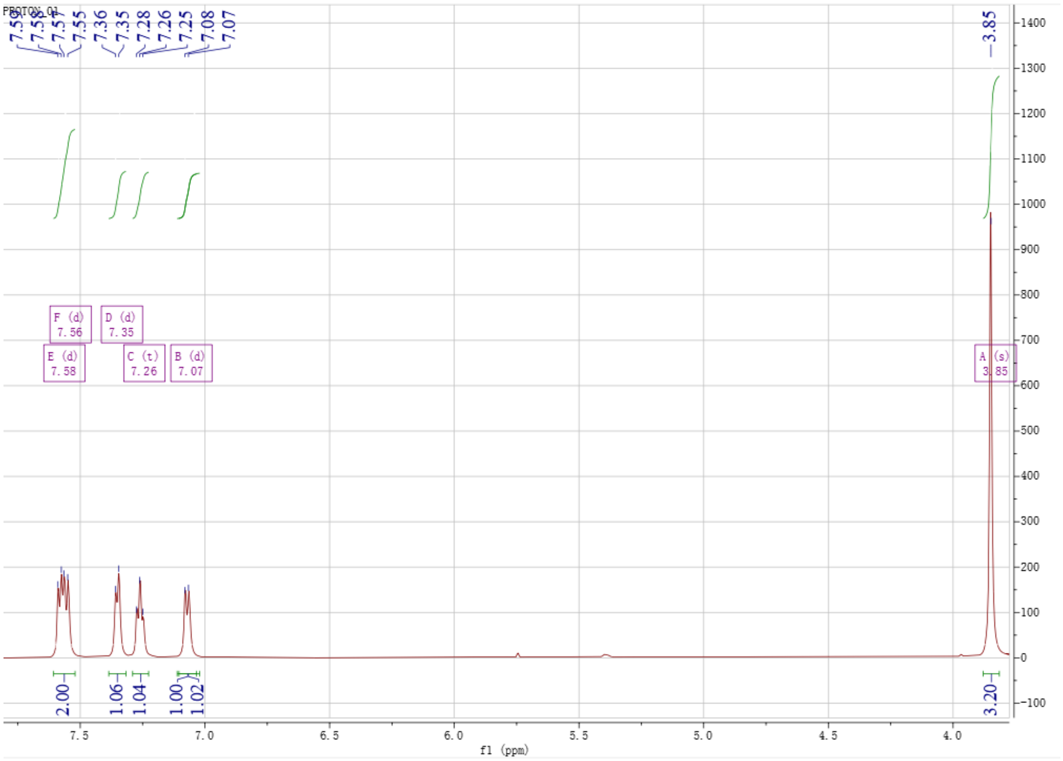

^{1}H-NMR spectrum of compound **9** (600 MHz, DMSO-d_6)

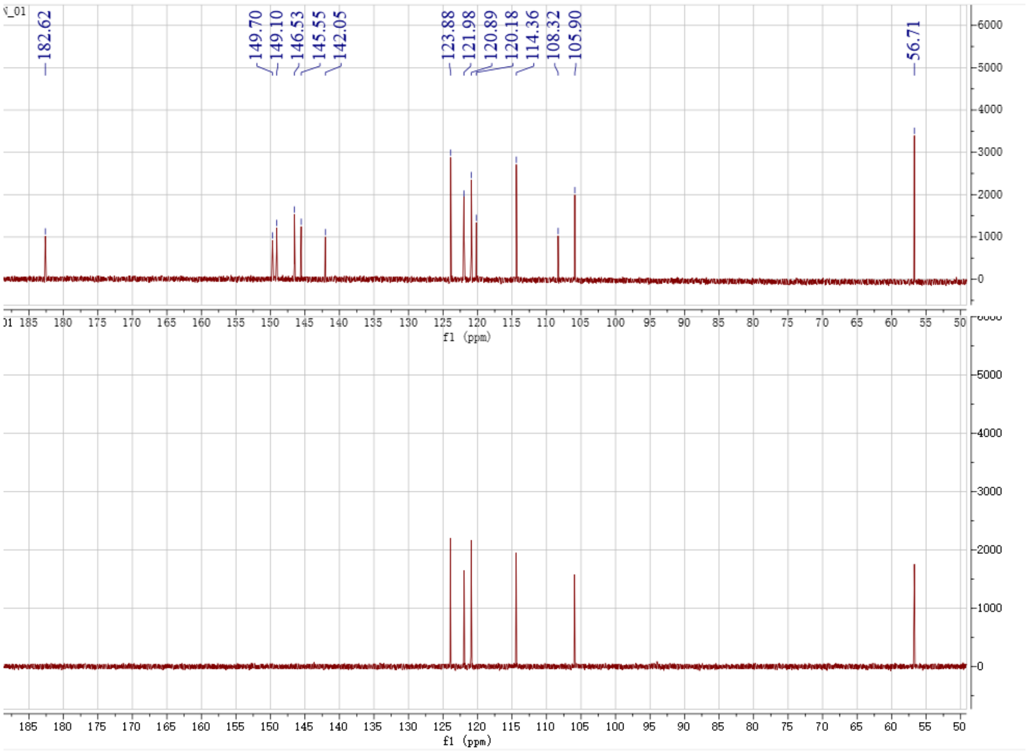

^{13}C-NMR and ^{13}C DEPT-135 spectra of compound **9** (151 MHz, DMSO-d_6)

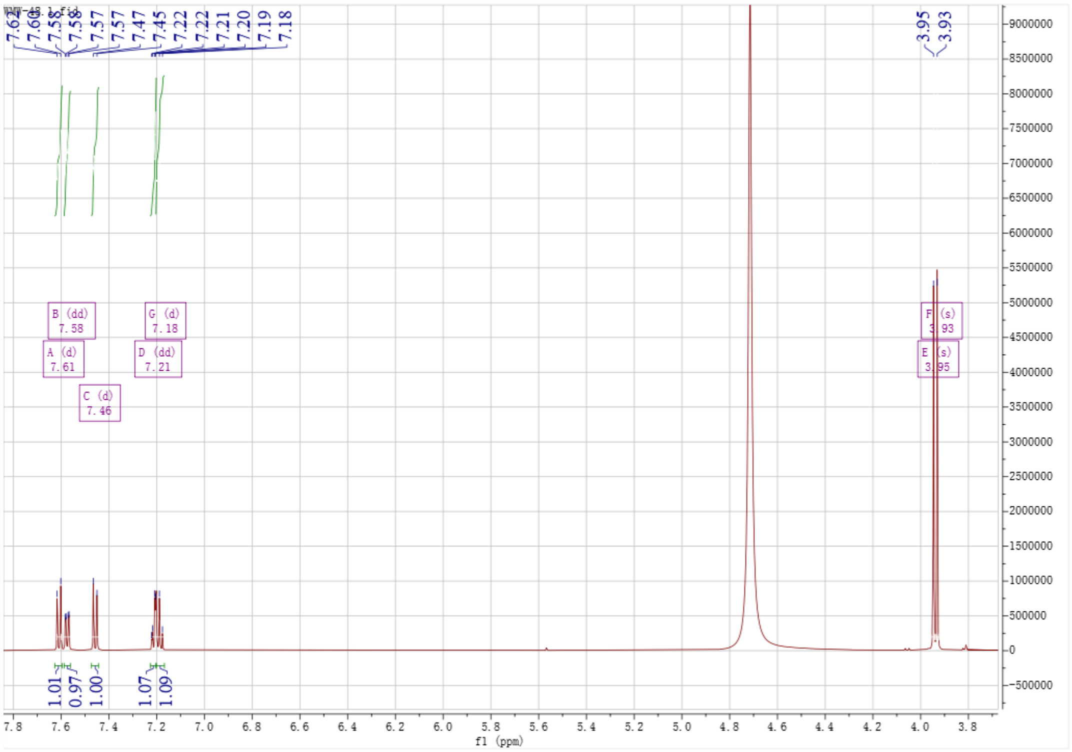

^{1}H-NMR spectrum of compound **10** (600 MHz, CD_3OD)

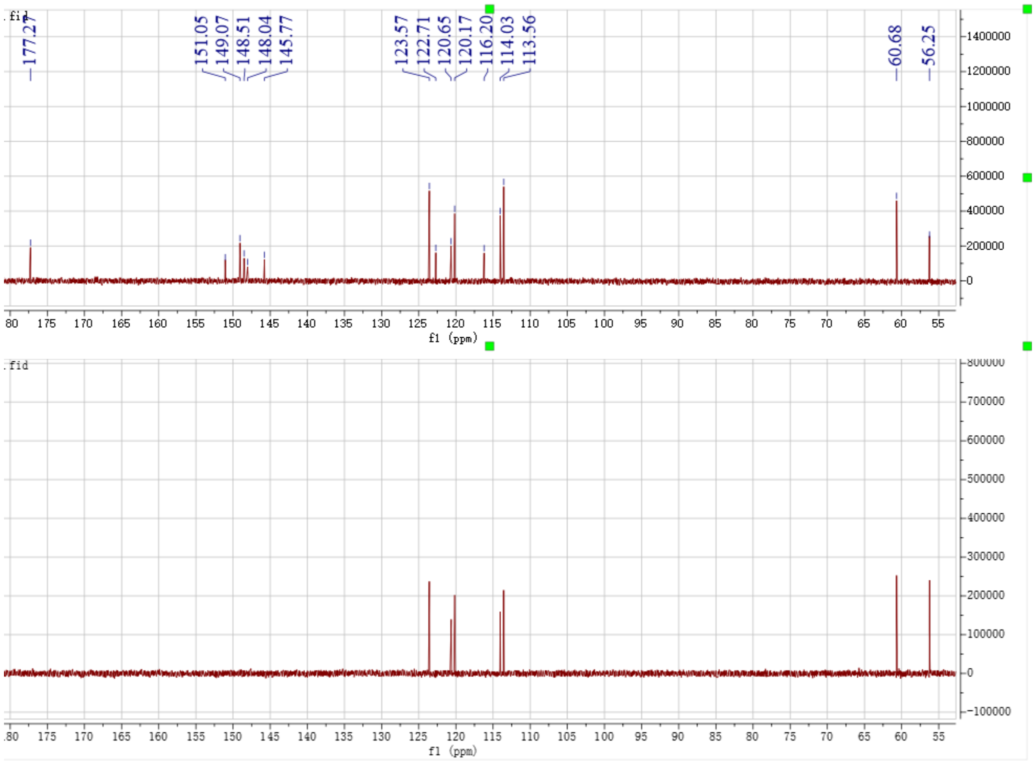

^{13}C-NMR and ^{13}C DEPT-135 spectra of compound **10** (151 MHz, CD_3OD)

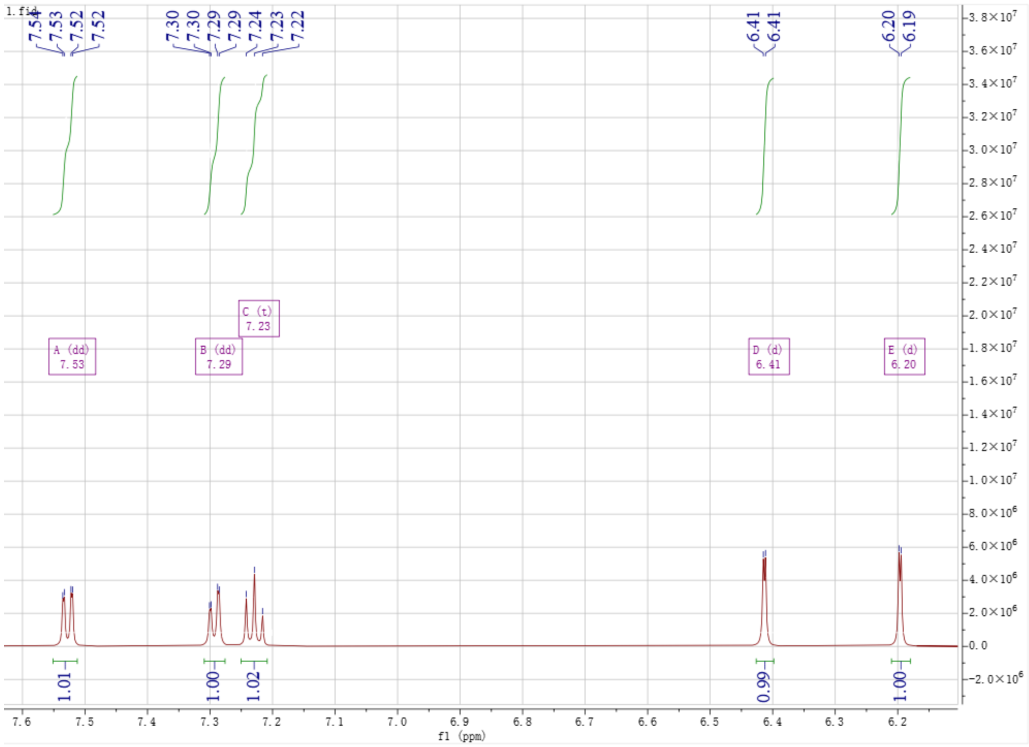

^{1}H-NMR spectrum of compound **11** (600 MHz, DMSO-d_6)

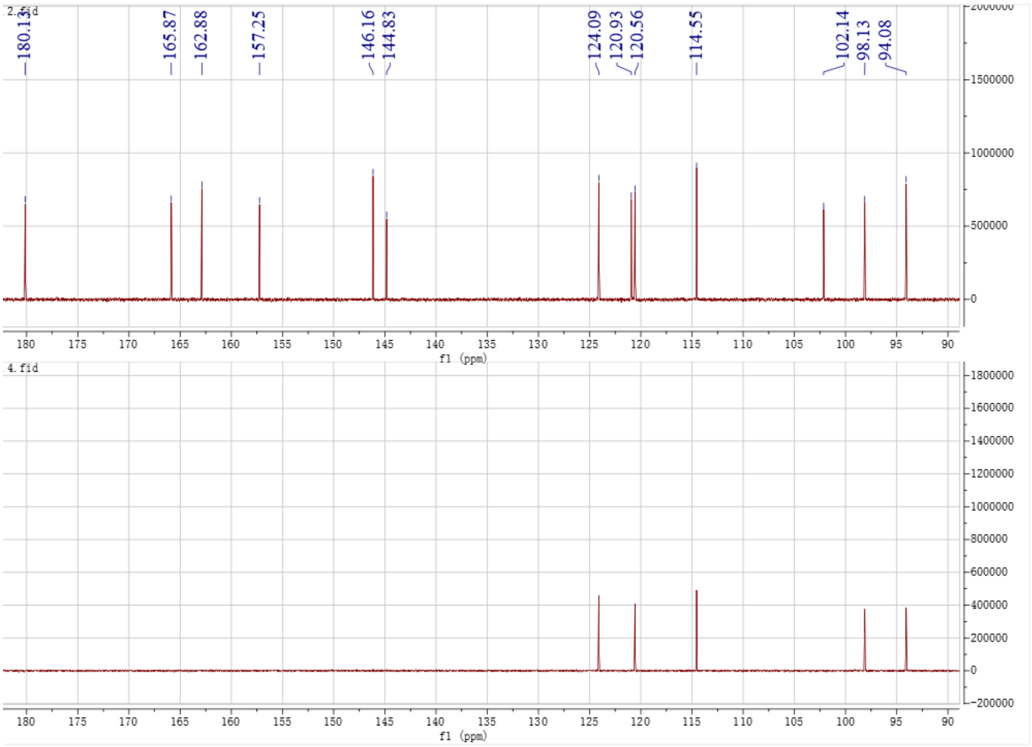

^{13}C-NMR and ^{13}C DEPT-135 spectra of compound **11** (151 MHz, DMSO-d_6)

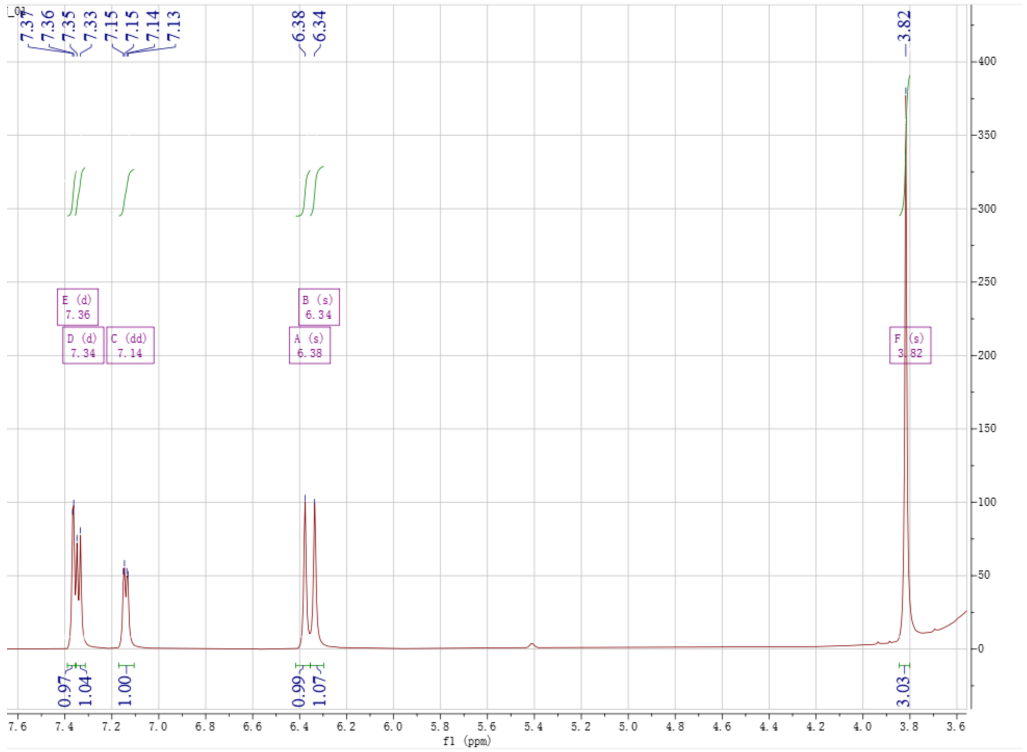

^{1}H-NMR spectrum of compound **12** (600 MHz, DMSO-d_6)

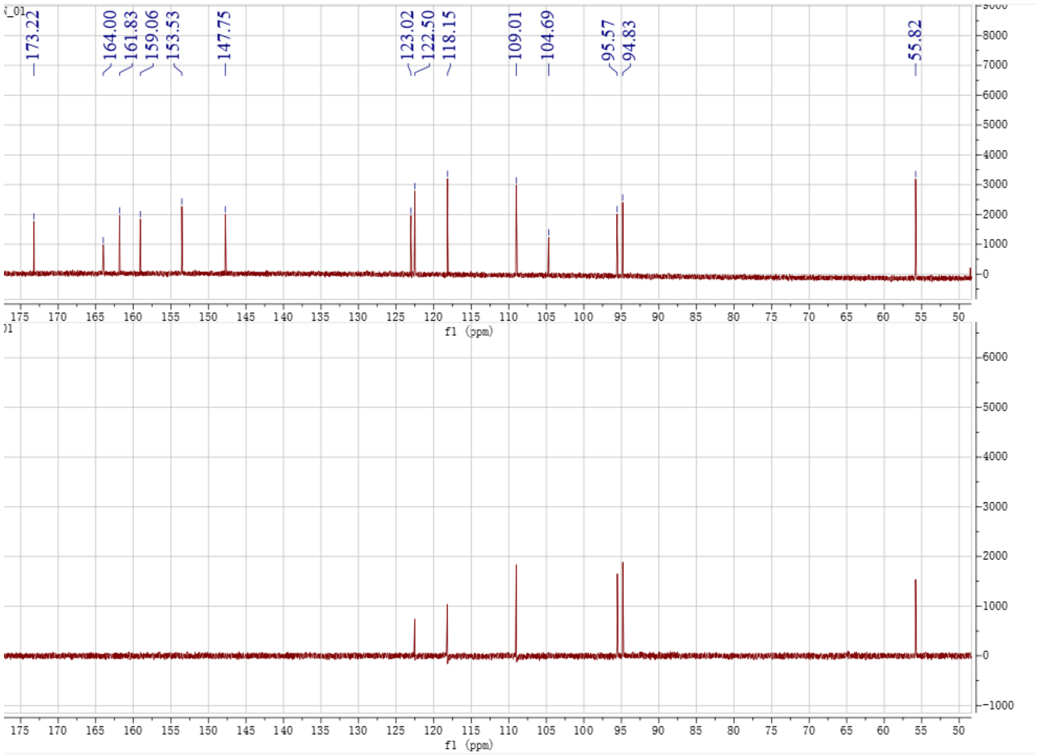

^{13}C-NMR and ^{13}C DEPT-135 spectra of compound **12** (151 MHz, DMSO-d_6)

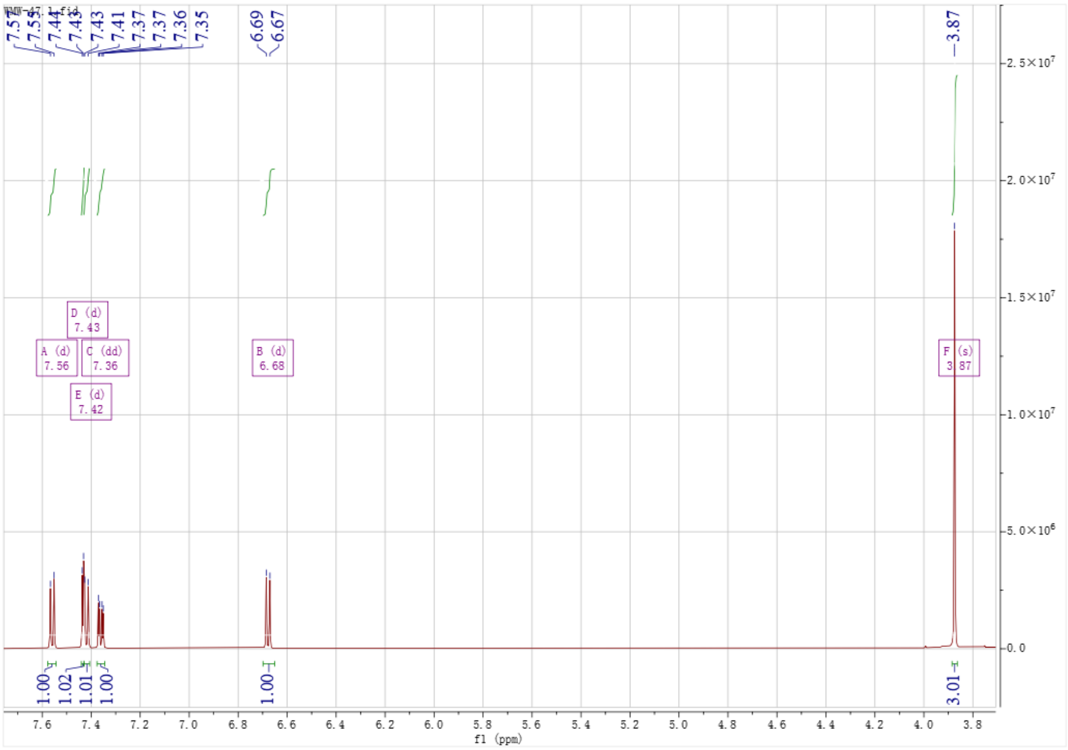

^{1}H-NMR spectrum of compound **13** (600 MHz, DMSO-d_6)

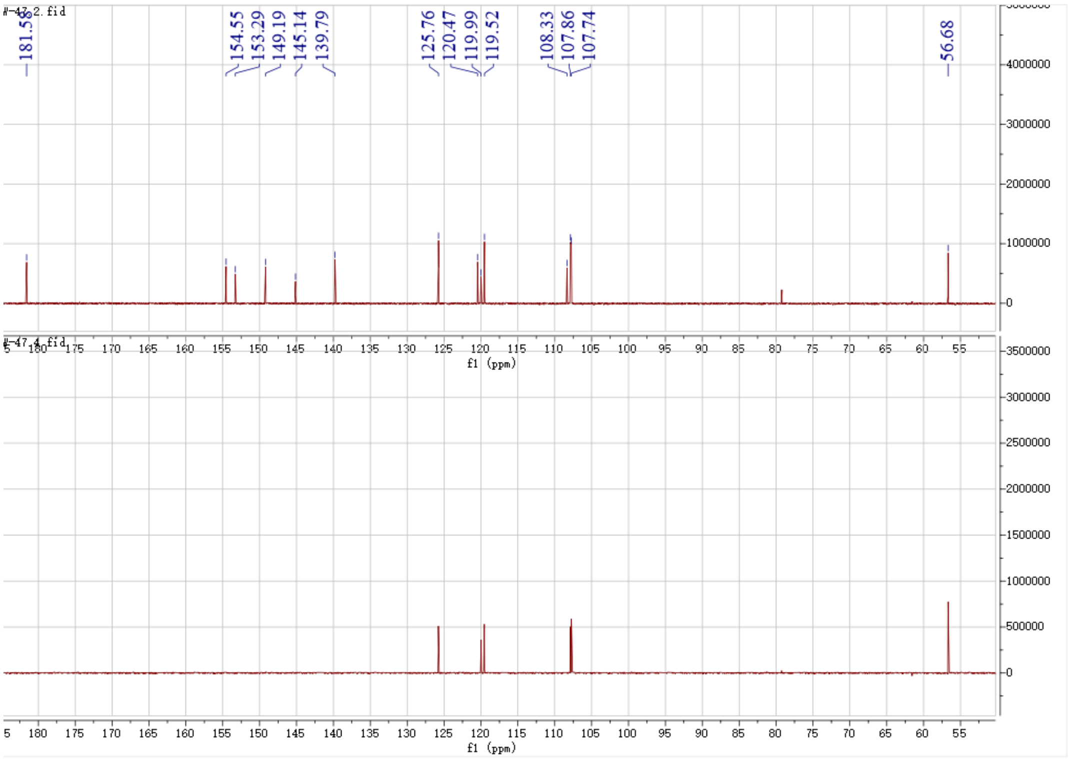

^{13}C-NMR and ^{13}C DEPT-135 spectra of compound **13** (151 MHz, DMSO-d_6)

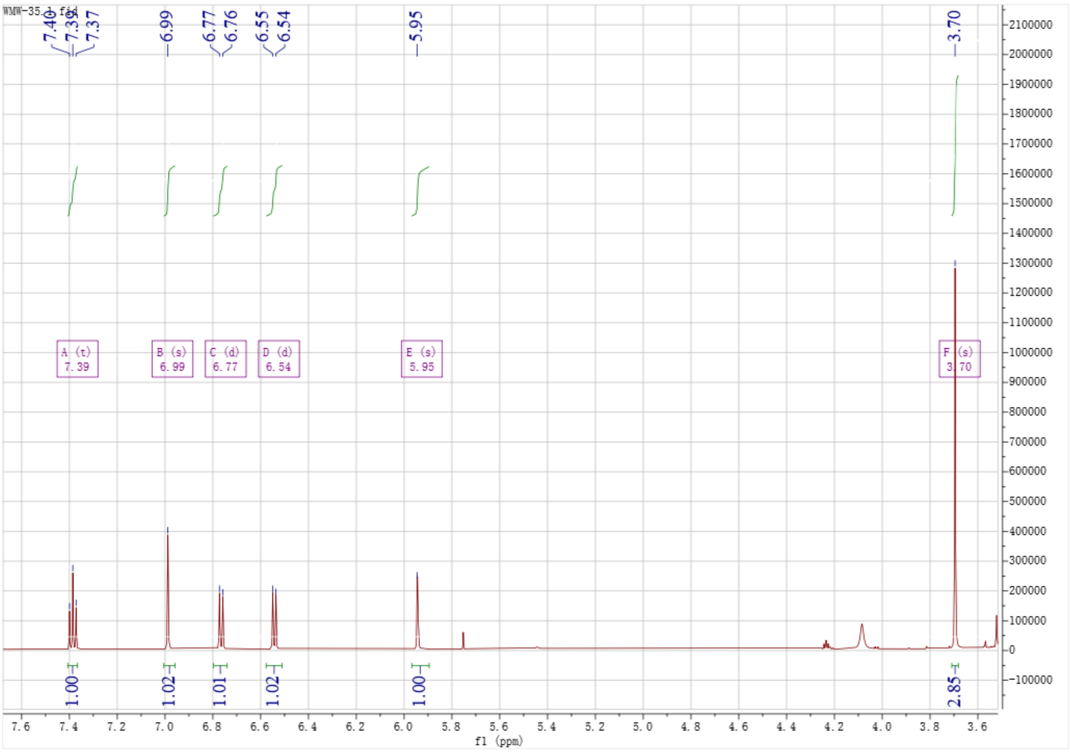

^{1}H-NMR spectrum of compound **14** (600 MHz, DMSO-d_6)

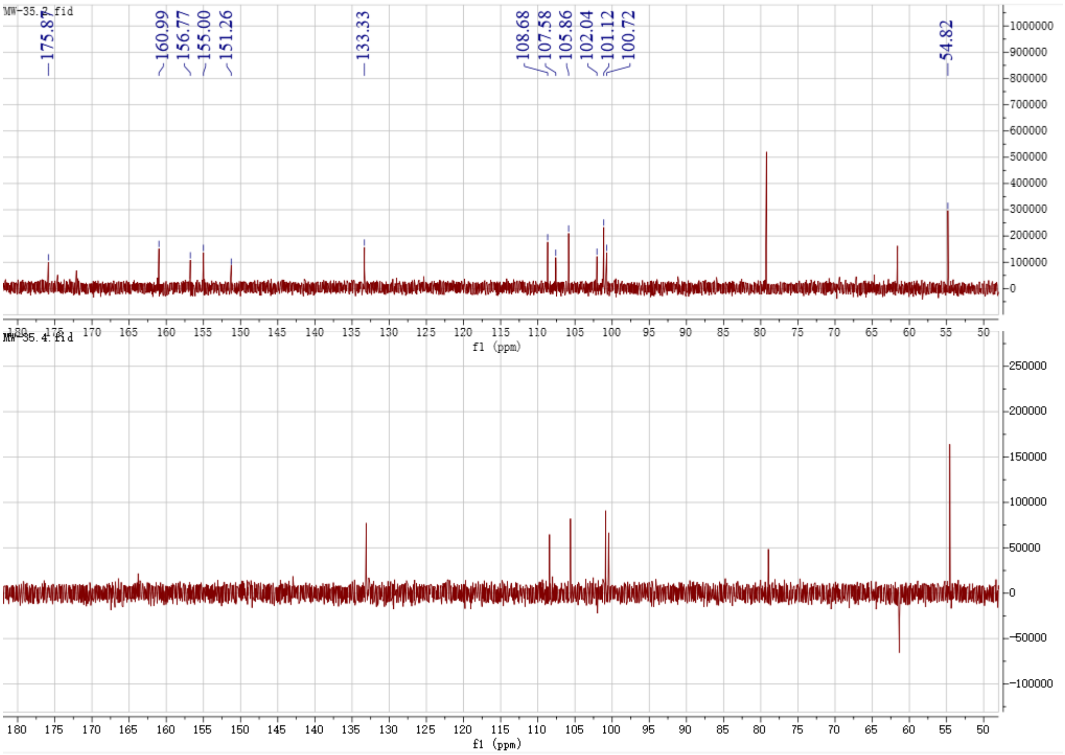

^{13}C-NMR and ^{13}C DEPT-135 spectra of compound **14** (151 MHz, DMSO-d_6)

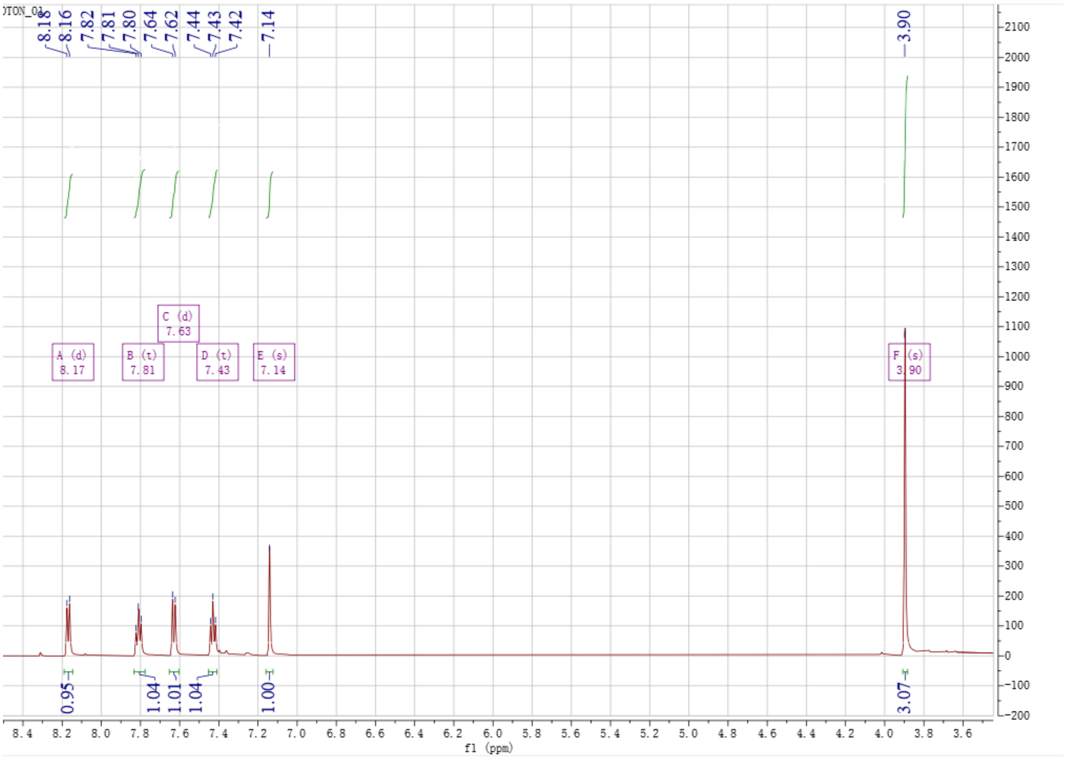

^{1}H-NMR spectrum of compound **15** (600 MHz, DMSO-d_6)

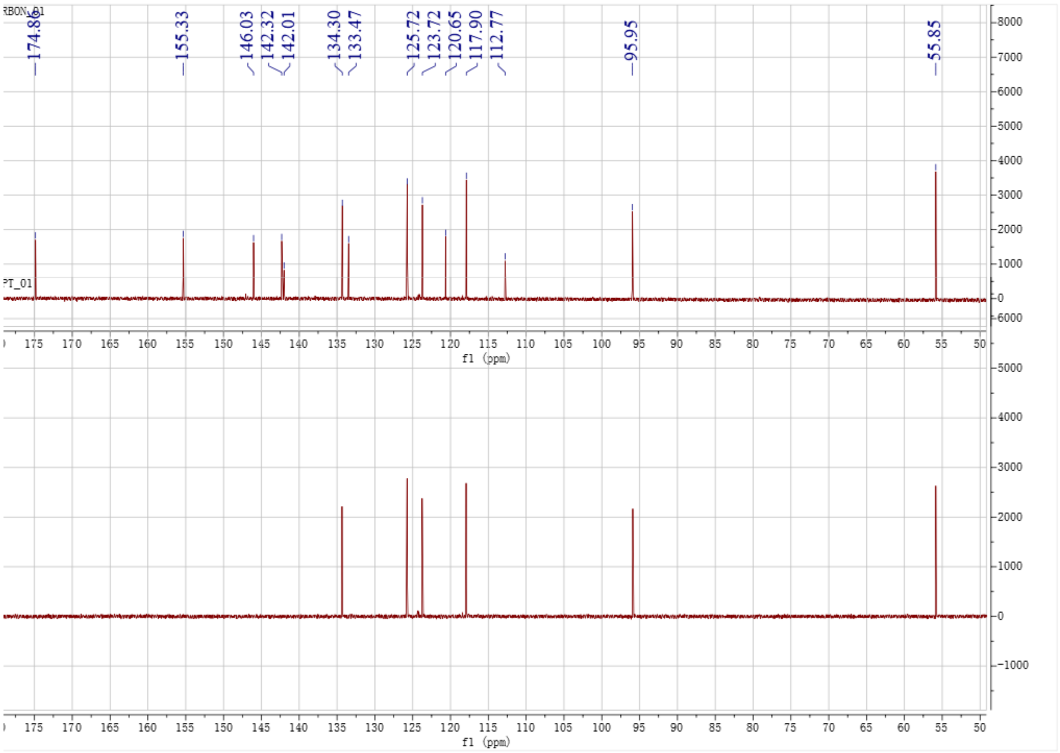

^{13}C-NMR and ^{13}C DEPT-135 spectra of compound **15** (151 MHz, DMSO-d_6)

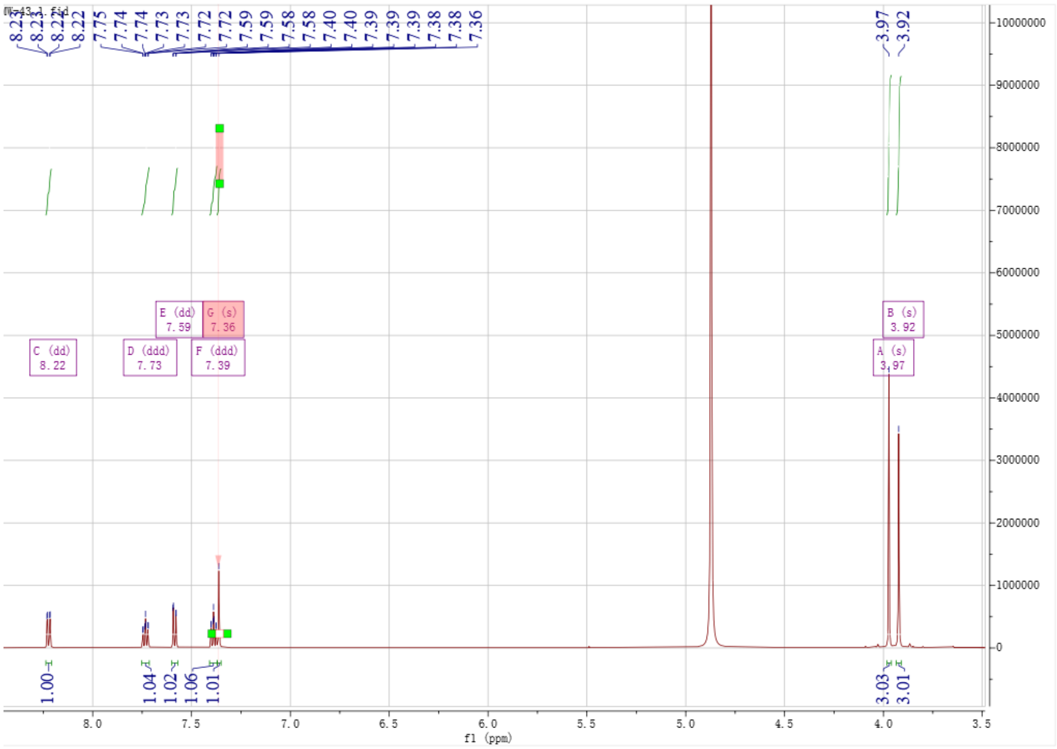

^{1}H-NMR spectrum of compound **16** (600 MHz, CD_3OD)

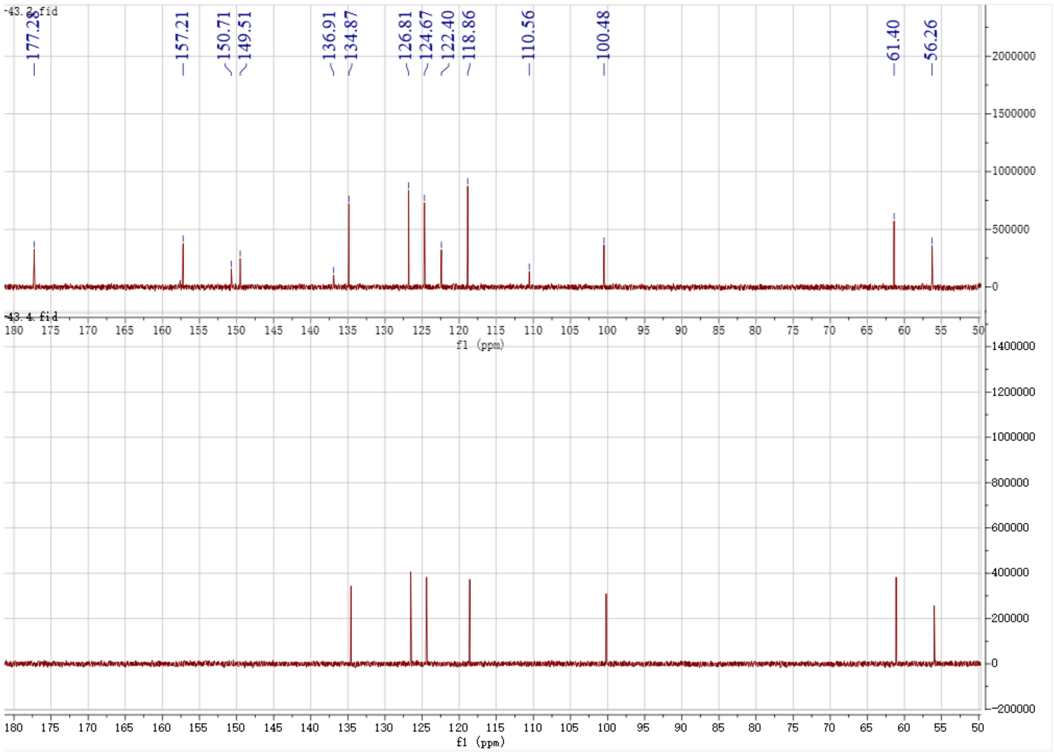

^{13}C-NMR and ^{13}C DEPT-135 spectra of compound **16** (151 MHz, CD_3OD)

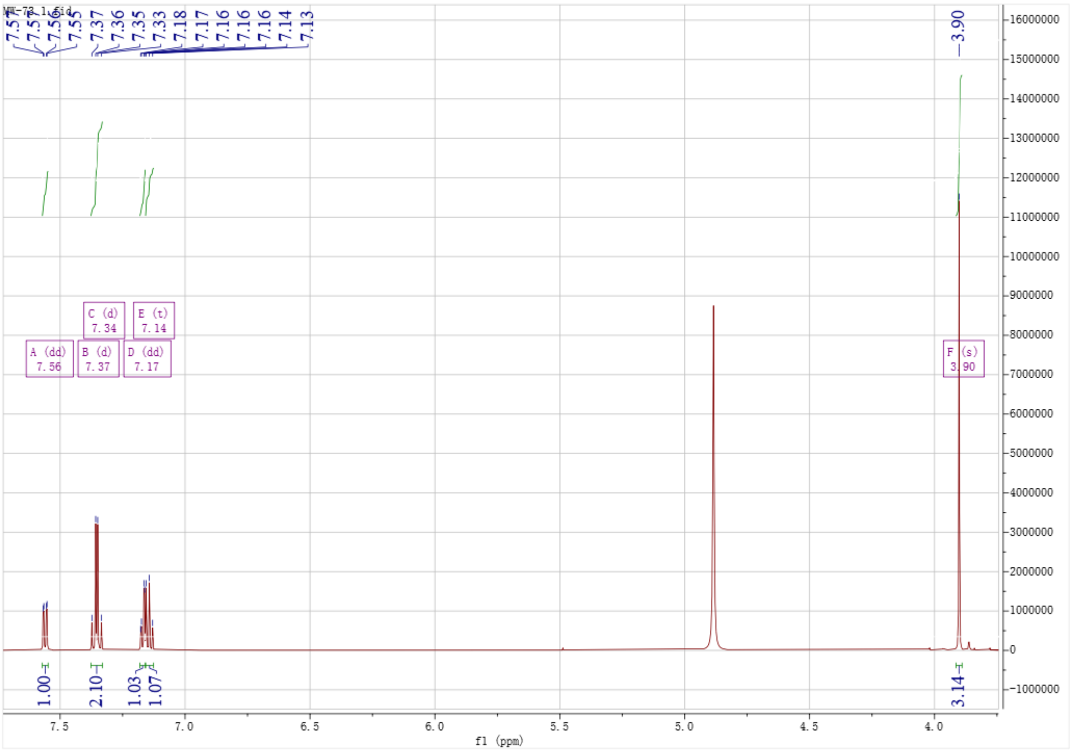

^{1}H-NMR spectrum of compound **17** (600 MHz, CD_3OD)

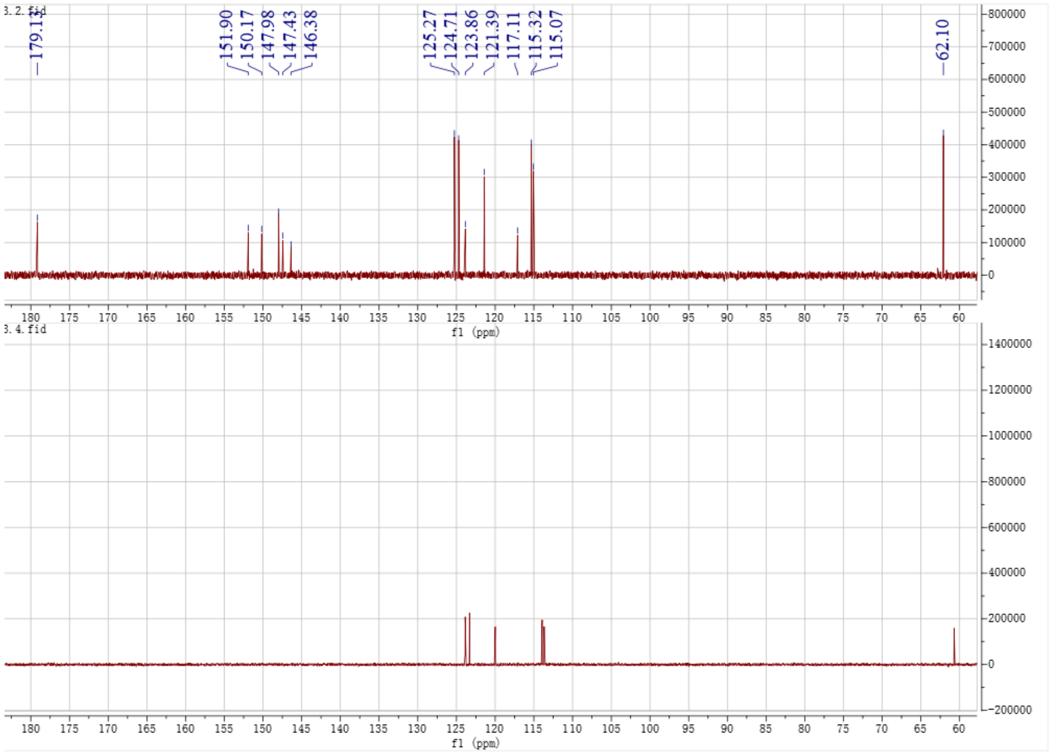

^{13}C-NMR and ^{13}C DEPT-135 spectra of compound **17** (151 MHz, CD_3OD)

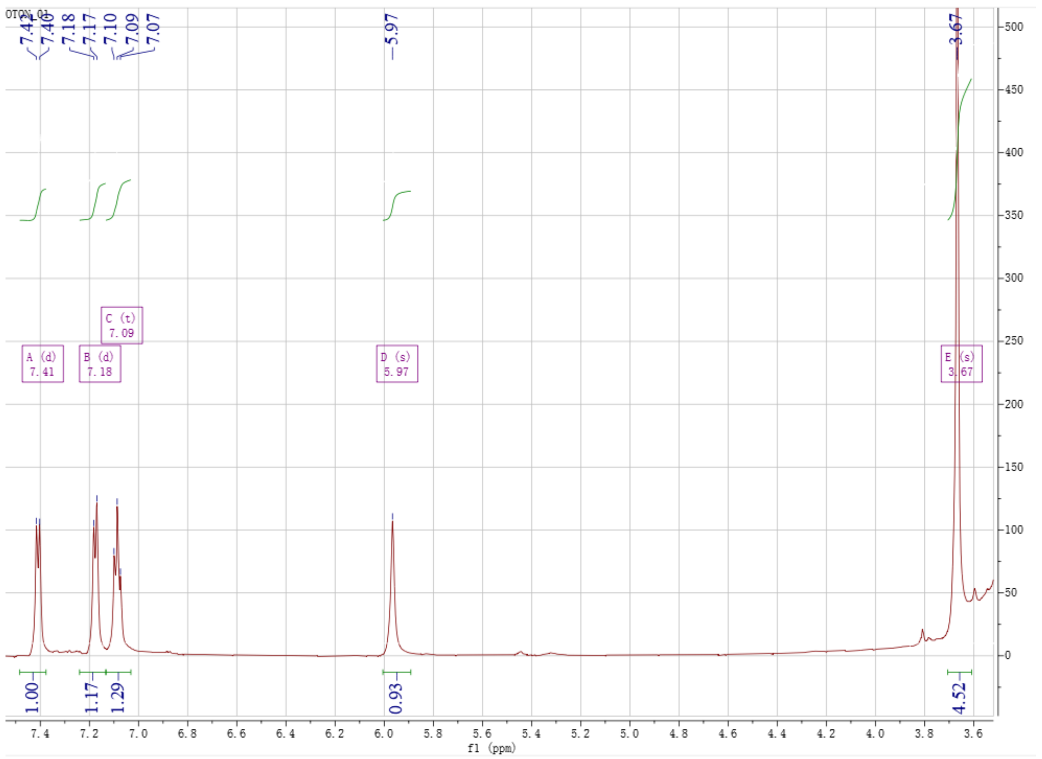

^{1}H-NMR spectrum of compound **18** (600 MHz, CD_3OD)

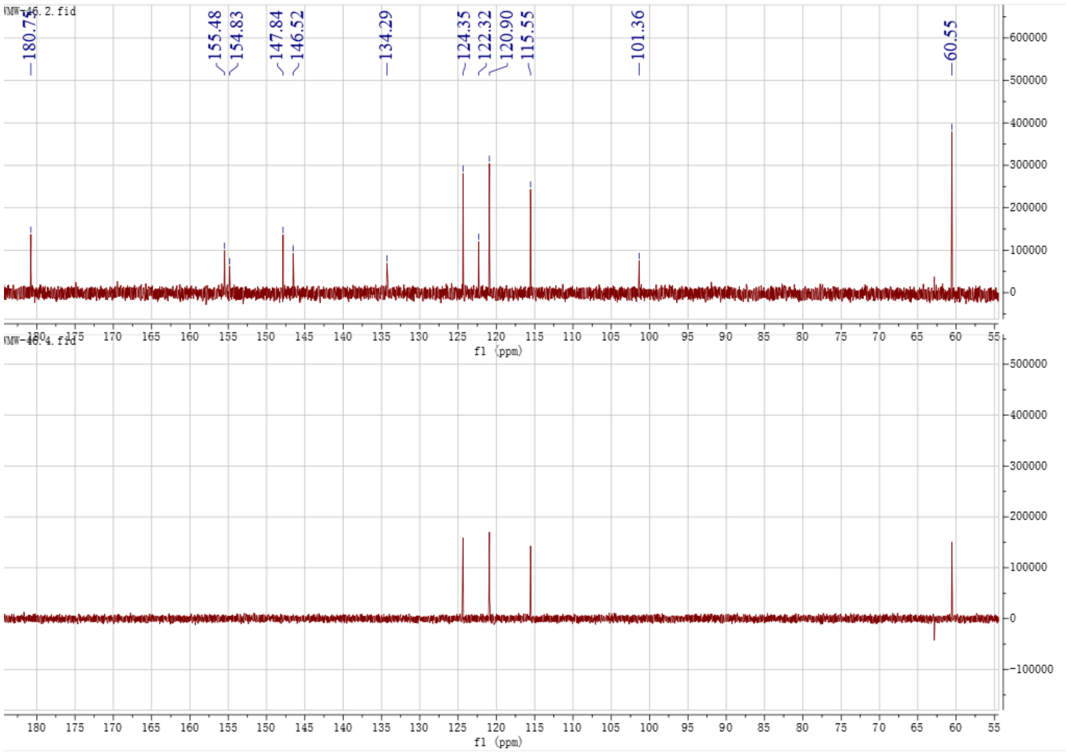

^{13}C-NMR and ^{13}C DEPT-135 spectra of compound **18** (151 MHz, CD_3OD)

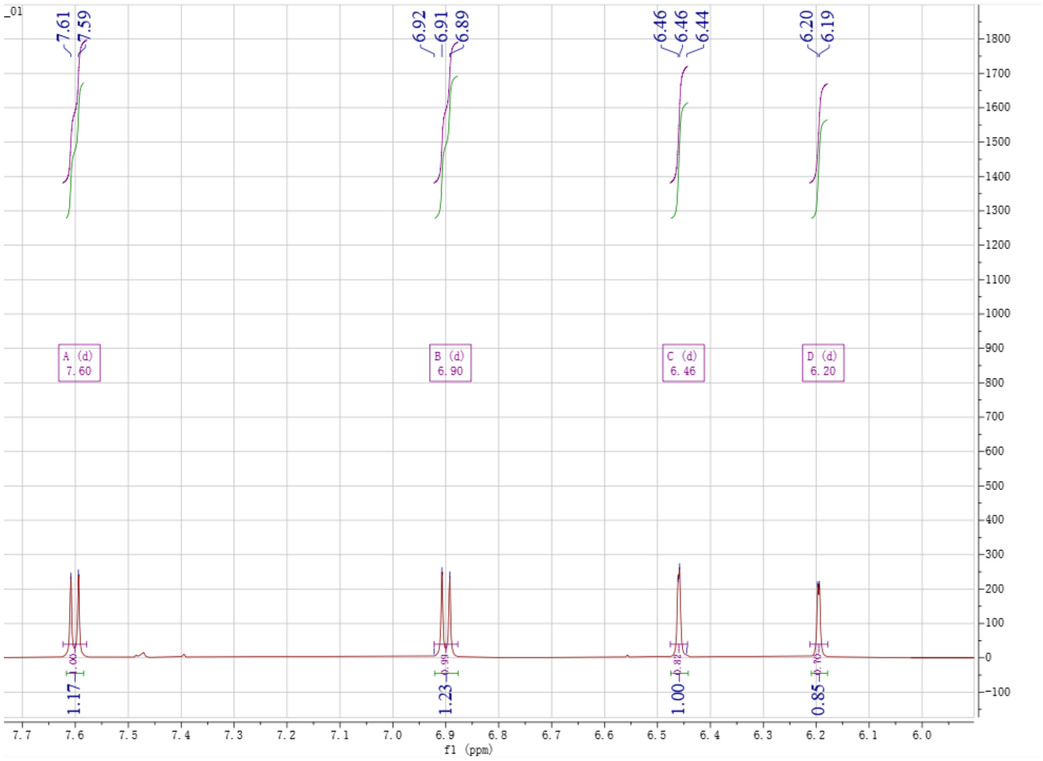

^{1}H-NMR spectrum of compound **19** (600 MHz, CD_3OD)

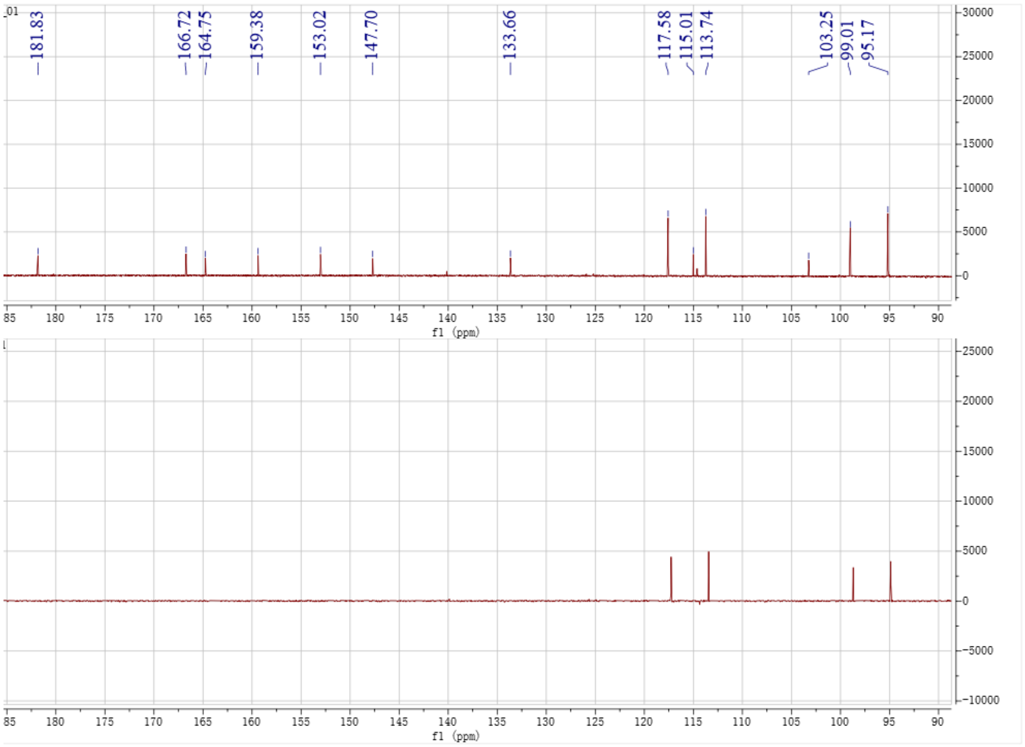

^{13}C-NMR and ^{13}C DEPT-135 spectra of compound **19** (151 MHz, CD_3OD)

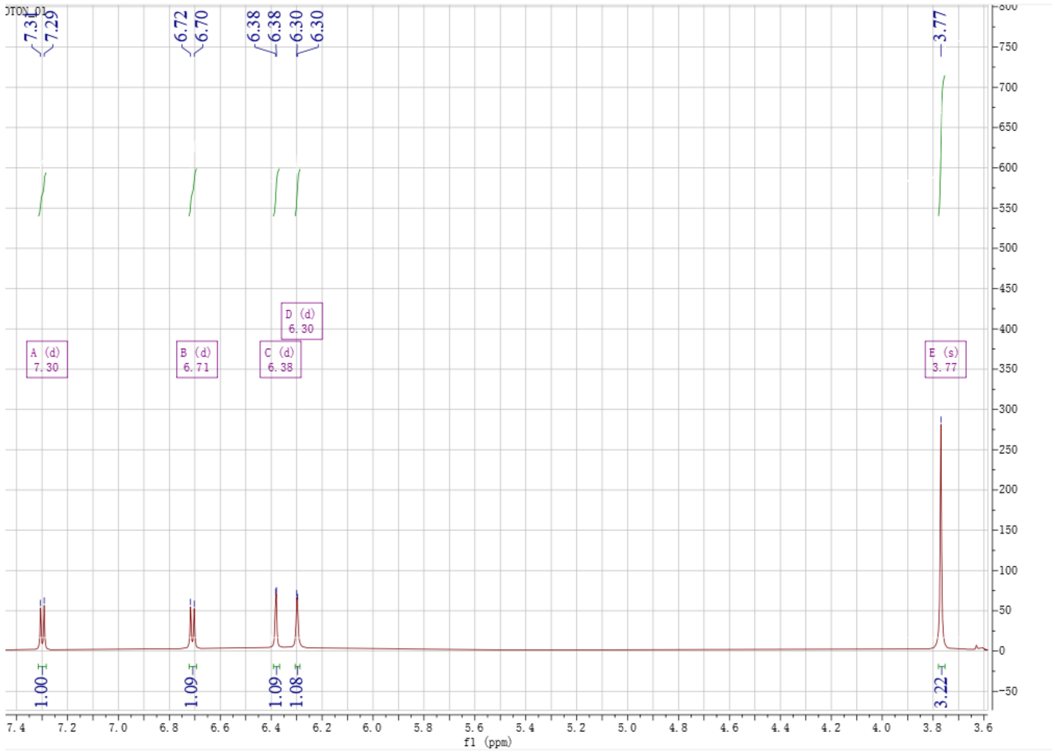

^{1}H-NMR spectrum of compound **20** (600 MHz, DMSO-d_6)

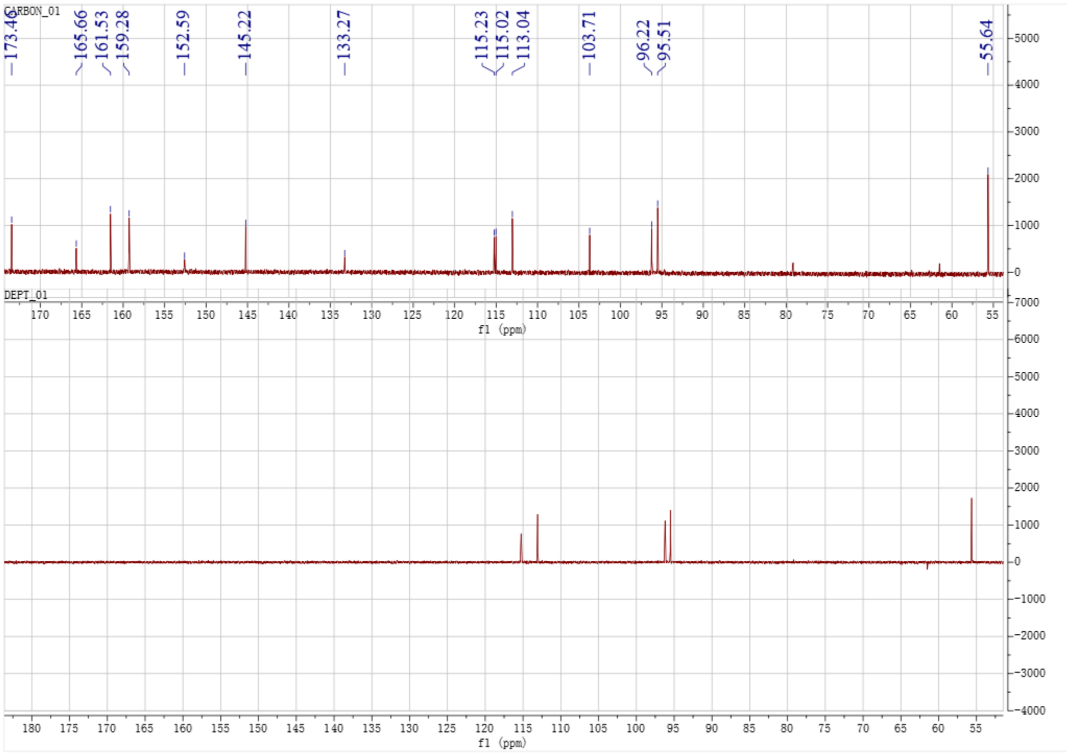

^{13}C-NMR and ^{13}C DEPT-135 spectra of compound **20** (151 MHz, DMSO-d_6)

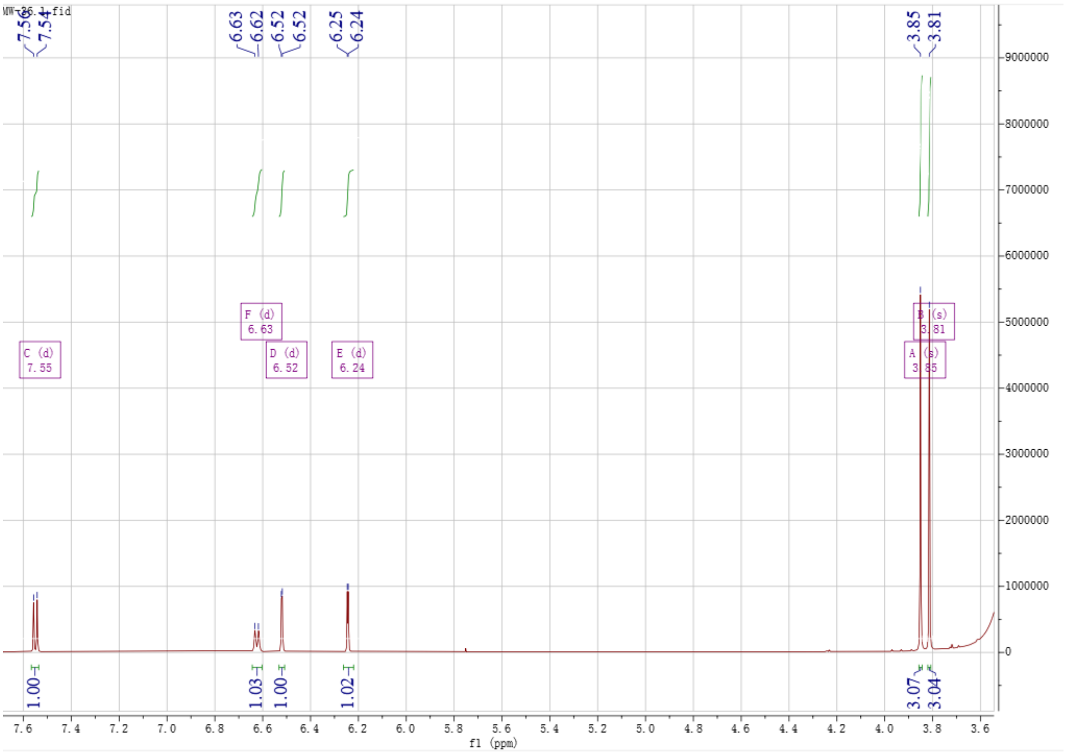

^{1}H-NMR spectrum of compound **21** (600 MHz, DMSO-d_6)

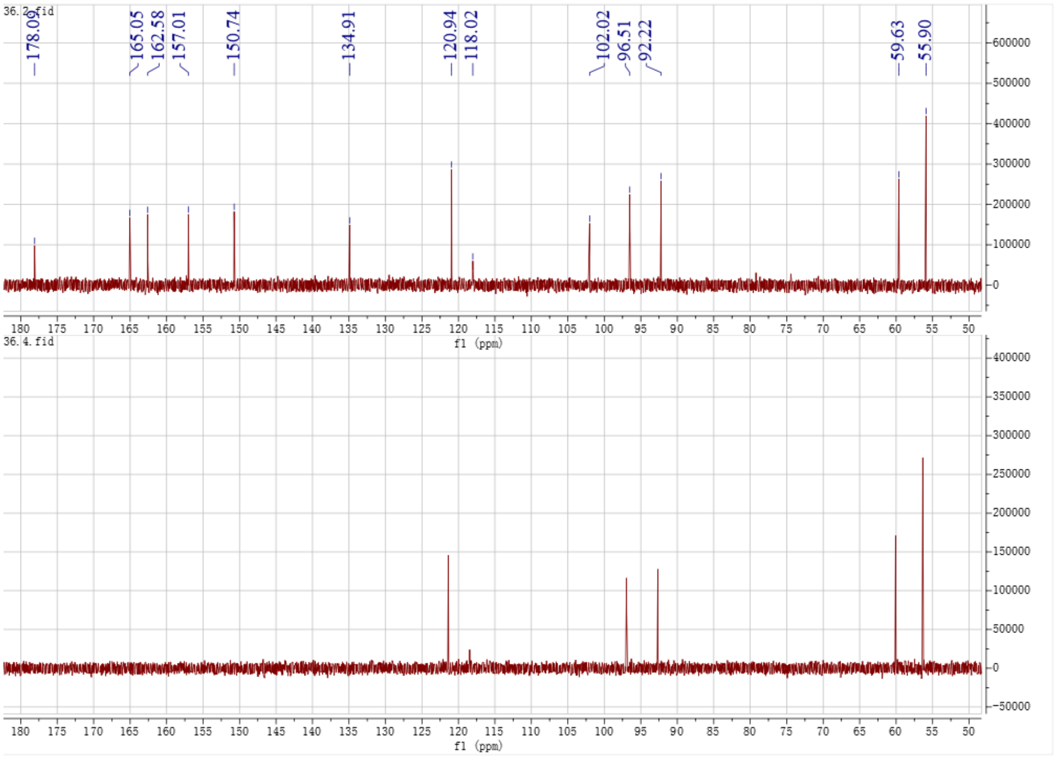

^{13}C-NMR and ^{13}C DEPT-135 spectra of compound **21** (151 MHz, DMSO-d_6)

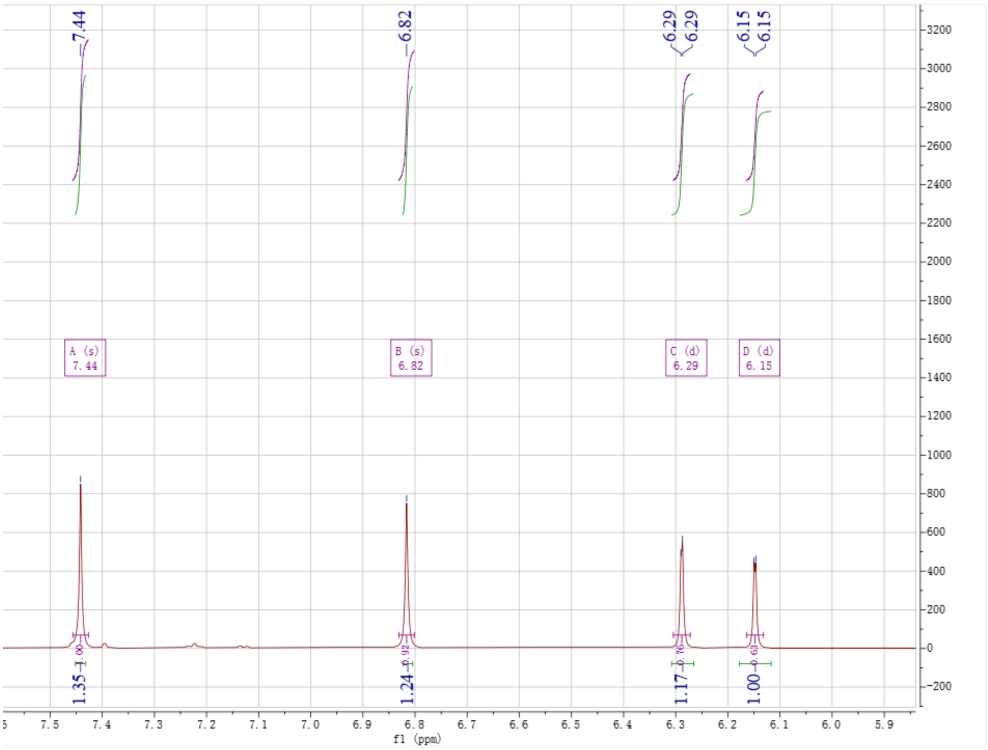

^{1}H-NMR spectrum of compound **22** (600 MHz, CD_3OD)

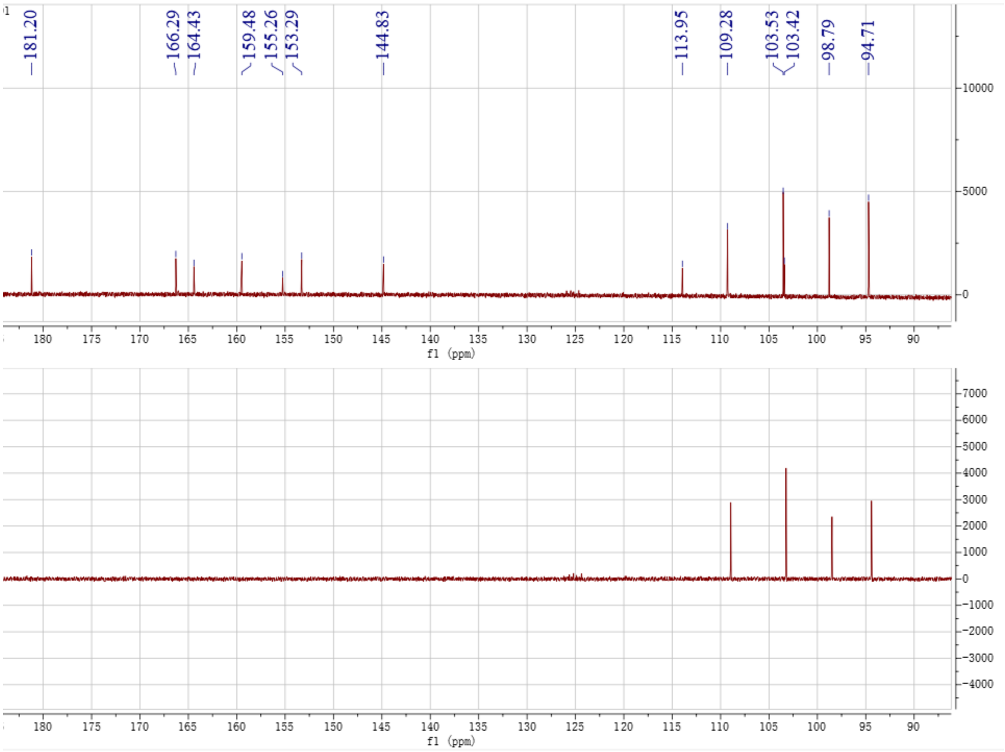

^{13}C-NMR and ^{13}C DEPT-135 spectra of compound **22** (151 MHz, CD_3OD)

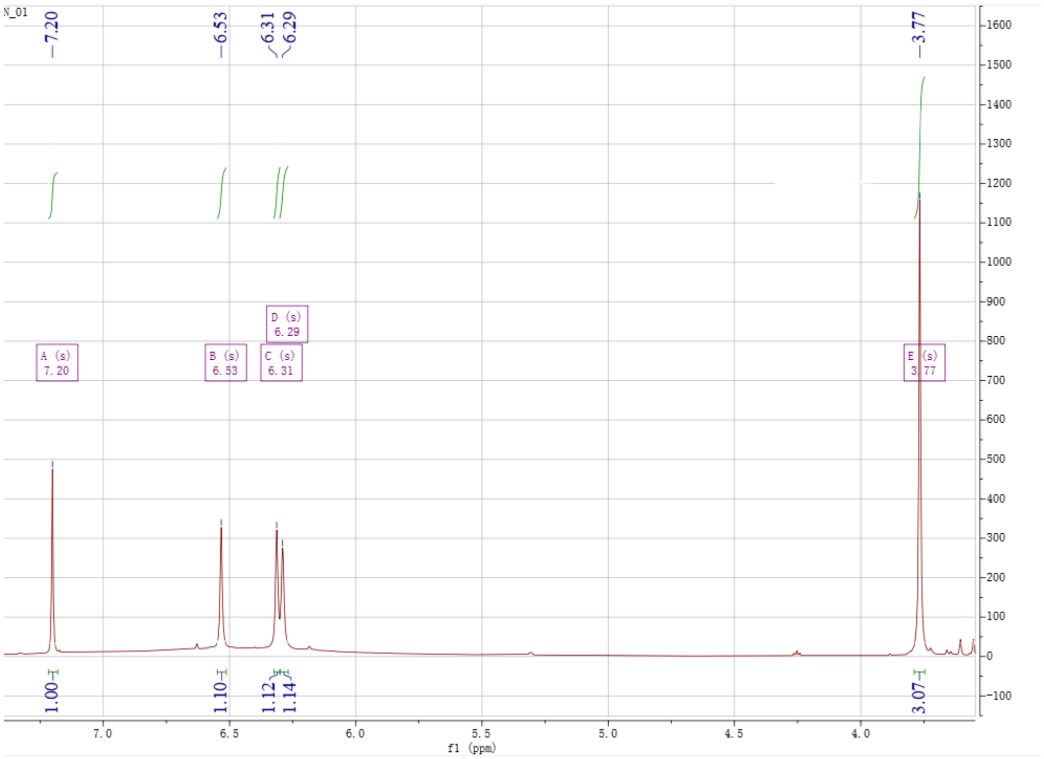

^{1}H-NMR spectrum of compound **23** (600 MHz, DMSO-d_6)

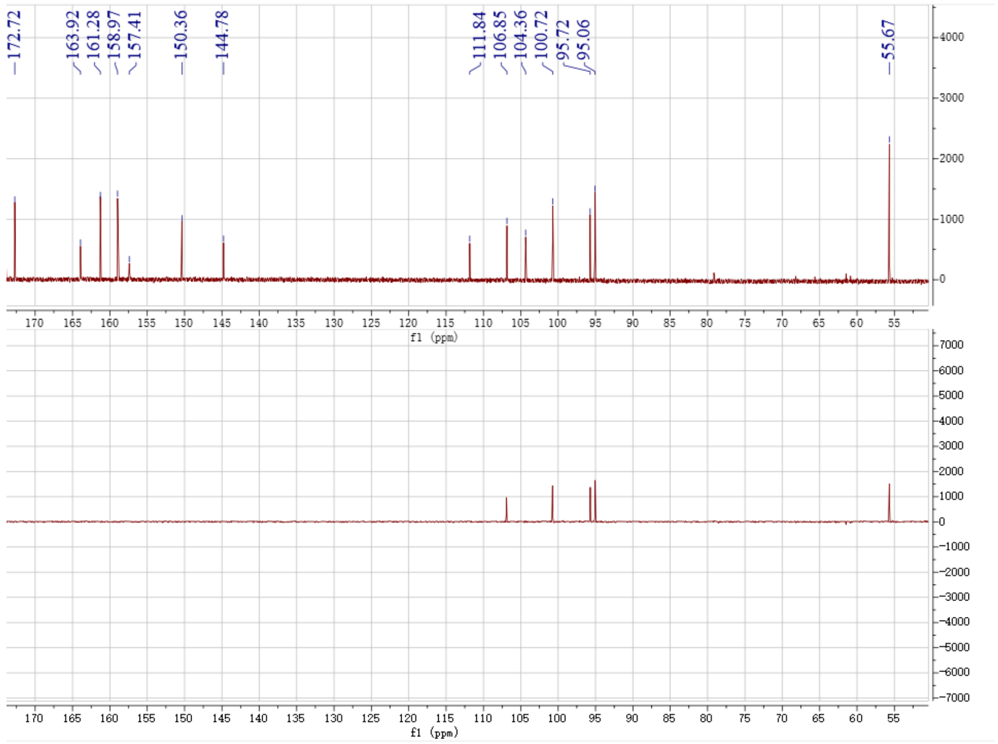

^{13}C-NMR and ^{13}C DEPT-135 spectra of compound **23** (151 MHz, DMSO-d_6)

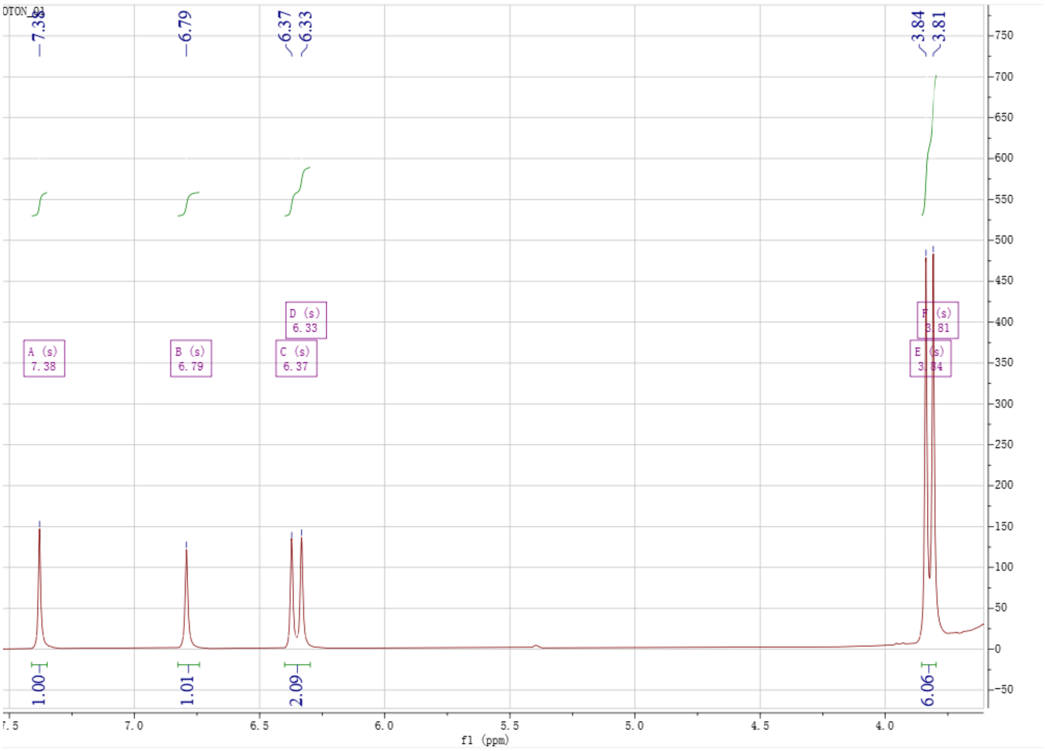

^{1}H-NMR spectrum of compound **24** (600 MHz, DMSO-d_6)

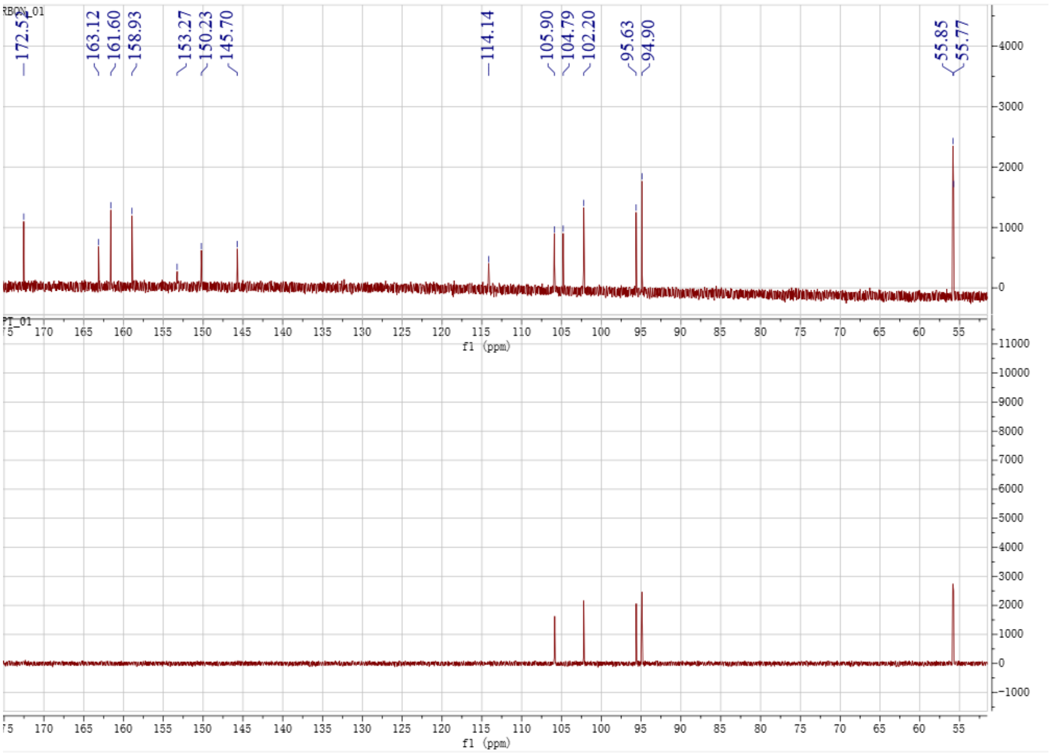

^{13}C-NMR and ^{13}C DEPT-135 spectra of compound **24** (151 MHz, DMSO-d_6)

^{1}H and ^{13}C NMR data of compounds **2**–**24**

2-hydroxyxanthone (**2**): yellow needle crystal, $C_{13}H_8O_3$. ^{1}H NMR (600 MHz, DMSO-d_6) δ 8.11 (dd, J = 7.9, 2.0 Hz, H-8), 7.70 (ddd, J = 8.8, 6.9, 2.0 Hz, H-6), 7.46 (d, J = 8.4 Hz, H-5), 7.43 (d, J = 3.1 Hz, H-4), 7.39 (d, J = 9.0 Hz, H-1), 7.32 (t, J = 7.5 Hz, H-7), 7.22 (dd, J = 9.0, 3.1 Hz, H-3). ^{13}C NMR (151 MHz, DMSO-d_6) δ 177.7 (C-9), 157.0 (C-4b), 155.0 (C-2), 150.8 (C-4a), 135.9 (C-6), 126.8 (C-8), 125.4 (C-3), 124.6 (C-7), 122.9 (C-8b), 121.6 (C-8a), 120.2 (C-4), 118.9 (C-5), 109.5 (C-1).

2-methoxy-3-hydroxyxanthone (**3**): yellow powder, $C_{14}H_{10}O_4$. ^{1}H NMR (600 MHz, DMSO-d_6) δ 8.12 (dd, J = 7.9, 1.7 Hz, H-8), 7.72 (ddd, J = 8.7, 7.1, 1.7 Hz, H-6), 7.53 (dd, J = 8.5, 1.0 Hz, H-5), 7.38 (m, H-7), 7.38 (s, H-1), 6.79 (s, H-4), 3.84 (s, OCH_3-2). ^{13}C NMR (151 MHz, DMSO-d_6) δ 173.7 (C-9), 155.3 (C-4b), 153.2 (C-3), 147.8 (C-2), 133.7 (C-6), 125.6 (C-8), 123.5 (C-7), 121.1 (C-8a), 117.6 (C-5), 110.4 (C-8b), 104.3 (C-1), 102.3 (C-4), 55.5 (2-OCH_3).

1-hydroxy-5-methoxyxanthone (**4**): yellow powder, $C_{14}H_{10}O_4$. ^{1}H NMR (600 MHz, DMSO-d_6) δ 8.22 (dd, J = 8.0, 1.6 Hz, H-8), 7.74 (ddd, J = 8.6, 7.0, 1.8 Hz, H-7), 7.47 (dd, J = 8.4, 1.1 Hz, H-6), 7.36 (ddd, J = 7.5, 7.0, 1.1 Hz, H-3), 7.34 (m, H-4), 7.21 (m, H-2), 3.90 (s, OCH_3-5). ^{13}C NMR (151 MHz, DMSO-d_6) δ 178.6 (C-9), 156.9 (C-1), 151.0 (C-4a), 150.6 (C-5), 147.0 (C-4b), 135.8 (C-3), 127.2 (C-7), 126.6 (C-6), 124.6 (C-8), 122.9 (C-8a), 118.7 (C-2), 117.5 (C-8b), 114.9 (C-4), 61.9 (5-OCH_3).

2,5-dihydroxyxanthone (**5**): yellow powder, $C_{13}H_8O_4$. ^{1}H NMR (600 MHz, DMSO-d_6) δ 7.58 (dd, J = 8.0, 1.6 Hz, H-8), 7.55 (d, J = 9.0 Hz, H-1), 7.47 (d, J = 3.0 Hz, H-4), 7.32 (dd, J = 9.0, 3.0 Hz, H-3), 7.29 (dd, J = 7.7, 1.6 Hz, H-6), 7.22 (t, J = 7.9 Hz, H-7). ^{13}C NMR (151 MHz, DMSO-d_6) δ 176.1 (C-9), 153.9 (C-4a), 149.1 (C-2), 146.6 (C-5), 145.3 (C-4b), 124.5 (C-7), 123.6 (C-3), 121.6 (C-8a), 121.5 (C-6), 119.7 (C-8b), 119.6 (C-4), 115.2 (C-8), 108.5 (C-1).

3-methoxy-5-hydroxyxanthone (**6**): yellow powder, $C_{14}H_{10}O_4$. ^{1}H NMR (600 MHz, DMSO-d_6) δ 7.64 (d, J = 9.2 Hz, H-1), 7.56 (dd, J = 8.0, 1.6 Hz, H-8), 7.55 (d, J = 3.2 Hz, H-4), 7.47 (dd, J = 9.1, 3.2 Hz, H-2), 7.33 (dd, J = 7.8, 1.6 Hz, H-6), 7.22 (t, J = 7.9 Hz, H-7), 3.87 (s, OCH_3-3). ^{13}C NMR (151 MHz, DMSO-d_6) δ 176.1 (C-9), 155.6 (C-3), 150.2 (C-4a), 147.6 (C-5), 145.5 (C-4b), 124.6 (C-2), 123.9 (C-7), 121.6 (C-8a), 121.3 (C-8b), 120.0 (C-1), 120.0 (C-6), 114.4 (C-8), 105.6 (C-4), 55.7 (3-OCH_3).

1,7-dihydroxyxanthone (**7**): yellow powder, $C_{13}H_8O_4$. ^1H NMR (600 MHz, DMSO-d_6) δ 7.65 (t, J = 8.3 Hz, H-3), 7.48 (d, J = 9.0 Hz, H-5), 7.42 (d, J = 3.0 Hz, H-8), 7.34 (dd, J = 9.0, 3.1 Hz, H-6), 6.97 (dd, J = 8.4, 0.9 Hz, H-4, 6.73 (dd, J = 8.2, 0.9 Hz, H-2). ^{13}C NMR (151 MHz, DMSO-d_6) δ 181.6 (C-9), 160.9 (C-1), 155.8 (C-4a), 154.3 (C-7), 149.2 (C-4b), 137.1 (C-3), 125.6 (C-6), 120.4 (C-5), 119.3 (C-8a), 109.6 (C-2), 107.8 (C-4), 107.8 (C-8b), 107.0 (C-8).

1-methoxy-2,5-dihydroxyxanthone (**8**): yellow powder, $C_{14}H_{10}O_5$. ^1H NMR (600 MHz, DMSO-d_6) δ 7.52 (dd, J = 8.0, 1.6 Hz, H-8), 7.39 (d, J = 9.1 Hz, H-3), 7.28 (d, J = 9.1 Hz, H-4), 7.24 (dd, J = 7.7, 1.6 Hz, H-6), 7.17 (t, J = 7.9 Hz, H-7). ^{13}C NMR (151 MHz, DMSO-d_6) δ 175.5 (C-9), 149.5 (C-4a), 146.7 (C-2), 146.6 (C-5), 145.1 (C-1), 144.6 (C-4b), 124.0 (C-3), 123.4 (C-7), 122.6 (C-8a), 119.4 (C-6), 116.1 (C-8b), 114.8 (C-8), 113.7 (C-4), 61.0 (1-OCH_3).

1,5-Dihydroxy-2-methoxyxanthone (**9**): yellow powder, $C_{14}H_{10}O_5$. ^1H NMR (600 MHz, DMSO-d_6) δ 7.58 (d, J = 8.1 Hz, H-8), 7.56 (d, J = 9.1 Hz, H-3), 7.35 (d, J = 7.9 Hz, H-6), 7.26 (t, J = 7.9 Hz, H-7), 7.07 (d, J = 9.0 Hz, H-1), 3.85 (s, OCH_3-2). ^{13}C NMR (151 MHz, DMSO-d_6) δ 182.6 (C-9), 149.7 (C-1), 149.1 (C-4a), 146.5 (C-5), 145.5 (C-4b), 142.0 (C-2), 123.9 (C-7), 122.0 (C-6), 120.9 (C-8), 120.2 (C-8a), 114.4 (C-3), 108.3 (C-8b), 105.9 (C-4), 56.7 (2-OCH_3).

1,2-dimethoxy-5-hydroxyxanthone (**10**): yellow powder, $C_{15}H_{12}O_5$. ^1H NMR (600 MHz, CD_3OD) δ 7.61 (d, J = 9.3 Hz, H-3), 7.58 (dd, J = 7.5, 2.2 Hz, H-8), 7.46 (d, J = 9.3 Hz, H-4)7.21 (dd, J = 7.8, 2.1 Hz, H-6), 7.19 (t, J = 7.8 Hz, H-7), 3.93 (s, OCH3-1), 3.95 (s, OCH3-2). ^{13}C NMR (151 MHz, CD_3OD) δ 177.3 (C-9), 151.0 (C-2), 149.1 (C-1), 148.5 (C-5), 148.0 (C-4a), 145.8 (C-4b), 123.6 (C-7), 122.7 (C-8a), 120.6 (C-3), 120.2 (C-6), 116.2 (C-8b), 114.0 (C-8), 113.6 (C-4), 60.7 (1-OCH3), 56.3 (2-OCH3).

1,3,5-trihydroxyxanthone (**11**): yellow powder, $C_{13}H_8O_5$. ^1H NMR (600 MHz, DMSO-d_6) δ 7.53 (dd, J = 7.9, 1.6 Hz, H-8), 7.29 (dd, J = 7.8, 1.6 Hz, H-6), 7.23 (t, J = 7.9 Hz, H-7), 6.41 (d, J = 2.1 Hz, H-4), 6.20 (d, J = 2.1 Hz, H-2). ^{13}C NMR (151 MHz, DMSO-d_6) δ 180.1 (C-9), 165.9 (C-3), 162.9 (C-1), 157.2 (C-4a), 146.2 (C-5), 144.8 (C-4b), 124.1 (C-7), 120.9 (C-6), 120.6 (C-8a), 114.5 (C-8), 102.1 (C-8b), 98.1 (C-2), 94.1 (C-4).

1-methoxy-3,7-dihydroxyxanthone (**12**): yellow powder, $C_{14}H_{10}O_5$. ^1H NMR (600 MHz, DMSO-d_6) δ 7.36 (d, J = 2.9 Hz, H-8), 7.34 (d, J = 8.9 Hz, H-5), 7.14 (dd, J = 8.9, 2.9 Hz, H-6), 6.38 (s, H-2), 6.34 (s, H-4), 3.82 (s, OCH_3-1). ^{13}C NMR (151 MHz,

DMSO-d_6) δ 173.2 (C-9), 164.0 (C-3), 161.8 (C-1), 159.1 (C-4a), 153.5 (C-7), 147.7 (C-4b), 123.0 (C-8a), 122.5 (C-6), 118.1 (C-5), 109.0 (C-8), 104.7 (C-8b), 95.6 (C-2), 94.8 (C-4), 55.8 (1-OCH_3).

1,7-dihydroxy-4-methoxyxanthone (**13**): yellow powder, $C_{14}H_{10}O_5$. ^{1}H NMR (600 MHz, DMSO-d_6) δ 7.56 (d, J = 9.1 Hz, H-5), 7.43 (d, J = 3.0 Hz, H-8), 7.42 (d, J = 8.9 Hz, H-3), 7.36 (dd, J = 9.0, 3.0 Hz, H-3), 6.68 (d, J = 8.9 Hz, H-2), 3.87 (s, OCH_3-4). ^{13}C NMR (151 MHz, DMSO-d_6) δ 181.6 (C-9), 154.5 (C-1), 153.3 (C-7), 149.2 (C-4b), 145.1 (C-4a), 139.8 (C-4), 125.8 (C-6), 120.5 (C-8a), 120.0 (C-3), 119.5 (C-5), 108.3 (C-8b), 107.9 (C-8), 107.7 (C-2), 56.7 (4-OCH3).

1,6-dihydroxy-7-methoxyxanthone (**14**): yellow powder, $C_{14}H_{10}O_5$. ^{1}H NMR (600 MHz, DMSO-d_6) δ 7.39 (t, J = 8.2 Hz, H-3), 6.99 (s, H-8), 6.77 (d, J = 8.3 Hz, H-4), 6.54 (d, J = 8.1 Hz, H-2), 5.95 (s, H-5), 3.70 (s, OCH_3-7). ^{13}C NMR (151 MHz, DMSO-d_6) δ 175.9 (C-9), 161.0 (C-1), 156.8 (C-4a), 155.0 (C-4b), 151.3 (C-6), 133.3 (C-3), 108.7 (C-8a), 107.6 (C-2), 105.9 (C-8b), 102.0 (C-4), 101.1 (C-5), 100.7 (C-8), 54.8 (7-OCH_3).

2-methoxy-3,4-dihydroxyxanthone (**15**): yellow powder, $C_{14}H_{10}O_5$. ^{1}H NMR (600 MHz, DMSO-d_6) δ 8.17 (d, J = 7.9 Hz, H-8), 7.81 (t, J = 7.8 Hz, H-6), 7.63 (d, J = 8.4 Hz, H-5), 7.43 (t, J = 7.5 Hz, H-7), 7.14 (s, H-1), 3.90 (s, OCH_3-2). ^{13}C NMR (151 MHz, DMSO-d_6) δ 174.9 (C-9), 155.3 (C-4b), 146.0 (C-2), 142.3 (C-4a), 142.0(C-4), 134.3 (C-6), 133.5 (C-3), 125.7

2,4-dimethoxy-3-hydroxyxanthone (**16**): yellow powder, $C_{15}H_{12}O_5$. ^{1}H NMR (600 MHz, CD_3OD) δ 8.22 (dd, J = 7.9, 1.8 Hz, H-8), 7.73 (ddd, J = 8.7, 7.0, 1.8 Hz, H-6), 7.59 (dd, J = 8.4, 1.1 Hz, H-5), 7.39 (ddd, J = 8.1, 7.0, 1.1 Hz, H-7), 7.36 (s, H-1), 3.97 (s, OCH_3-4), 3.92 (s, OCH_3-3). ^{13}C NMR (151 MHz, CD_3OD) δ 177.3 (C-9), 157.2 (C-4b), 150.7 (C-4a), 149.5 (C-3), 136.9 (C-4), 134.9 (C-6), 126.8 (C-8), 124.7 (C-7), 122.4 (C-8a), 118.9 (C-5), 110.6 (C-9a), 100.5 (C-1), 61.4 (4-OCH_3), 56.3 (2-OCH_3).

3,5-dihydroxy-4-methoxyxanthone (**17**): yellow powder, $C_{14}H_{10}O_5$. ^{1}H NMR (600 MHz, CD_3OD) δ 7.56 (dd, J = 7.6, 1.9 Hz, H-8), 7.37 (d, J = 9.1 Hz, H-1), 7.34 (d, J = 9.1 Hz, H-2), 7.17 (dd, J = 7.8, 1.9 Hz, H-6), 7.14 (t, J = 7.7 Hz, H-7), 3.90 (s, OCH_3-4). ^{13}C NMR (151 MHz, CD_3OD) δ 179.1 (C-9), 151.9 (C-4a), 150.2 (C-5), 148.0 (C-4), 147.4 (C-3), 146.4 (C-4b), 125.3 (C-1), 124.7 (C-7), 123.9 (C-8a), 121.4 (C-6), 117.1 (C-8b), 115.3 (C-8), 115.1 (C-2), 62.1 (4-OCH_3).

1,3,5-trihydroxy-2-methoxyxanthone (**18**): yellow powder, $C_{14}H_{10}O_6$. ^{1}H NMR (600 MHz, DMSO-d_6) δ 7.41 (d, J = 7.9 Hz, H-8), 7.18 (d, J = 7.8 Hz, H-6), 7.09 (t, J = 7.8 Hz, H-7), 5.97 (s, H-4), 3.67 (s, OCH_3-2). ^{13}C NMR (151 MHz, DMSO-d_6) δ 176.5 (C-9), 153.9 (C-1), 152.6 (C-3), 146.0 (C-5), 144.2 (C-4b), 133.0 (C-2), 122.6 (C-7), 121.1 (C-8a), 118.9 (C-6), 113.7 (C-8), 96.4 (C-4), 58.8 (2-OCH_3).

1,3,5,6-tetrahydroxyxanthone (**19**): yellow powder, $C_{13}H_8O_6$. ^{1}H NMR (600 MHz, CD_3OD) δ 7.60 (d, J = 8.8 Hz, H-8), 6.90 (d, J = 8.7 Hz, H-7), 6.46 (d, J = 1.9 Hz, H-4), 6.20 (d, J = 2.1 Hz, H-2). ^{13}C NMR (151 MHz, CD_3OD) δ 181.8 (C-9), 166.7 (C-3), 164.8 (C-1), 159.4 (C-4a), 153.0 (C-6), 147.7 (C-4b), 133.7 (C-5) 117.6 (C-8), 115.0 (C-8a), 113.7 (C-7), 103.2 (C-8b), 99.0 (C-2), 95.2 (C-4).

1-methoxy-3,5,6-trihydroxyxanthone (**20**): yellow powder, $C_{14}H_{10}O_6$. ^{1}H NMR (600 MHz, DMSO-d_6) δ 7.30 (d, J = 8.6 Hz, H-8), 6.71 (d, J = 8.6 Hz, H-7), 6.38 (d, J = 2.1 Hz, H-4), 6.30 (d, J = 2.1 Hz, H-2), 3.77 (s, OCH_3-1). ^{13}C NMR (151 MHz, DMSO-d_6) δ 173.5 (C-9), 165.7 (C-3), 161.5 (C-1), 159.3 (C-4a), 152.6 (C-6), 145.2 (C-4b), 133.3 (C-5), 115.2 (C-8), 115.0 (C-8a), 113.0 (C-7), 103.7 (C-8b), 96.2 (C-2), 95.5 (C-4), 55.6 (1-OCH_3).

1,6-dihydroxy-3,5-dimethoxyxanthone (**21**): yellow powder, $C_{15}H_{12}O_6$. ^{1}H NMR (600 MHz, DMSO-d_6) δ 7.55 (d, J = 9.0 Hz, H-8), 6.63 (d, J = 9.0 Hz, H-7), 6.52 (d, J = 2.4 Hz, H-4), 6.24 (d, J = 2.3 Hz, H-2), 3.85 (s, OCH_3-5), 3.81 (s, OCH_3-3). ^{13}C NMR (151 MHz, DMSO-d_6) δ 178.1 (C-9), 165.1 (C-3), 162.6 (C-1), 157.0 (C-4a), 150.7 (C-6), 134.9 (C-5), 120.9 (C-8), 118.0 (C-7), 102.0 (C-8b), 96.5 (C-2), 92.2 (C-4), 59.6 (5-OCH_3), 55.9 (3-OCH_3).

1,3,6,7-tetrahydroxyxanthone (**22**): yellow powder, $C_{13}H_8O_6$. ^{1}H NMR (600 MHz, CD_3OD) δ 7.44 (s, H-8), 6.82 (s, H-5), 6.29 (d, J = 2.0 Hz, H-4), 6.15 (d, J = 2.1 Hz, H-2). ^{13}C NMR (151 MHz, CD_3OD) δ 181.2 (C-9), 166.3 (C-3), 164.4 (C-1), 159.5 (C-4a), 155.3 (C-6), 153.3 (C-4b), 144.8 (C-7), 113.9 (C-8a), 109.3 (C-8), 103.5 (C-5), 103.4 (C-8b), 98.8 (C-2), 94.7 (C-4).

1-methoxy-3,6,7-trihydroxyxanthone (**23**): yellow powder, $C_{14}H_{10}O_6$. ^{1}H NMR (600 MHz, DMSO-d_6) δ 7.20 (s, H-8), 6.53 (s, H-5), 6.31 (s, H-4), 6.29 (s, H-2), 3.77 (s, OCH_3-1). ^{13}C NMR (151 MHz, DMSO-d_6) δ 172.7 (C-9), 163.9 (C-3), 161.3 (C-1), 159.0 (C-4a), 157.4 (C-6), 150.4 (C-7), 144.8 (C-4b), 111.8 (C-8a), 106.9 (C-8), 104.4 (C-8b), 100.7 (C-5), 95.7 (C-2), 95.1 (C-4), 55.7 (1-OCH_3).

1,7-dimethoxy-3,6-dihydroxyxanthone (**24**): yellow powder, $C_{15}H_{12}O_6$. ^1H NMR (600 MHz, DMSO-d_6) δ 7.38 (s, H-8), 6.79 (s, H-5), 6.37 (s, H-4), 6.33 (s, H-2), 3.84 (s, OCH_3-7), 3.81 (s, OCH_3-1). ^{13}C NMR (151 MHz, DMSO-d_6) δ 172.5 (C-9), 163.1 (C-3), 161.6 (C-1), 158.9 (C-4a), 153.3 (C-4b), 150.2 (C-6), 145.7 (C-7), 114.1 (C-8a), 105.9 (C-8), 104.8 (C-8b), 102.2 (C-5), 95.6 (C-2), 94.9 (C-4), 55.8 (1-OCH_3), 55.8 (7-OCH_3).

A survey of natural compounds **2–24**

NO.	Compound	Origin	pharmacological activity	Ref.
**2**	2-hydroxyxanthone	*Mammea siamensis*		[1]
		Garcinia schomburgkiana		[2]
		Mammea neurophylla		[3]
		Calophyllum inophyllum		[4]
		Kayea assamica		[5]
		Calophyllum teysmannii		[6]
		Hypericum roeperanum		[7]
**3**	3-hydroxy-2-methoxyxanthone	*Caesalpinia sappan*		[8]
		Psorospermum febrifugum		[9]
**4**	1-hydroxy-5-methoxyxanthone	*Calophyllum thwaitesii*	Antifungal and Antioxidant activities	[10]
**5**	2,5-dihydroxyxanthone	*Hypericum roeperanum*	Antibacterial Activity	[11]
		Hypericum canariensis		[12]
		Hypericum chinense		[13]
**6**	5-hydroxy-3-methoxyxanthone	*Hypericum riparium*	Antibacterial Activity	[14]
		Hypericum lanceolatum		[15]
**7**	1,7-dihydroxyxanthone	*Hypericum lagarocladum*		[16]
		Garcinia succifolia	EGFR-tyrosine kinase inhibitory activity	[17]
		Garcinia schomburgkiana		[2]
		Hypericum beanii	Anti-staphylococcal	[18]
		Cratoxylum arborescens		[19]
		Mammea siamensis	Cytotoxic efect	[1]
		Cameroonians allanblackia	Antiplasmodial activity	[20]
		Polygala caudata	Antioxidation and vasodilatation activities in vitro	[21]
		Hypericum henryi		[22]
		Garcinia hombroniana	Inhibitory activities on human low-density lipoprotein (LDL)	[23]
		Garcinia cowa		[24]

		Securidaca inappendiculata		[25]
		Garcinia griffithii		[26]
		Kayea assamica		[5]
		indera fruticosa		[27]
		Vismia rubescens	Antimicrobial activities	[28]
		Calophyllum teysmannii		[6]
		Vismia laurentii		[29]
		Allanblackia gabonensis		[30]
		Garcinia dulcis	Antimalarial activity	[31]
		Garcinia multiflora		[32]
		Allanblackia floribunda	Antimicrobial activities	[33]
**8**	2,5-dihydroxy-1-methoxyxanthone	*Garcinia xanthochymus*	Antioxidant	[34]
		Securidaca inappendiculata		[35]
		Garcinia subelliptica		[36]
**9**	1,5-dihydroxy-2-methoxyxanthone	*Hypericum roeperanum*		[7]
**10**	5-hydroxy-1,2-dimethoxyxanthone	*Mammea siamensis*		[37]
**11**	1,3,5-trihydroxyxanthone	*Garcinia schomburgkiana*		[2]
		Swertia patens		[38]
		Garcinia cantleyana	Selective inhibitory activity on platelet aggregation induced by	[39]
		Gentianella acuta	Ameliorate Colorectal Carcinoma	[40]
		Tovomita krukovii		[41]
		Hypericum oblongifolium	Anti-inflammatory	[42]
		Garcinia afzelii	Cytotoxicity	[43]
		Garcinia polyantha	Anticholinesterase activity	[44]
		Garcinia smeathmannii		[45]
**12**	3,7-dihydroxy-1-methoxyxanthone	*Cratoxylum cochinchinense*		[46]
		Cudrania cochinchinensis	Anti-inflammatory	[47]
**13**	1,7-dihydroxy-4-methoxyxanthone	*Polygala karensium*	Inhibit neuraminidases from influenza A viruses	[48]
		Securidaca inappendiculata		[35]

		Cratoxylum maingayi		[49]
		Polygala nyikensis		[50]
**14**	1,6-dihydroxy-7-methoxyxanthone	*Calophyllum inophyllum*	Cytotoxicity	[51]
		Securidaca inappendiculata		[25]
		Hypericum riparium		[14]
**15**	3,4-dihydroxy-2-methoxyxanthone	*Hypericum oblongifolium*		[52]
		Kielmeyera variabilis	Antistaphylococcal	[53]
		Hypericum lagarocladum		[16]
		Hypericum oblongifolium		[52]
**16**	3-hydroxy-2,4-dimethoxyxanthone	*Hypericum acmosepalum*	Cytotoxicity	[54]
		Calophyllum teysmannii		[6]
		Hypericum chinense		[13]
**17**	3,5-dihydroxy-4-methoxyxanthone	*Kielmeyera variabilis*	Antistaphylococcal	[53]
		Tovomita krukovii		[41]
**18**	1,3,5-trihydroxy-2-methoxyxanthone	*Garcinia atroviridis*		[55]
		Pentadesma butyracea		[56]
		Monnina obtusifolia	Antifungal	[57]
**19**	1,3,5,6-tetrahydroxyxanthone	*Garcinia achachairu*	Promotes diuresis, renal protection and antiurolithic	[58]
		Cudrania cochinchinensis		[59]
		Hypericum roeperianum	Cytotoxicity	[60]
		Garcinia Schomburgkian		[61]
		Hypericum perforatum		[62]
		Tripterospermum lanceolatum	Inhibit angiotensin-I-converting enzyme	[63]
		Hypericum erectum	hepatoprotective	[64]
		Garcinia polyantha		[44]
		Garcinia mangostana		[26]
**20**	3,5,6-trihydroxy-1-methoxyxanthone	*Cudrania fruticosa*		[65]
**21**		*Tovomita krukovii*		[41]
		Canscora decussata		[66]

	1,6-dihydroxy-3,5-	*Chironia krebsii*		[67]
**22**	1,3,6,7-tetrahydroxyxanthone	*Gamboge oblongifolia*	Suppress the proliferation of HCC cells	[68]
		Cudrania cochinchinensis		[59]
		Garcinia esculenta	Xanthine oxidase inhibitory	[69]
		Garcinia succifolia	Antibacterial	[17]
		Garcinia mangostana	Cytotoxic and antioxidant activities	[70]
		Garcinia hombroniana	Antioxidant activitie	[71]
		Tripterospermum lanceolatum	Inhibit angiotensin-I-converting enzyme	[63]
		Hippocratea africana	Anti-inflammatory, analgesic, and antioxidant	[72]
		Garcinia brasiliensis	Antioxidant activitie	[73]
**23**	3,6,7-trihydroxy-1-methoxyxanthone	*Cudrania fruticosa*		[65]
		Allanblackia monticola		[74]
**24**	3,6-dihydroxy-	*Hypericum ascyron*		[75]

References

[1] Chaniad, P.; Chukaew, A.; Payaka, A.; Phuwajaroanpong, A.; Techarang, T.; Plirat, W. Punsawad, C. Antimalarial potential of compounds isolated from *Mammea siamensis* T. Anders. flowers: in vitro and molecular docking studies. *BMC complementary medicine and therapies* **2022**, *22*, 266. https://doi.org/10.1186/s12906-022-03742-7

[2] Lien Do, T.M.; Duong, T.H.; Nguyen, V.K.; Phuwapraisirisan, P.; Doungwichitrkul, T.; Niamnont, N.; Jarupinthusophon, S.; Sichaem, J. Schomburgkixanthone, a novel bixanthone from the twigs of *Garcinia schomburgkiana*. *Natural product research* **2021**, *35*, 3613–3618. https://doi.org/10.1080/14786419.2020.1716351

[3] Dang, B.T.; Gény, C.; Blanchard, P.; Rouger, C.; Tonnerre, P.; Charreau, B.; Rakolomalala, G.; Randriamboavonjy, J.I.; Loirand, G.; Pacaud, P.; Litaudon, M.; Richomme, P.; Séraphin, D.; Derbré, S. Advanced glycation inhibition and protection against endothelial dysfunction induced by coumarins and procyanidins from *Mammea neurophylla*. *Fitoterapia* **2014**, *96*, 65–75. https://doi.org/10.1016/j.fitote.2014.04.005

[4] Xiao, Q.; Zeng, Y.B.; Mei, W.L.; Zhao, Y.X.; Deng, Y.Y.; Dai, H.F. Cytotoxic prenylated xanthones from *Calophyllum inophyllum. Journal of Asian natural products research* **2008**, *10*(9-10), 993–997. https://doi.org/10.1080/10519990802240387

[5] Lee, K.H.; Chai, H.B.; Tamez, P.A.; Pezzuto, J.M.; Cordell, G.A.; Win, K.K.; Tin-Wa, M. Biologically active alkylated coumarins from *Kayea assamica*. *Phytochemistry* **2003**, 64, 535–541. https://doi.org/10.1016/s0031-9422(03)00243-7

[6] Kijjoa, A.; Gonzalez, M.J.; Pinto, M.M.; Silva, A.M.; Anantachoke, C.; Herz, W. Xanthones from *Calophyllum teysmannii* var. inophylloide. *Phytochemistry* **2000**, 55, 833–836. https://doi.org/10.1016/s0031-9422(00)00289-2

[7] Rath, G.; Potterat, O.; Mavi, S.; Hostettmann, K. Xanthones from *Hypericum roeperanum*. *Phytochemistry*, **1996**, 43, 513–520. https://doi.org/10.1016/0031-9422(96)00284-1

[8] Zhao, H.; Wang, X.; Li, W.; Koike, K.; Bai, H. A new minor homoisoflavonoid from *Caesalpinia sappan*. *Natural product research* **2014**, *28*, 102–105. https://doi.org/10.1080/14786419.2013.847439

[9] Habib, A.M.; Reddy, K.S.; McCloud, T.G.; Chang, C.J.; Cassady, J.M. New xanthones from *Psorospermum febrifugum*. *Journal of natural products* **1987**, *50*, 141–145. https://doi.org/10.1021/np50050a001

[10] Dharmaratne, H.R.; Napagoda, M.T.; Tennakoon, S.B.; Xanthones from roots of *Calophyllum thwaitesii* and their bioactivity. *Natural product research* **2009**, 23, 539–545. https://doi.org/10.1080/14786410600899118

[11] Kamga, P.B.; Mouthe, G.L.T.; Wache, B.O.; Beng, V.P.; Nkengfack, A.E. Antibacterial activity of the ethyl acetate extract of *Hypericum roeperanum* schimp. ex a. rich. (guttifereae)

and their secondary metabolitecs. *Pharmacologia* **2012**, 3, 632-636. https://doi.org/10.5567/pharmacologia.2012.632.636

[12] Cardona M.L.; Pedro J.R.; Seoane E.; Vidal R. Xanthone constituents of *Hypericum canariensis*. *J. Nat. Prod.* **1985**, 48, 467–469. https://doi.org/10.1021/np50039a018.

[13] Tanaka, N.; Takaishi, Y. Xanthones from Hypericum chinense. *Phytochemistry* **2006**, 67, 2146–2151. https://doi.org/10.1016/j.phytochem.2006.05.043

[14] Michel F.T.; Patricia D.T.; Hippolyte K.W.; Ferdinand M.T.; Pierre T.; Jules R.K.; Léon A.T.; Hartmut L. Chemical constituents, antimicrobial and cytotoxic activities of *Hypericum riparium* (guttiferae). *Records of Natural Products* **2013**, 7, 65–68.

[15] Hippolyte K.W.; Théodora K.K.; Antoine Honoré N.L.; Alembert T.T.; Pierre T.; Haruhisa K.; Michel F.; Yoshiteru O. Phenolic Compounds and Terpenoids from *Hypericum lanceolatum*. *Records of Natural Products* **2012**, 6, 94-100.

[16] Deng, J.T.; Zhou, T.X.; Yang, Y.C.; Han, Q.D.; Cheng, H.T.; Wang, Q.; & Yang, X.Z. A new xanthone from *Hypericum lagarocladum. China journal of Chinese materia medica* **2022**, 47, 5544–5549. https://doi.org/10.19540/j.cnki.cjcmm.20220418.202

[17] Duangsrisai, S.; Choowongkomon, K.; Bessa, L.J.; Costa, P.M.; Amat, N.; Kijjoa, A. Antibacterial and EGFR-tyrosine kinase inhibitory activities of polyhydroxylated xanthones from *Garcinia succifolia*. *Molecules* **2014**, 19, 19923–19934. https://doi.org/10.3390/molecules191219923

[18] Shiu, W.K.; Gibbons, S. Anti-staphylococcal acylphloroglucinols from *Hypericum beanii*. *Phytochemistry* **2006**, 67, 2568–2572. https://doi.org/10.1016/j.phytochem.2006.09.037

[19] Pattanaprateeb, P.; Ruangrungsi, N.; Cordell, G.A. Cytotoxic constituents from Cratoxylum arborescens. *Planta medica* **2005**, 71, 181–183. https://doi.org/10.1055/s-2005-837788

[20] Azebaze, A.G.; Teinkela, J.E.; Nguemfo, E.L.; Valentin, A.; Dongmo, A.B.; Vardamides, J.C. Antiplasmodial activity of some phenolic compounds from *Cameroonians Allanblackia*. *African health sciences* **2015**, 15, 835–840. https://doi.org/10.4314/ahs.v15i3.18

[21] Lin, L.L.; Huang, F.; Chen, S.B.; Yang, D.J.; Chen, S.L.; Yang, J.S.; & Xiao, P.G. Xanthones from the roots of *Polygala caudata* and their antioxidation and vasodilatation activities in vitro. *Planta medica* **2005**, 71, 372–375. https://doi.org/10.1055/s-2005-864108

[22] Wu, Q.L.; Wang, S.P.; Du, L.J.; Yang, J.S.; Xiao, P.G. Xanthones from *Hypericum japonicum* and *H. henryi*. *Phytochemistry* **1998**, 49, 1395–1402. https://doi.org/10.1016/s0031-9422(98)00116-2

[23] Saputri, F.C.; Jantan, I. Inhibitory activities of compounds from the twigs of *Garcinia hombroniana* Pierre on human low-density lipoprotein (LDL) oxidation and platelet aggregation. *Phytotherapy research: PTR* **2012**, 26, 1845–1850. https://doi.org/10.1002/ptr.4667

[24] Shen, J.; Yang, J. S. Two new xanthones from the stems of *Garcinia cowa*. *Chemical & pharmaceutical bulletin* **2006**, 54, 126–128. https://doi.org/10.1248/cpb.54.126

[25] Wang, Q.W.; Ma, C.Y.; Chen, D.D.; Li, X.; Chen, J.W. Xanthones from Rhizoma of *Securidaca inappendiculata*. *Journal of Chinese medicinal materials* **2016,** 39, 2024–2026.

[26] Nguyen, L.H.; Venkatraman, G.; Sim, K.Y.; Harrison, L.J. Xanthones and benzophenones from *Garcinia griffithii* and *Garcinia mangostana*. *Phytochemistry* **2005**, 66, 1718–1723. https://doi.org/10.1016/j.phytochem.2005.04.032

[27] Song, M.C.; Nigussie, F.; Jeong, T.S.; Lee, C.Y.; Regassa, F.; Markos, T.; Baek, N.I. Phenolic compounds from the roots of *Lindera fruticosa*. *Journal of natural products* **2006**, 69, 853–855. https://doi.org/10.1021/np060048b

[28] Tamokou, J.D.; Tala, M.F.; Wabo, H.K.; Kuiate, J.R.; Tane, P. Antimicrobial activities of methanol extract and compounds from stem bark of Vismia rubescens. *Journal of ethnopharmacology* **2009**, 124, 571–575. https://doi.org/10.1016/j.jep.2009.04.062

[29] Nguemeving, J.R.; Azebaze, A.G.; Kuete, V.; Eric Carly, N.N.; Beng, V.P.; Meyer, M.; Blond, A.; Bodo, B.; Nkengfack, A.E. Laurentixanthones A and B, antimicrobial xanthones from Vismia laurentii. *Phytochemistry* **2006**, 67, 1341–1346. https://doi.org/10.1016/j.phytochem.2006.03.018

[30] Azebaze, A.G.; Ouahouo, B.M.; Vardamides, J.C.; Valentin, A.; Kuete, V.; Acebey, L.; Beng, V.P.; Nkengfack, A.E.; Meyer, M.. Antimicrobial and antileishmanial xanthones from the stem bark of Allanblackia gabonensis (Guttiferae). *Natural product research* **2010**, 24, 781. https://doi.org/10.1080/14786419.2010.486529

[31] Likhitwitayawuid, K.; Chanmahasathien, W.; Ruangrungsi, N.; Krungkrai, J. Xanthones with antimalarial activity from Garcinia dulcis. *Planta medica* **1998**, 64, 281–282. https://doi.org/10.1055/s-2006-957429

[32] Jing, W.Y.; Jiang, C.; Ji, F.; Hua, H.M.; Li, Z.L. Chemical constituents from the stem barks of Garcinia multiflora. *Journal of Asian natural products research* **2013**, 15, 1152–1157. https://doi.org/10.1080/10286020.2013.819854

[33] Kuete, V.; Azebaze, A.G.; Mbaveng, A.; Nguemfo, E.L.; Tshikalange, E.T.; Chalard, P.; Nkengfack, A.E. Antioxidant, antitumor and antimicrobial activities of the crude extract and compounds of the root bark of Allanblackia floribunda. *Pharmaceutical biology* **2011**, 49, 57–65. https://doi.org/10.3109/13880209.2010.494673

[34] Cheng W.Y.; Zhong F.F.; Zhao Y.H.; Yang G.Z.; Chen Yu. Study on the Antioxidant Constituents from the Barks of *Garcinia xanthochymus*. *Natural Product Research and Development* **2008**, 20, 836-838.

[35] Yang, X.D.; Xu, L.Z.; Yang, S.L. Xanthones from the stems of *Securidaca inappendiculata*. *Phytochemistry* **2001**, 58, 1245–1249. https://doi.org/10.1016/s0031-9422(01)00356-9

[36] Hiroyuki M.; Emi T.; Mitsuaki K.; Yoshiyasu F.; Three xanthones from *Garcinia subelliptica*. *Phytochemistry* **1996**, 41, 629-633. https://doi.org/10.1016/0031-9422(95)00567-6

[37] Poobrasert, O.; Constant, H.L.; Beecher, C.W.; Farnsworth, N.R.; Kinghorn, A.D.; Pezzuto, J.M.; Cordell, G.A.; Santisuk, T.; Reutrakul, V. Xanthones from the twigs of Mammea siamensis. *Phytochemistry* **1998**, 47, 1661–1663. https://doi.org/10.1016/s0031-9422(97)00820-0

[38] He, K.; Cao, T.W.; Wang, H.L.; Geng, C.A.; Zhang, X.M.; Chen, J.J. Chemical constituents of *Swertia patens. China journal of Chinese materia medica*, **2015**, 40, 4012–4017.

[39] Jantan, I.; Saputri, F.C. Benzophenones and xanthones from *Garcinia cantleyana* var. cantleyana and their inhibitory activities on human low-density lipoprotein oxidation and platelet aggregation. *Phytochemistry* **2012**, 80, 58–63. https://doi.org/10.1016/j.phytochem.2012.05.003

[40] Lu, M.Q.; Ruan, J.Y.; Li, H.M.; Yang, D.S.; Liu, Y.X.; Hao, M.M.; Yu, H.Y.; Zhang, Y.; Wang, T. Xanthones from *Gentianella acuta* (Michx.) Hulten Ameliorate Colorectal Carcinoma via the PI3K/Akt/mTOR Signaling Pathway. *International journal of molecular sciences* **2023**, 24, 2279. https://doi.org/10.3390/ijms24032279

[41] Zhang, Z.; ElSohly, H.N.; Jacob, M.R.; Pasco, D.S.; Walker, L.A.; Clark, A.M. Natural products inhibiting Candida albicans secreted aspartic proteases from *Tovomita krukovii*. *Planta medica* **2002**, 68, 49–54. https://doi.org/10.1055/s-2002-20049

[42] Ali, M.; Arfan, M.; Ahmad, M.; Singh, K.; Anis, I.; Ahmad, H.; Choudhary, M. I.; Shah, M.R. Anti-inflammatory xanthones from the twigs of *Hypericum oblongifolium* wall. *Planta medica* **2011**, 77, 2013–2018. https://doi.org/10.1055/s-0031-1280114

[43] Lannang, A.M.; Louh, G.N.; Biloa, B.M.; Komguem, J.; Mbazoa, C.D.; Sondengam, B.L.; Naesens, L.; Pannecouque, C.; De Clercq, E.; Sayed El Ashry, H. Cytotoxicity of natural compounds isolated from the seeds of *Garcinia afzelii*. *Planta medica* **2010**, 76, 708–712. https://doi.org/10.1055/s-0029-1240627

[44] Louh, G.N.; Lannang, A.M.; Mbazoa, C.D.; Tangmouo, J.G.; Komguem, J.; Castilho, P.; Ngninzeko, F.N.; Qamar, N.; Lontsi, D.; Choudhary, M.I.; Sondengam, B.L. Polyanxanthone A, B and C, three xanthones from the wood trunk of *Garcinia polyantha* Oliv. *Phytochemistry* **2008**, 69, 1013–1017. https://doi.org/10.1016/j.phytochem.2007.10.002

[45] Komguem, J.; Meli, A.L.; Manfouo, R.N.; Lontsi, D.; Ngounou, F.N.; Kuete, V.; Kamdem, H.W.; Tane, P.; Ngadjui, B.T.; Sondengam, B.L.; Connolly, J.D. Xanthones from Garcinia smeathmannii (Oliver) and their antimicrobial activity. *Phytochemistry* **2005**, 66, 1713–1717. https://doi.org/10.1016/j.phytochem.2005.03.010

[46] Duan, Y.; Dai, Y.; Chen, L.; Liu, M.; Li, Y.; Yao, X. Xanthones from the stems of *Cratoxylum cochinchinense*. *Magnetic resonance in chemistry MRC* **2012**, 50, 642–645. https://doi.org/10.1002/mrc.3852

[47] Lin, C.F.; Chen, Y.J.; Huang, Y.L.; Chiou, W.F.; Chiu, J.H.; Chen, C.C. A new auronol from *Cudrania cochinchinensis*. *Journal of Asian natural products research* **2012**, 14, 704–707. https://doi.org/10.1080/10286020.2012.682305

[48] Dao, T.T.; Dang, T.T.; Nguyen, P.H.; Kim, E.; Thuong, P.T.; OH, W.K. Xanthones from *Polygala karensium* inhibit neuraminidases from influenza A viruses. *Bioorganic & medicinal chemistry letters* **2012**, 22, 3688–3692. https://doi.org/10.1016/j.bmcl.2012.04.028

[49] Kijjoα, A.; José, M.; Gonzalez, T.G.; Madalena M.M.; Pinto, A.M.; Werner H. Xanthones from *Cratoxylum maingayi Phytochemistry* **1998**, 49, 2159-2162. https://doi.org/10.1016/S0031-9422(98)00381-1

[50] Marston, A.; Hamburger, M.; Sordat-Diserens, I.; Msonthi, J.D.; Hostettmann, K. Xanthones from *Polygala nyikensis*. *Phytochemistry* **1993**, 33, 809-812. https://doi.org/10.1016/0031-9422(93)85279-Z

[51] Ponguschariyagul, S.; Sichaem, J.; Khumkratok, S.; Siripong, P.; Lugsanangarm, K.; Tip-Pyang, S. Caloinophyllin A, a new chromanone derivative from *Calophyllum inophyllum* roots. *Natural product research* **2018**, 32, 2535–2541. https://doi.org/10.1080/14786419.2018.1425845

[52] Ali, M.; Latif, A.; Zaman, K.; Arfan, M.; Maitland, D.; Ahmad, H.; Ahmad, M. Anti-ulcer xanthones from the roots of *Hypericum oblongifolium* Wall. *Fitoterapia* **2014**, 95, 258–265. https://doi.org/10.1016/j.fitote.2014.03.014

[53] Coqueiro, A.; Choi, Y.H.; Verpoorte, R.; Gupta, K.B.; De Mieri, M.; Hamburger, M.; Young, M.C.; Stapleton, P.; Gibbons, S.; Bolzani, V.S. Antistaphylococcal Prenylated Acylphoroglucinol and Xanthones from *Kielmeyera variabilis*. *Journal of natural products* **2016**, 79, 470–476. https://doi.org/10.1021/acs.jnatprod.5b00858

[54] Wang, A.Z.; Fang, Q.Q.; Feng, T.T.; Wei, R.J.; Jiang, K.; Lu, Q.; Tan, C.H.. Acmoxanthones A-E, New Lavandulated Xanthones from *Hypericum acmosepalum* N. Robson. *Fitoterapia* **2021**, *154*, 104923. https://doi.org/10.1016/j.fitote.2021.104923

[55] Tan, W.N.; Khairuddean, M.; Wong, K.C.; Tong, W.Y.; Ibrahim, D. Antioxidant compounds from the stem bark of Garcinia atroviridis. *Journal of Asian natural products research* **2016**, 18, 804–811. https://doi.org/10.1080/10286020.2016.1160071

[56] Lenta, B.N.; Kamdem, L.M.; Ngouela, S.; Tantangmo, F.; Devkota, K.P.; Boyom, F.F.; Rosenthal, P.J.; Tsamo, E. Antiplasmodial constituents from the fruit pericarp of *Pentadesma butyracea*. *Planta medica* **2011**, 77, 377–379. https://doi.org/10.1055/s-0030-1250384

[57] Pinto, D.C.; Fuzzati, N.; Pazmino, X.C.; Hostettmann, K. Xanthone and antifungal constituents from *Monnina obtusifolia*. *Phytochemistry* **1994**, 37, 875–878. https://doi.org/10.1016/s0031-9422(00)90375-3

[58] Mariano, L.N.B.; Boeing, T.; Cechinel Filho, V.; Niero, R.; Mota da Silva, L.; Souza, P. 1,3,5,6-tetrahydroxyxanthone promotes diuresis, renal protection and antiurolithic properties in normotensive and hypertensive rats. *The Journal of pharmacy and pharmacology* **2021**, 73, 700–708. https://doi.org/10.1093/jpp/rgab026

[59] Chen, L.; Zhou, Q.; Li, B.; Liu, S.J.; Dong, J.X. A new flavonoid from *Cudrania cochinchinensis*. *Natural product research* **2015**, 29, 1217–1221. https://doi.org/10.1080/14786419.2014.997234

[60] Guefack, M.F.; Damen, F.; Mbaveng, A.T.; Tankeo, S.B.; Bitchagno, G.T.M.; Çelik, İ.; Simo Mpetga, J.D.; Kuete, V. Cytotoxic Constituents of the Bark of *Hypericum roeperianum* towards Multidrug-Resistant Cancer Cells. *Evidence-based complementary and alternative medicine: Ecam* **2020**, 2020, 4314807. https://doi.org/10.1155/2020/4314807

[61] Meechai, I.; Phupong, W.; Chunglok, W.; Meepowpan, P. Dihydroosajaxanthone: A New Natural Xanthone from the Branches of *Garcinia Schomburgkiana* Pierre. *Iranian journal of pharmaceutical research: IJPR* **2018**, 17, 1347–1352.

[62] Ferrari, F.; Pasqua, G.; Monacelli, B.; Cimino, P.; Botta, B. Xanthones from calli of *Hypericum perforatum* subsp. perforatum. *Natural product research* **2005**, 19, 171–176. https://doi.org/10.1080/14786410410001704796

[63] Chen, C.H.; Lin, J.Y.; Lin, C.N.; Hsu, S. Y. Inhibition of angiotensin-I-converting enzyme by tetrahydroxyxanthones isolated fromI. *Journal of natural products* **1992**, 55, 691–695. https://doi.org/10.1021/np50083a025

[64] An, R.B.; Jeong, G.S.; Beom, J.S.; Sohn, D.H.; Kim, Y.C. Chromone glycosides and hepatoprotective constituents of *Hypericum erectum*. *Archives of pharmacal research* **2009**, 32, 1393–1397. https://doi.org/10.1007/s12272-009-2008-1

[65] Liang, B.; Li, H.R.; Xu, L.Z., Yang, S. L. Xanthones from the roots of *Cudrania fruticosa* Wight. *Journal of Asian natural products research* **2007**, 9, 393–397. https://doi.org/10.1080/10286020600782355

[66] Ghosal S.; Chaudhuri R.K.; Nath A. Chemical constituents of gentianaceae IV: New xanthones of *Canscora decussata*. *J. Pharm. Sci.* **1973**, 62, 137–139. https://doi.org/10.1002/jps.2600620128.

[67] Wolfender J.L.; Hamburger M.; Msonthi J.D.; Hostettmann K. Xanthones from *Chironia krebsii*. *Phytochemistry* **1991**, 30, 3625–3629. https://doi.org/10.1016/0031-9422(91)80080-K.

[68] Fu, W.M.; Zhang, J.F.; Wang, H.; Tan, H.S.; Wang, W.M.; Chen, S.C.; Zhu, X.; Chan, T.M.; Tse, C.M.; Leung, K.S.; Lu, G.; Xu, H.X.; Kung, H.F. Apoptosis induced by 1,3,6,7-tetrahydroxyxanthone in *Hepatocellular carcinoma* and proteomic analysis. *Apoptosis: an*

international journal on programmed cell death **2012**, 17, 842–851. https://doi.org/10.1007/s10495-012-0729-y

[69] Zhu, L.L.; Fu, W.W.; Watanabe, S.; Shao, Y.N.; Tan, H.S.; Zhang, H.; Tan, C.H.; Xiu, Y.F.; Norimoto, H.; Xu, H.X. Xanthine oxidase inhibitors from *Garcinia esculenta* twigs. *Planta medica* **2014**, 80, 1721–1726. https://doi.org/10.1055/s-0034-1383193

[70] Tran, T.H.; Nguyen, V.T.; Le, H.T.; Nguyen, H.M.; Tran, T.H.; Do Thi, T.; Nguyen, X.C.; Ha, M.T. Garcinoxanthones SV, new xanthone derivatives from the pericarps of *Garcinia mangostana* together with their cytotoxic and antioxidant activities. *Fitoterapia* **2021**, 151, 104880. https://doi.org/10.1016/j.fitote.2021.104880

[71] Jamila, N.; Khairuddean, M.; Yaacob, N.S.; Kamal, N.N.; Osman, H.; Khan, S.N.; Khan, N. Cytotoxic benzophenone and triterpene from Garcinia hombroniana. *Bioorganic chemistry* **2014**, 54, 60–67. https://doi.org/10.1016/j.bioorg.2014.04.003

[72] Umoh, U.F.; Thomas, P.S.; Essien, E.E.; Okokon, J.E.; De Leo, M.; Ajibesin, K.K.; Flamini, G.; Eseyin, O.A. Isolation and characterization of bioactive xanthones from *Hippocratea africana* (Willd.) Loes.ex Engl. (Celastraceae). *Journal of ethnopharmacology* **2021**, 280, 114031. https://doi.org/10.1016/j.jep.2021.114031

[73] Gontijo, V.S.; Souza, T.C.; Rosa, I.A.; Soares, M.G.; Silva, M.A.; Vilegas, W.; Viegas, C.; Dos Santos, M.H. Isolation and evaluation of the antioxidant activity of phenolic constituents of the *Garcinia brasiliensis* epicarp. *Food chemistry* **2012**, 132, 1230–1235. https://doi.org/10.1016/j.foodchem.2011.10.110

[74] Ngouela, S.; Zelefack, F.; Lenta, B.N.; Ngouamegne, E.T.; Tchamo, D.N.; Tsamo, E.; Connolly, J.D. Xanthones and other constituents of *Allanblackia monticola* (Guttiferae). *Natural product research* **2005**, 19, 685–688. https://doi.org/10.1080/14786410512331330549

[75] Hu L.H.; Yip S.C.; Sim K.Y. Xanthones from *Hypericum ascyron*. *Phytochemistry* **1999**, 52, 1371–1373. https://doi.org/10.1016/S0031-9422(99)00412-4.
